# Supplementary figures and images for: Who bears the cost of forest conservation?
Source: PeerJ. 2018 Jul 5;6:e5106. doi: 10.7717/peerj.5106 (PMC6035863; doi:10.7717/peerj.5106)

Inherited Cleared Bought Rented Borrowed

Percentage of plots

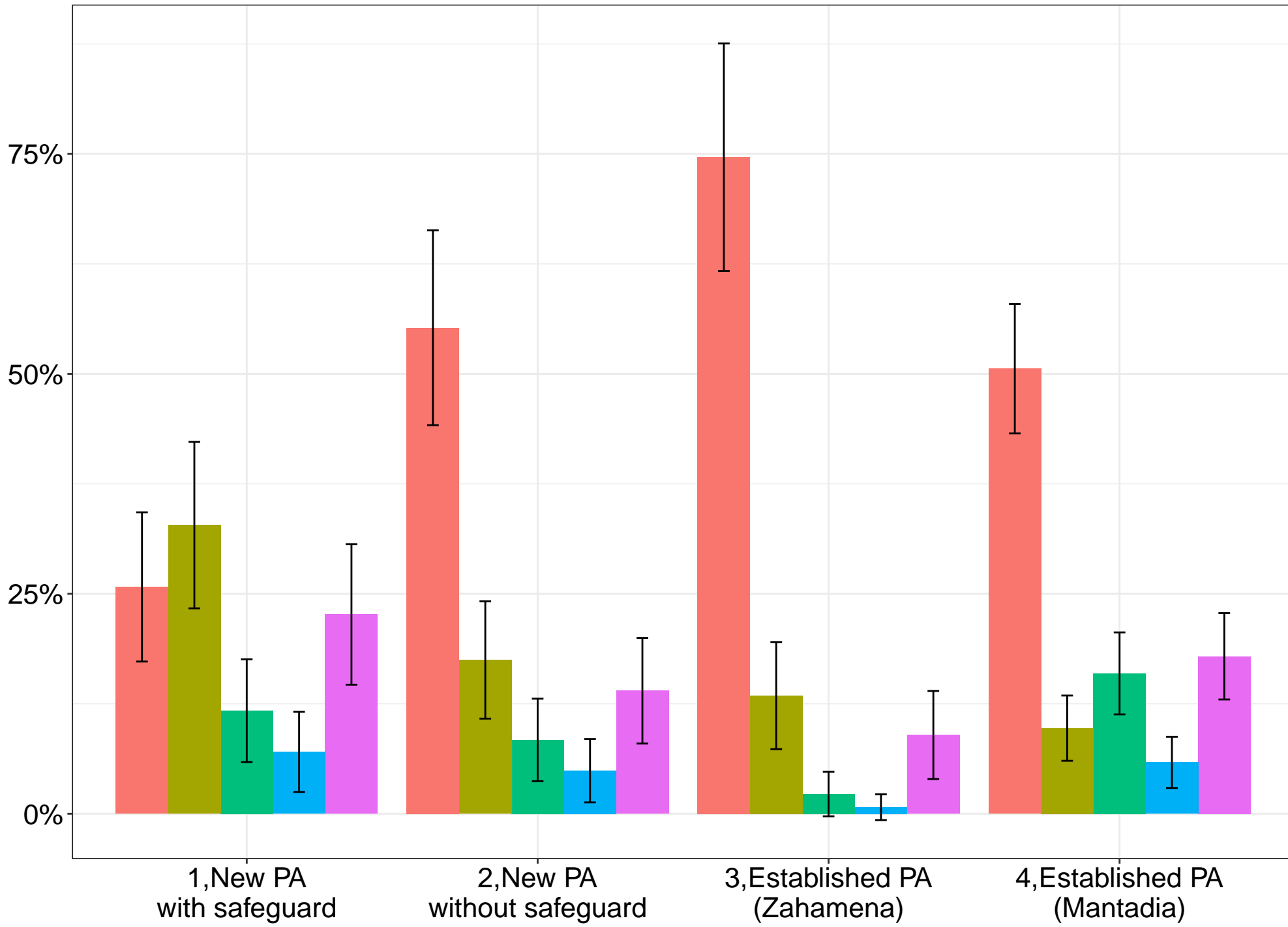

Supplement: Supplemental Information 1 — Y-axis shows the overall percentage of plots in each site being accessed through one of the five ways listed–total adding up to 100% for each site. Error bars show 95% confidence intervals. [file peerj-06-5106-s001.pdf]

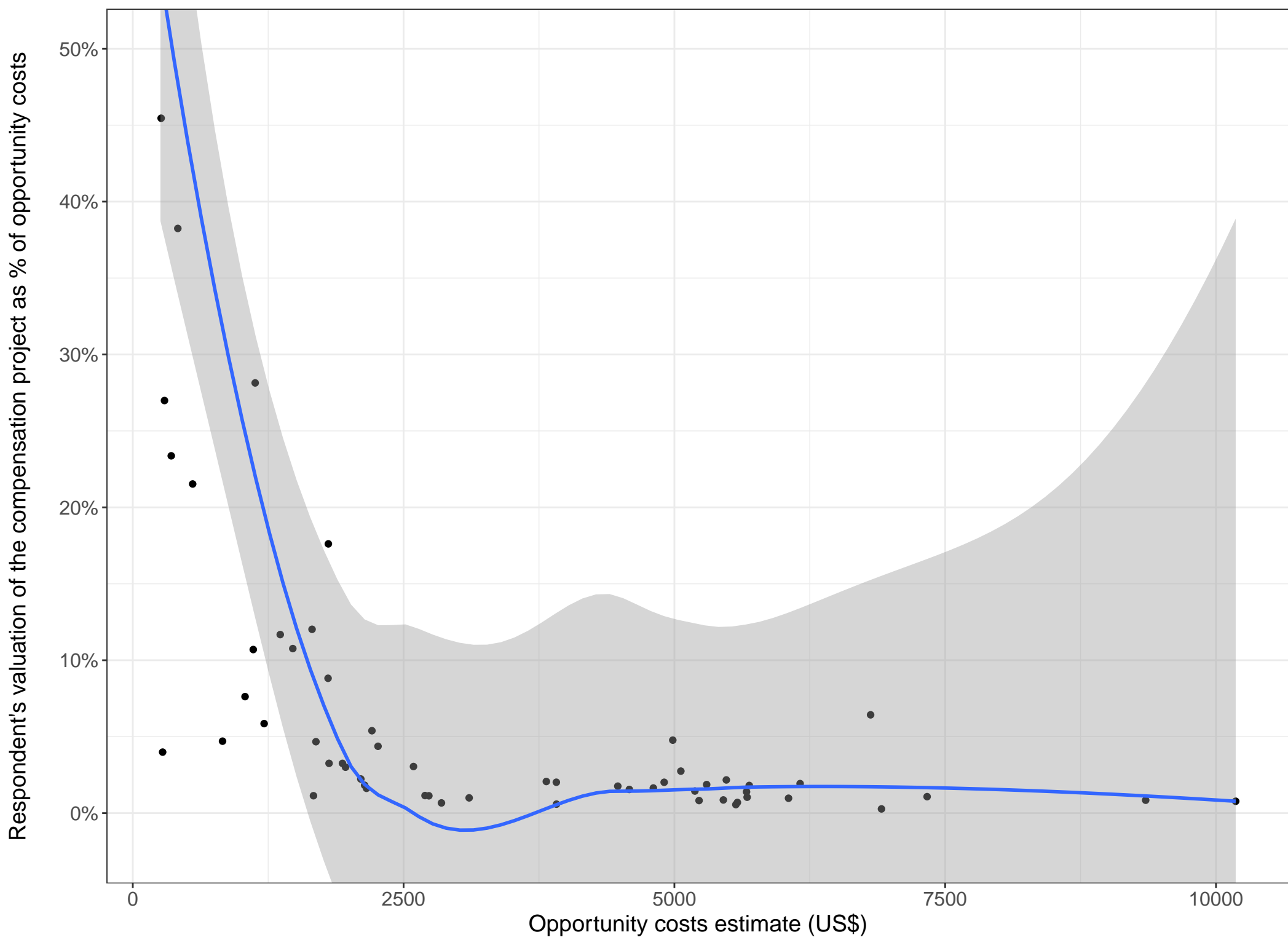

Supplement: Supplemental Information 2 — Data is for 62 recipients of compensation (all from site 1), 2 years after compensation was received. The value of compensation is estimated from our contingent valuation while the opportunity cost of conservation is estimated from the choice experiment. [file peerj-06-5106-s002.pdf]

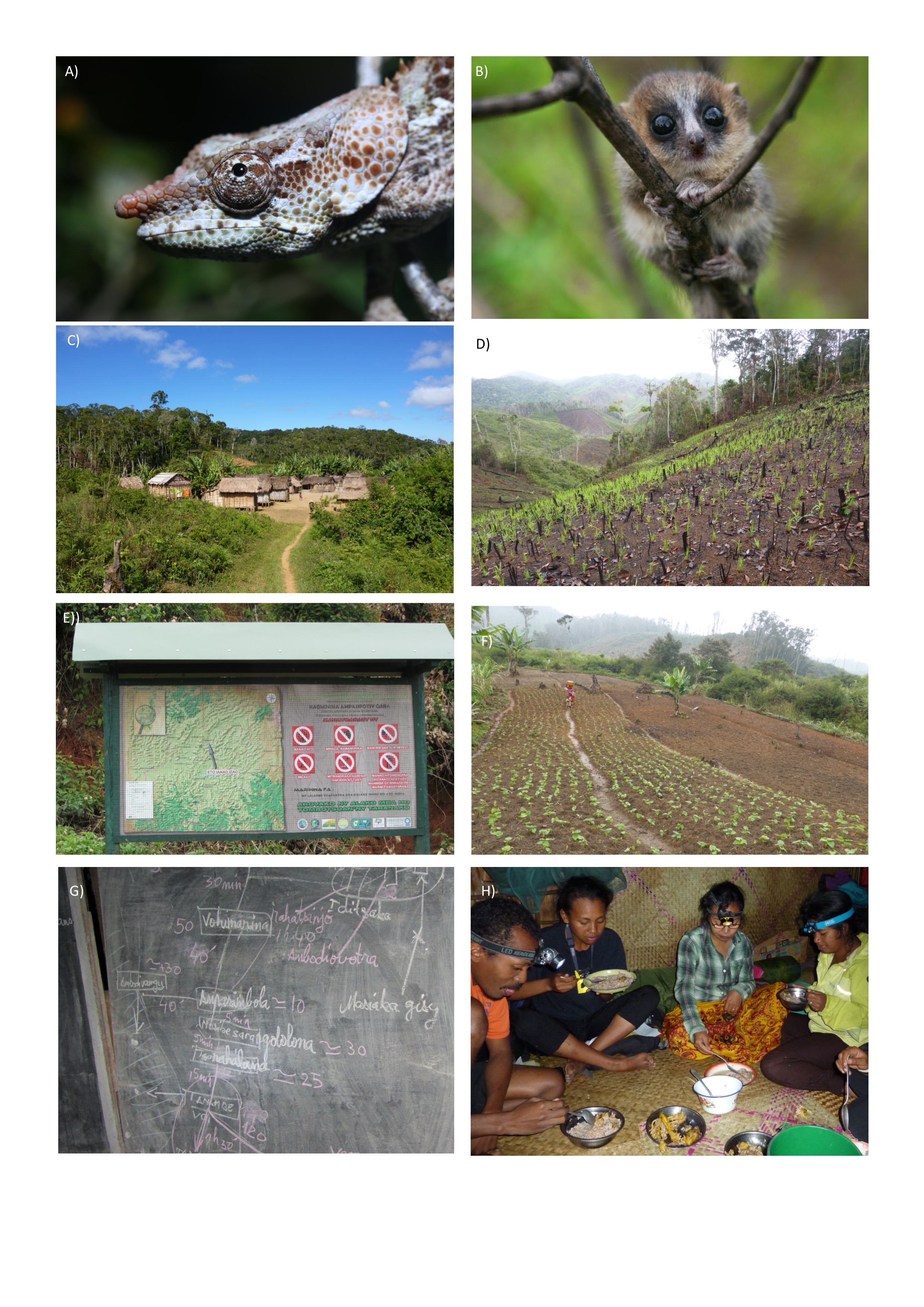

Supplement: Supplemental Information 3 — a), b) The biodiversity of the CAZ is world-renowned. c), d) 10s of 1000s of people live around the CAZ new protected area, traditionally most people depend economically on clearing land for agriculture in a swidden system known locally as ‘tavy.’ e) The CAZ protected area will result in strict enforcement of conservation rules including not clearing new land. f) Selected residents have been identified as Project Affected Person and therefore have received micro-development projects such as improved bean cultivation under World Bank safeguards. g) To build a sampling frame we worked with local leader to update available maps and then visited each hamlet with a GPS. h) Our team stayed in the villages (with local families) where worked for extended periods which facilitated trust. [file peerj-06-5106-s003.jpg]

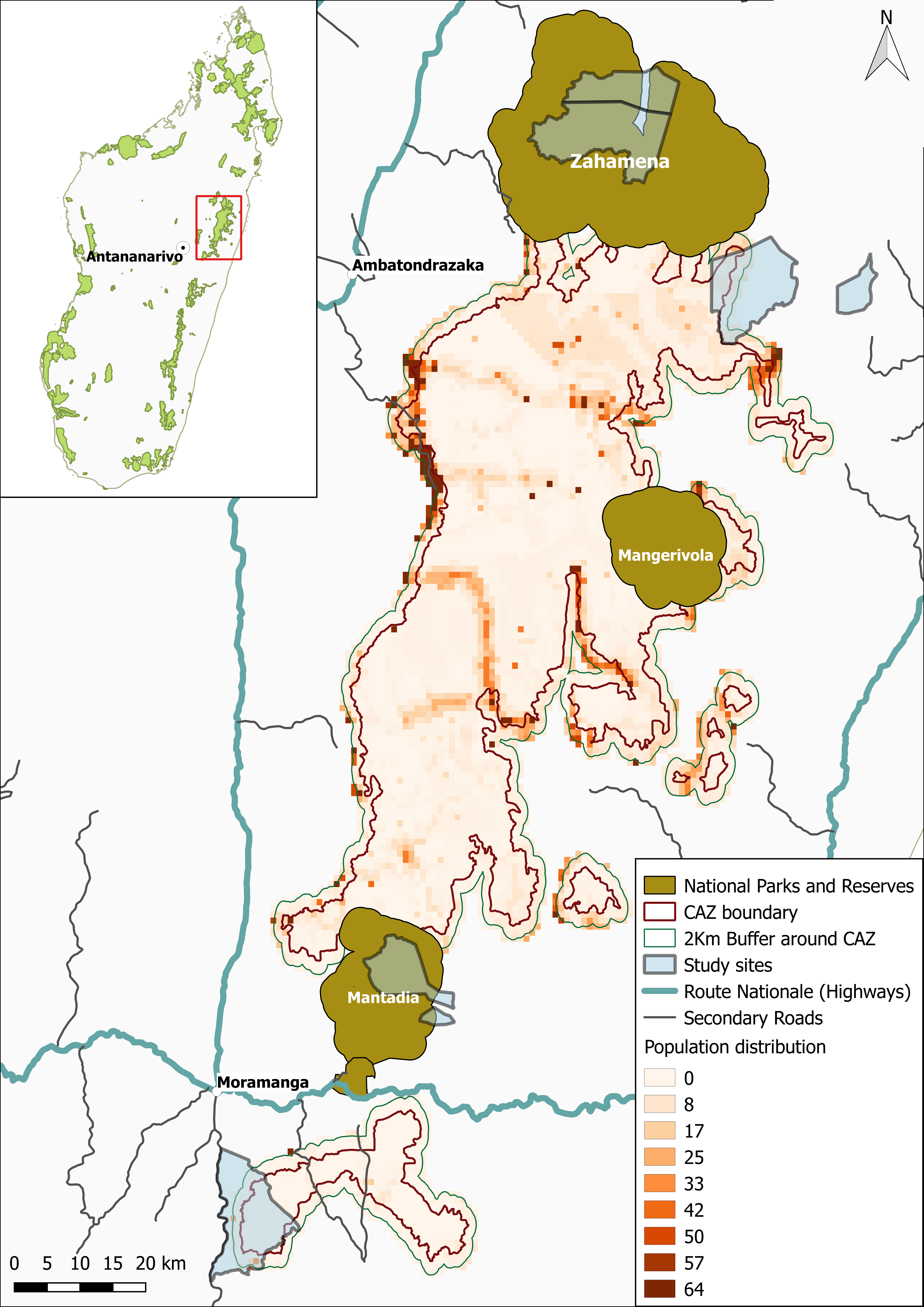

Supplement: Supplemental Information 4 — Established protected areas managed by Madagascar National Parks (with 2 Km buffer around them) have been excluded as different compensation right exist there. The population model is based on Landscan 2007 data distributed with the EcoEngine algorithm in WaterWorld. [file peerj-06-5106-s004.png]

Distributed population data in study sites

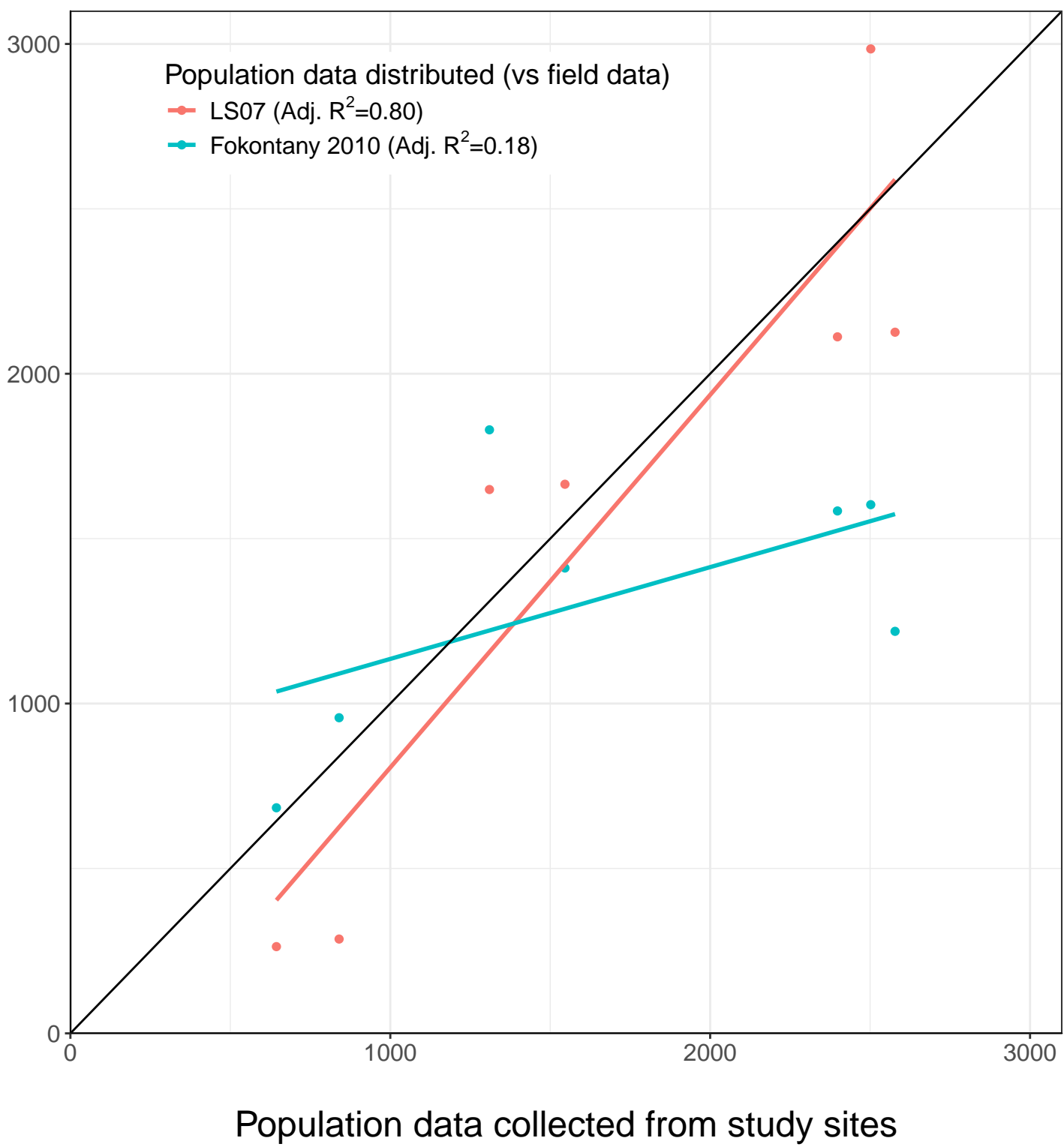

Supplement: Supplemental Information 5 — Our field data shows the primary census data collected in each fokontany during 2014/2015 (population data from p4ges field sites) plotted against the population estimates for those sites from LandScan 2007 (LS07), and INSTAT (Madagascar’s National Institute of Statistics) 2010 data (fokontany 2010)- both distributed using EcoEngine algorithm in WaterWorld. [file peerj-06-5106-s005.pdf]
