# Supplementary material for: Who bears the cost of forest conservation?
Source: PeerJ. 2018 Jul 5;6:e5106. doi: 10.7717/peerj.5106 (PMC6035863; doi:10.7717/peerj.5106)
Supplement: Supplemental Information 6 [file peerj-06-5106-s006.pdf]

| ALTERNATIVES | A | B | C (reference level) |
|---|---|---|---|
| 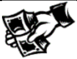 Total cash payments ($10^6$ MGA) | $3 \times 10^6$ 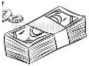 | 6 x $x\ 10^6$ 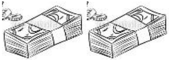 | NONE |
| 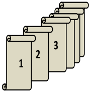 Number of installments | **10** 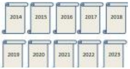 | **20** 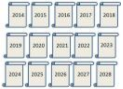 | - |
| 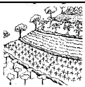 Technical rice farming | **YES** ✓ | **NO** X | **NO** X |
| 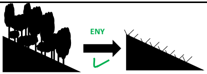  Teviala option | YES BUT only on **1ha** | NO X | YES ✓ |
| **Choice** | ☐ | ☐ | ☐ |
