# Supplementary material for: Who bears the cost of forest conservation?
Source: PeerJ. 2018 Jul 5;6:e5106. doi: 10.7717/peerj.5106 (PMC6035863; doi:10.7717/peerj.5106)
Supplement: Supplemental Information 7 [file peerj-06-5106-s007.docx]

##### Table S1: Attributes and levels of the choice experiment (reference levels in bold).

| **Attributes** | **Description** | **Levels** | **Coding and Notation** |
| --- | --- | --- | --- |
| Total cash donations framed as development assistance (2702 ariary = 1 US$) | The payment levels were selected based on a review of secondary data and previous literature estimating the local costs of deforestation as well as extensive piloting (e.g., refs. *97, 114*). | **0**, 3, 6, 9, 12, 15 (x10^6^ ariary) | Cash (continuous variable) |
| Number of annual instalments | The three levels of instalments allow an estimation of the respondents’ discount rates, and provides information on their ability to invest money. | **1**, 10,20 | Dummy-coded: Installment10 and installment20 |
| Support for improved rice farming | This attribute (based on real micro development interventions in the region) is introduced as an agricultural support package including training and material support for taking up new practices including the use of improved seed, fertilizers, insecticides and the construction of terraces for slopes). | **No,** yes | Dummy-coded: Support for improved rice farming coded as 1 |
| Clearance of forestlands for agriculture | This attribute has three levels: i) free clearance (unrestricted forest clearance for swidden agriculture (similar to pre-colonial times before criminalization of swidden agriculture, and de facto to more recent periods of little or no enforcement). ii) a permit for one hectare of forest clearance, one-off, for swidden agriculture), iii) no forest clearance (i.e. strict conservation). | **Free clearance** (open forest frontier), 1ha clearance permit, and no clearance (strict conservation) | Dummy-coded: 1ha clearance, strict conservation |
