# Supplementary material for: Who bears the cost of forest conservation?
Source: PeerJ. 2018 Jul 5;6:e5106. doi: 10.7717/peerj.5106 (PMC6035863; doi:10.7717/peerj.5106)
Supplement: Supplemental Information 8 [file peerj-06-5106-s008.docx]

**S2. The coefficients from the choice experiment. Lower and upper bounds are 95% confidence intervals**

| **Group** | **Coefficient** | **Estimate** | **Std. Error** | **t-value** | **P Value** | **Lower** | **Upper** |
| --- | --- | --- | --- | --- | --- | --- | --- |
| Main effects for REDD+ project with safeguard | asc | -1076 | 432.8 | -920.2 | 0.013 | -1924 | -228 |
|  | Instalment 10 | 1468 | 250.2 | 2172.0 | 0.000 | 978 | 1958 |
|  | Instalment 20 | 353 | 246.2 | 530.1 | 0.152 | -130 | 835 |
|  | Rice project | 3146 | 505.0 | 2305.8 | 0.000 | 2157 | 4136 |
|  | 1 hectare clearance | 71 | 506.9 | 51.8 | 0.889 | -923 | 1065 |
|  | Strict conservation | -700 | 1733.8 | -149.5 | 0.686 | -4099 | 2698 |
| Rice project interaction | Established PA (Mantadia) | -3654 | 654.3 | -2066.8 | 0.000 | -4936 | -2371 |
|  | REDD+ project without safeguard | 1085 | 887.8 | 452.1 | 0.222 | -656 | 2825 |
|  | Established PA (Zahamena) | -1067 | 599.7 | -658.3 | 0.075 | -2242 | 109 |
|  | Distance from forest (km) | -206 | 148.1 | -515.0 | 0.164 | -497 | 84 |
|  | Household age (decades) | 56 | 136.4 | 151.8 | 0.682 | -211 | 323 |
|  | Education | 1485 | 605.5 | 907.4 | 0.014 | 298 | 2671 |
|  | Wealth axis 1 | 106 | 199.4 | 197.2 | 0.594 | -285 | 497 |
|  | Wealth axis 2 | 302 | 211.7 | 528.1 | 0.154 | -113 | 717 |
| 1 hectare clearance interaction | Established PA (Mantadia) | -6985 | 844.3 | -3061.5 | 0.000 | -8639 | -5330 |
|  | REDD+ project without safeguard | -5192 | 934.2 | -2056.9 | 0.000 | -7023 | -3361 |
|  | Established PA (Zahamena) | -3097 | 620.4 | -1847.6 | 0.000 | -4313 | -1881 |
|  | Distance from forest (km) | 792 | 165.1 | 1776.2 | 0.000 | 469 | 1116 |
|  | Household age (decades) | 295 | 148.2 | 737.0 | 0.046 | 5 | 585 |
|  | Education | 1900 | 649.0 | 1083.5 | 0.003 | 628 | 3172 |
|  | Wealth axis 1 | 140 | 215.1 | 241.5 | 0.514 | -281 | 562 |
|  | Wealth axis 2 | 430 | 230.0 | 692.5 | 0.061 | -20 | 881 |
| Strict conservation interaction | Established PA (Mantadia) | -18644 | 4410.6 | -1564.4 | 0.000 | -27289 | -9999 |
|  | REDD+ project without safeguard | -14777 | 4091.6 | -1336.6 | 0.000 | -22797 | -6758 |
|  | Established PA (Zahamena) | -3981 | 2033.2 | -724.7 | 0.050 | -7966 | 4 |
|  | Distance from forest (km) | 4034 | 983.7 | 1517.9 | 0.000 | 2106 | 5962 |
|  | Household age (decades) | 1361 | 494.4 | 1019.0 | 0.006 | 392 | 2330 |
|  | Education | 12742 | 3912.2 | 1205.4 | 0.001 | 5074 | 20410 |
|  | Wealth axis 1 | -651 | 648.5 | -371.6 | 0.315 | -1922 | 620 |
|  | Wealth axis 2 | 1049 | 640.8 | 606.0 | 0.102 | -207 | 2305 |
| Random effects | SD asc | 3472 | 431.8 | 2975.8 | 0.000 | 2626 | 4319 |
|  | SD Instalment 10 | 1692 | 542.6 | 1154.2 | 0.002 | 629 | 2756 |
|  | SD Instalment 20 | 1961 | 503.9 | 1440.3 | 0.000 | 973 | 2949 |
|  | SD Rice project | 1590 | 418.3 | 1406.7 | 0.000 | 770 | 2410 |
|  | SD 1 hectare clearance | 4767 | 385.3 | 4579.6 | 0.000 | 4012 | 5522 |
|  | SD strict conservation | 14581 | 3004.4 | 1796.2 | 0.000 | 8692 | 20470 |
