# Supplementary material for: Who bears the cost of forest conservation?
Source: PeerJ. 2018 Jul 5;6:e5106. doi: 10.7717/peerj.5106 (PMC6035863; doi:10.7717/peerj.5106)
Supplement: Supplemental Information 9 [file peerj-06-5106-s009.docx]

**S3: The annualised opportunity costs per household (USD)**

| Discount rate | minimum | median | maximum |
| --- | --- | --- | --- |
| 0.001% | 0 | 24 | 340 |
| 2.5% | 0 | 46 | 660 |
| 5% | 0 | 75 | 1077 |
