# Supplementary material for: Who bears the cost of forest conservation?
Source: PeerJ. 2018 Jul 5;6:e5106. doi: 10.7717/peerj.5106 (PMC6035863; doi:10.7717/peerj.5106)

Supplementary materials: Who bears the cost of forest conservation? Survey instruments for phase one of data collection: the household survey and the choice experiment

We present the survey instruments used (in English as well as Malagasy)

Phase 1: Household survey (English followed by Malagasy)

With Choice Experiment* (English followed by Malagasy)

*For the paper Who bears the cost of forest conservation? Only the WTA formulation is relevant as only households where this formulation was used are included in this paper (both formulations were initially tested-see Rakotonarivo 2016 for details).

Household survey in English followed by Malagasy

can paying 4 global ecosystem services reduce poverty?

les paiements pour les services écosystèmiques globaux peuvent-ils réduire la pauvreté? www.p4ges.org

Work Package 6: Household Questionnaire Survey

Survey Information

Activity/Task	Date	Start time	End time	Person(s) Responsible	Remarks
Interview					
Checking Questionnaire					
Coding Questionnaire					
Data Entry					
Checking and Approving Data Entry					

Does the respondent/household agree to be part of the follow-up surveys? [ ] **YES**

[Note: Ask this AT THE END OF THE SURVEY and record the response here.] [ ] **NO**

A. Identification

[Note: Most of the information below can be filled prior to the household survey. Fill the name of the respondent(s), GPS location of the HH and distance of the HH from the village centre to complete this section before going to the next section. For codes such as VID, FID and so on, refer to the survey guideline, and fill accordingly.]

1. HH Name & Code	*(name)*	*(HHID)*
2. Village Name & Code	*(name)*	*(VID)*
3. Fokontany Name & Code	*(name)*	*(FID)*
4. Commune Name & Code	*(name)*	*(CID)*
5. District Name & Code	*(name)*	*(DID)*
6. Name & PID of primary respondent	*(name)*	*(PID)*
7. Name & PID of secondary respondent	*(name)*	*(PID)*
8. GPS Location of the HH	*(Lat)*	*(Lon)*
9. GPS Accuracy		
10. Distance of HH from fokontany centre	*(Minutes)*	*(Km)*

B. Information about the Respondent(s) and Household Composition

1. Information about the household head and the respondent

a. Is the main respondent also the head of the household? **[If 'YES' >> go to 'c.']**	(YES=1, NO=0)
b. What is the relation of the respondent to the HH head? **[use code from 2 below]**	(code)

Codes for relation to HH head:	*1=spouse(legally married or co-habiting); 2=son/daughter; 3=son/daughter in-law; 4=grandchild; 5=mother/father; 6=mother/father in-law; 7=brother/sister; 8=brother/sister in-law; 9=uncle/aunt; 10=nephew/neice; 11=step/foster child; 12=other family; 13=not related*

[Note: Once you have established whether the respondent is also the household head from above, ask the following questions to the respondent and record their responses in the appropriate column. When the respondent is NOT the household head, also ask the respondent about the household head and fill in the respective column (for c. to i.).]

[(Non-)response codes: -98=respondent DOES NOT KNOW; -99=respondent DOES NOT WANT TO ANSWER; -100=interviewer DID NOT ASK (specify why)]

	**Respondent IS the HH Head**	**Respondent is NOT the HH Head**
c. Was the respondent born in this village? **[If 'YES' >> go to 'g.']**	(YES=1, NO=0)	(YES=1, NO=0)
d. How long has the respondent lived in the village?	_______ years	_______ years
e. Where did the respondent come from (commune and region)?		
f. Why did the respondent move to this village?	(code)	(code)
1=study; 2=work; 3=marriage; 4=land availability; 5=other (specify)		
g. Which ethnic group does the respondent belong to?	(code)	(code)
Ethinc group: 1=Betsimisaraka; 2=Sihanaka; 3=Bezanozano; 4=Merina; 5=Betsileo; 6=Other (specify)		
h. What is the marital status of the respondent?	(code)	(code)
1=married & living together; 2=married & spouse working away; 3=widow/widower; 4=divorced; 5=never married; 6=other (specify)		
i. How long ago was this HH formed?	________ years	________ years

Remarks:

2. Starting with the head of this household, please tell us about all the household members, how old are they, their education, their main occupation and how long do they reside in the household in a year.

[(Non-)response codes: -98=respondent DOES NOT KNOW; -99=respondent DOES NOT WANT TO ANSWER; -100=interviewer DID NOT ASK (specify why)]

**Personal Identification Number (PID)**	**Relationship to HH head**	**Age**	**Sex**	**Education**	**Main Occupation**	**2nd Occupation**	**3rd Occupation**	**Residency**
	[see codes below]	[or ask year of birth and note present age]	*(0=female; 1=male)*	*[For formal schooling, ask and record the number of years completed. For others, use the code as specified: 0=illiterate; 1=literate (no formal schooling); 2=vocational education; 3=other (specify).* ***For babies, write 'N/A']***	[Ask each member of the household what their primary (main) occupation is, and then ask about their other two occupations (2nd and 3rd) - if any - in terms of their importance in time spent or income earned. Use these occupation codes: 1=Agriculture; 2=Govt. job; 3=Pvt. Job; 4=Daily wage; 5=Business/trader; 6=wild product harvester; 7=Other (specify). For children who are still in school, use code 7 and specify 'in school'. For babies write 'N/A'.]			*Does the HH member live 6 months of more in the HH? (1=YES; 0=NO)*
1	*HH Head = code 0*							
2								
3								
4								
5								
6								
7								
8								

Codes for relation to HH head: 1=spouse(legally married or co-habiting); 2=son/daughter; 3=son/daughter in-law; 4=grandchild; 5=mother/father; 6=mother/father in-law; 7=brother/sister; 8=brother/sister in-law; 9=uncle/aunt; 10=nephew/neice; 11=step/foster child; 12=other family; 13=not related

Remarks:

3. Of the household members who are not resident in this household for six months or more, please provide information about where they live, their main occupation and whether they contribute to the household.

[(Non-)response codes: -98=respondent DOES NOT KNOW; -99=respondent DOES NOT WANT TO ANSWER; -100=interviewer DID NOT ASK (specify why)]

**PID**	**Current Residence**	**Length of Stay**	**Purpose**	**Contribution**	**Amount**
	[Ask for the name of the Village, Fokontany, Commune and the District]		*(1=study; 2=work; 3=marriage; 4=farming; 5=other (specify))*	*(1=Income; 0=Expenditure)*	[of income/expenditure if available]

a. How many adult children do you have who are no longer part of your household?

b. For the adult children who are no longer part of this household, please provide details about their age, sex, place of birth, place of current residence, current occupation and their reason for the move.

[(Non-)response codes: -98=respondent DOES NOT KNOW; -99=respondent DOES NOT WANT TO ANSWER; -100=interviewer DID NOT ASK (specify why)]

**Age**	**Sex (0=female,1=male)**	**Where born? (village, fokontany, commune, district)**	**Where resident now? (village, fokontany, commune, district)**	**Current occupation**	**Reason for move**
		**[Ask for the name of the village, fokontany, Commune and the District]**	**[Ask for the name of the village, fokontany, Commune and the District]**	*(1=Agriculture; 2=Govt. job; 3=Pvt. Job; 4=Daily wage; 5=Business/trader; 6=wild product harvester; 7=Other (specify))*	*(1=study; 2=work; 3=marriage; 4=land availability; 5=other (specify))*

C. Land Ownership; Land Access; and Land Use

1. Please provide information about the land that you have access to and those you used during the last agricultural year (2013/2014) - including those you lent out or rented out during 2013/2014. How many plots did you farm? How many of those were tavy plots and where? How many were tanimbary plots? How much rice seed did you plant in each plot? How did you get access to these plots? When were these plots first cleared? ***[Interviewer should draw sketch maps if that helps interviewee provide information about the plots]***

[Note: Ask here about ALL the plots that the HH has access to and can cultivate. For the plots that were cultivated during 2013/2014 (the most recent agricultural year), ask and record the (estimated) area, either in ha (m^2) OR in quantity of seeds used with units.]

[(Non-)response codes: -98=respondent DOES NOT KNOW; -99=respondent DOES NOT WANT TO ANSWER; -100=interviewer DID NOT ASK (specify why)]

**Site**	**Plot**	**Ownership/ Access**	**Bought/Borrowed/ Rented Plots**	**Lent/Rented Out Plots**		**Plot Age**	Soil fertility	**Cultivated Size**	
**[Ask and record the locally-used name of the site/ location of the plot]**	*(1=hill tavy; 2=flat tavy; 3=tanimbary; 4=tanimboly; 5=other (specify))*	*(1=inherited; 2= rented; 3=bought; 4=borrowed; 5=cleared by the household; 6=other (specify))*	**[For bought/borrowed/ rented plots, indicate whether they are from family or others]** (1=Family; 2=Others)	[Indicate whether the plot is lent out OR rented out] (1=lent out; 2=rented out)	[For lent out/ rented out plots, indicate whether they are to family or others] (1=Family; 2=Others)	*(how long since first forest clearance)*	[Ask how fertile each plot is, and use the code to record the response: 1=unfertile; 2=fertile; 3=very fertile]	*(area cultivated during 2013/2014)* ***[ha or m^2]***	*(quantity of seeds used with units)*

2. Given the resources (labour, capital, implements) you had last year, could you have cultivated more land?

[ ] YES [ ] NO [ ] Don't Know

3. If 'YES', what were your main reasons for not cultivating more land?

[Prompt from the list if necessary and record at most THREE main reasons.]

[ ] no land for sale [ ] pests (birds, animals, insects) [ ] cannot do teviala to get new land

[ ] no land to rent [ ] weeds [ ] immigrants are using land [ ] Other (specify)

D. Wild Product Harvest

1. Do you collect any (non-agricultural) products from the wild (from forest, savoka, river) for use and/or for sale?

[ ] YES >> go to '2.' [ ] NO >> go to 'E.'

2. What are the most important products that you collect? Where do you collect them from? Do you use them yourself or sell them?

[prompt using the broad categories (eg., weaving materials, medicine, food) - ask them to say what land use class they collect from, and also the preferred types of source (species) for some common products]

a. Commonly harvested products

**Products**	Type of source (species)	**Where from (type of land)?**	**Subsistence use/Sale/both?**
	[Ask & record their preferred species for these products, except for fire-wood]	**[ask and record locally used names for different types of land here - e.g., savoka mody]**	*(1=subsistence use; 2=sale; 3=subsistence+sale; 4=other (specify))*
Firewood			
Roofing material			
Material for walls			
Timber for building			
Material for floor			
Weaving material (for mats etc)			

b. What other wild-products do you collect? How often do you collect them? Do you use them yourself or sell them?

**Products**	**Where from (type of land)?**	**Subsistence use/Sale/both?**	Frequency of collection
	**[ask and record locally used names for different types of land here - e.g., savoka mody]**	*(1=subsistence use; 2=sale; 3=subsistence+sale; 4=other (specify))*	[Ask & record how frequently they collect these products. For example, 2 times a week, once a month. Make sure to record the units, i.e., per week, per month and so on.]

E. Project Affected Persons (PAPs)

1. Did you or anyone from your household participate in the survey about protected area and forest use in 2009/2010?

[ ] YES >> go to 2. [ ] NO >> go to 3. [ ] Don't know >> go to 3. [ ] Don't remember >> go to 3.

2. If 'YES', were you or the person participating identified as a forest user?

[ ] YES [ ] NO [ ] Don't know [ ] Don't remember

3. Was your household contacted earlier this year regarding any project support (related to the CAZ protected area)?

[ ] YES [ ] NO

4. Has your household received any project support recently (Riziculture, Apiculture, Igname or Aviculture)?

[ ] YES (specify) ____________________ [ ] NO

F. Assets & Wealth Indicators

1. House. Could you please tell us more about your house(s)? How many there are? What are they made of? What kind of roof have they got? Whether they are your permanent or temporary dwelling?

	**Permanent**	**Temporary**	**[Additional space to record in case of more than one house]**	
Number of houses				
Number of storey (main house)				
Number of rooms (main house)				
Roof type (main house)				
Wall type (main house)				
Other				

2. Livestock owned. Please tell us how many of these livestock does your household currently own.

	**Current Number**
Omby	
Kisoa	
Akoho	
Gana	
Gisa	
Fish tanks	
Bee hives	
Hafa (inona)	
Hafa (inona)	
Hafa (inona)	

3. How many months did your household have enough to eat during the last farming year? __________ Months

4. What kind of light do you use in the house?

[Ask giving examples, and mark all that applies.]

Petrol lamp [ ] Candles [ ] Torch (battery) [ ] Solar/rechargable lamps [ ]

Generator [ ] Other (specify) [ ] Other (specify) [ ]

5. Do you have enough light in the house?

[ ] Always [ ] Mostly [ ] Sometimes

[ ] Rarely [ ] Never

6. Household Items. Please tell is if you have got any of these items, how old they are and how much did they cost when you purchased them.
[Note: Go through the primary list with all the households surveyed. For the secondary list, make an on-the-spot assessment on whether it is worth going through the list with the household (i.e., whether the household is likely to have any of these items) and go through the list if they are likely to have the items on the list.]
[If the respondent CANNOT give the present value but can give the 'cost at purchase' and 'age of the item', record that in the blank column on the right.]
[(Non-)response codes: -98=respondent DOES NOT KNOW; -99=respondent DOES NOT WANT TO ANSWER; -100=interviewer DID NOT ASK (specify why)]

	**Number**	**Present value**	
		**[ask & record the estimated value of the item if it were to be sold now]**	
**Primary List**			
Radio			
Bicycle			
Mobile phone			
Cassette/CD Player/Portable Radio			
Torch			
Bed (metal)			
Bed (wood)			
Mattress			
Chairs			
Cleaver			
Machete			
Spade			
Plough			
Other (specify)			
Other (specify)			
Other (specify)			
**Secondary List**			
Motorcycle/Moped			
Television			
DVD/VHS Player			
Tractor			
Chainsaw			
Sewing Machine			
Solar Panel			
Generator			
Other (specify)			
Other (specify)			
Other (specify)			

G. Social & Human Capital

1. Have you or any member of your household received any training (agriculture, health, vocational skills etc.) in the last THREE years? If you have, who provided the training, for what purpose and how many times have to participated in such trainings?

**Type of training**	**Provider**	**Purpose**	**How many?**
Fambolena			
Fiompiana			
Fahasalamana			
Tontolo iainana			
Hafa [inona]			
Hafa [inona]			
Hafa [inona]			

2. Are you or any other member of your household a member of any group or an association? If you are, please provide the details about the membership.

**Type**	**Membership type**	**Membership fee**	
	(1=general member; 2=decision-making member (e.g., president, vice-pres, secretary); 3=other (specify))	*Is there a membership fee in the association? (1=YES; 0=NO)*	*If 'YES', how much is the membership fee?*
COBA			
Farmers' association/group			
Women's group			
Youth group			
Church or other religious groups			
Hafa [inona]			
Hafa [inona]			
Hafa [inona]			
Hafa [inona]			

3. Social cohesion, support network, connectedness

a. If I have serious problems (losing crops/illness etc.), I get help from my extended family.
[ ] YES [ ] Possibly [ ] NO

b. If I have serious problems (losing crops/illness etc.), I get help from my neighbour/wider community.
[ ] YES [ ] Possibly [ ] NO

c. Do you have posters of projects and/or political parties on the walls in your house?
[ ] YES [ ] NO

H. Choice Experiment

INTRODUCE AND CONDUCT CHOICE EXPERIMENT AT THIS POINT IN THE HOUSEHOLD SURVEY. CONTINUE WITH THE HOUSEHOLD SURVEY SECTION F ONCE THE CHOICE EXPERIMENT HAS BEEN COMPLETED.

END OF SURVEY >> ASK IF THE RESPONDENT/HOUSEHOLD IS WILLING TO BE PART OF THE FOLLOW-UP SURVEYS AND RECORD THEIR RESPONSE ON THE FRONT PAGE

ABOUT THE SURVEY

[Note: This is to be filled by the survey administrator/enumerator after the survey above has been completed. This is NOT to be asked to the respondents.]

1. How would you score your respondent for the following aspects in relation to the interview that you have just completed?

a. Truthfulness **[ ]**

[truthful while responding to the questions]

5=Very truthful	4=Mostly truthful	3=Partly truthful	2=Not very truthful	1=Not truthful at all

b. Openness **[ ]**

[openness in interaction with the interviewer and in responding to the questions]

5=Very open	4=Mostly open	3=Partly open	2=Not very open	1=Not open at all

c. Confusion **[ ]**

[seemed confused about the questions asked]

5=Not confused at all	4=Not much confused	3=Partly confused	2=Mostly confused	1=Very confused

2. If the respondent/household was given a gift, note what gift was given.

[ ] Cup	[ ] Salt	[ ] Sugar	[ ] Lighter
[ ] Torchlight & batteries	[ ] Oil	[ ] Candy	

3. If there are any further comments and/or important notes about this particular household/survey, please write them in the box below.

can paying 4 global ecosystem services reduce poverty?

les paiements pour les services écosystèmiques globaux peuvent-ils réduire la pauvreté?

Work Package 6: Fanadihadiana isan-tokantrano

Mahakasika ny fisy fanadihadiana

Asa	Daty	ora nanombohana	ora namaranana	tompon'andraikitra	fanamarihana
Fanadihadiana					
Famenoana ny fisy					
Marika ny fisy					
Fampidirana ny vokatry ny fanadihadiana					
Fanamarinana fa voadika ny fisy					

Manaiky ve ilay olona hadihadiana/tokantrano hanoy ny fanadihadiana mahakasika ny fambolena? [ ] **ENY**

[Fanamarihana: Anontanio amin'ny faran'ny fanadihadiana ary mariho eto ny valiny] [ ] **TSIA**

A. Famaritana tokantrano sy toerana fonenany

[Fanamarihana: Fenoy mialoha ireto alohan'ny hanombohan'ny fandihadiana fototra:Anaran'ny olona hadiahadiana, toeram-ponenany @ GPS, halaviran'ny trano miainga avy eo amin'ny ivon'ny fokontany. Ho an'ireo marika samihafa toy ny VID, FID sns,dia jereo ny torolalana mahakasika ny fanadihadihana.]

Anarany TK & Marika & Isam-pianakavy	*(Anarana)*	_______ *(HHID)*
Anaran'ny vohitra & Marika	*(Anarana)*	*(VID)*
Anaran'ny Fokontany & Marika	Ampahitra *(Anarana)*	314030011 *(FID)*
Anaran'ny kaominina & Marika	Ambohibary *(Anarana)*	314030 *(CID)*
Anaran'ny Distrika & Marika	Moramanga *(Anarana)*	MDG33314 *(DID)*
Anaran'ny olona hadihadiana voalohany & Marika	*(Anarana)*	*(PID)*
Anaran'ny olona hadihadiana faharoa & Marika	*(Anarana)*	*(PID)*
Toeram-ponenany hadihadiana GPS	*(Lat)*	*(Lon)*
GPS Fahamarinany (Precision)		
Alavirany ny Tanana (miala ny fokontany)	*(Minitra)*	*(Km)*

Rehefa vita ny fanadihadiana dia asorina ity pejy voalohany ity ary atokana @ toerana hafa. Izany dia entina manamarina fa voahaja sy voaaro ny mombamomban'ny ity tokantrano iray ity

Work Package 6: fanadihadiana isan-tokantrano FID: ________ HHID: ________

B. Famaritana ny toetry ny tokantrano

1. Mahakasika ny loham-pianakaviana na ny olona hadihadiana

a. Loham-pianakaviana ihany ve ilay olona hadihadiana? **[Raha 'ENY' >> jereo ny 'c.']**	(ENY=1, TSIA=0)
b. Inona ny fifandraisanao amin'ny loham-pianakaviana? **[ampiasao ny marika eo amin'ny fanontaniana faha-2]**	(Marika)

[Fanamarihana: : Raha ilay olona anontaniana ihany no loham-pianakaviana dia anontanio ireto fanontaniana manaraka ireto ary raiso ao aminy faritra mifanaraka amin'izany. Raha "tsy loham-pianakaviana" dia anontanio ihany ny momba ny loham-pianakaviana sy mombamomba ilay olona hadihadiana manomboka eo @ fanontaniana "c ka hatr@ i".]

	Raha "loham-pianakaviana"	Raha "tsy loham-pianakavina"
c. Teraka teto ve ianao ? **[Raha'ENY' >> jereo 'g.']**		
d. Firy taona izay ianao no nonina teto ianao?	________ taona	________ taona
e. Avy aiza toerana niavianao (Vohitra, Fokontany ary Kaominina)?	(Anarana)	(Anarana)
f.Inona ny antony nifindranao teto amin'ity Fokontany ity?	(Marika)	(Marika)
1=fianarana; 2=asa; 3=fanambadiana; 4=fisian'ny tany afaka volena; 5=hafa (inona)		
g. Avy amin'ny foko inona ianao?	(Marika)	(Marika)
Foko: 1=Betsimisaraka; 2=Sihanaka; 3=Bezanozano; 4=Merina; 5=Betsileo; 6=Hafa (inona)		
h. Manambady ve ianao?	(Marika)	(Marika)
1=miara monina; 2=tsy miaraka mipetraka; 3=maty; 4=nisaraka; 5=mpitovo; 6=hafa (inona)		
i. Firy taona no niorenan'ny tokatranonareo?	________ taona	

Fanamarihana	

2. Atombohy amin'ny loham-pianakaviana ireto fanontaniana ireto, ary hanontanio ny momba ny ankohonany : Firy taona avy ianareo. Nianatra ve sa tsia? Raha "eny" kilasy fahafiry no nijanona? Raha tsia, afaka mamaky teny sy manoratra ve? kilasy faha firy no niala niantra ianaoreo ary hafiriana no ijanonany tsirairay ato an-tokantrano mandritrin'ny taona iray.

[(Marika hoan'ireo valin-teny "tsia": -98=tsy fantany hadihadiana ny valiny; -99=tsy te-hamaly ny olona hadihadiana ; -100=tsy napetrakin'ny mpanadihady ny fanontaniana (hamarino ny antony)]

Marika fama-tarana ny olona ao an-tokantrano (PID)	Fifandraisany @ loham-pianakaviana	Taona	Sokajy	Taona nianarana	Asa fototra	Asa 2	Asa 3	fotoana hi-janonana ao an-tokantrano*
	(jereo Marika)	*(anontanio ny taona naterahany na ny taonany ankehitriny)*	*(0=Vavy; 1=Lahy)*	*(Kilasy nijanonana niantra,)(0=tsy nianatra;1=tsy nianatra fa mahay mamaky teny sy manoratra,ho an'ny ambiny raiso ny kilasy nijanonany). Ho an'ny zaza dia mariho hoe: N/A*	*[**Eto dia ezahana fantarina ireo karazana asa mety handaniany foto-ana sy hampidiram-bola ao an-tokantrano, alaharo araka ny habetsaky ny fotoana laniny,** Ampiasao ny marika eo @ fanontaniana 1=Mpamboly; 2=Mpiasa mpanjakana; 3=Mpiasa tsy miankina; 4=saran-katsaha; 5=Mpanangom-bokatra; 6= mpaka vokatra ara-boajanahary , 7= Hafa (inona, ho an'ny ankizy mbola mianatra mariho hoe " in school"]*			*Ireo olona ao an-tokantrano ireo ve mipetraka mi-hoatra ny enim-bolana ao? (1=ENY; 0=TSIA)*
1	*loham-pianakavina = Marika 0*							
2								
3								
4								
5								
6								
7								
8								
9								
10								
11								
12								
13								
14								
15								
16								
17								
18								
19								
20								
21								
22								

Marika famatarana ny fifandraisany olona ao an-tokantrano amin'ny loham-pianakaviana:	*1=vady(ofisaly na Tsia); 2=zanaka; 3=zanam-bady; 4=zafikely; 5=RAR; 6=Rafozana; 7=Rahalahy/anambavy; 8=zaodahy/ zaobavy; 9=dadatao/nenintoa; 10=zanaka anambavy/anadahy; 11=zaza natsagana; 12=havana hafa; 13=Tsisy fifandraisana*

Fanamarihana

3. Ho an'ny ireo olona tsy mipetraka maharitra ao an-tokantrano enim-bolana na mihoatra, dia anontanio ny mahakasika : ny toerana tena fonenany, ny asa fototra ataony, ny fandraisany anjara ao an-tokantrano na ny fandaniana atao aminy

[(Marika hoan'ireo valin-teny "tsia": -98=tsy fantany hadihadiana ny valiny; -99=tsy te-hamaly ny olona hadihadiana ; -100=tsy napetrakin'ny mpanadihady ny fanontaniana (hamarino ny antony)]

**PID**	**Toeram-ponenany maharitra**	**fotoana ijanona-ny any**	**Antony**	**Sandany**	**lela vola na hafa (inona)**
	(anontanio anaran'ny Fokontany, kaominina sy distrika)		*(1=Mianatra; 2=miasa; 3=manambady; 4=mamboly, 5=hafa (inona))*	*(1=mampidim-bola; 0=hamoam-bola)*	*(vola miditra/mivoka raha misy)*

4. Mahakasika ireo zanaka efa nitokantrano

a. Firy ireo zanakao efa mahaleo tena na efa mitokantrano? *(Isa)*

b.Omeo ny mombamoban'ireo zanakao efa mahaleo tena na efa mitokantrano

[(Marika hoan'ireo valin-teny "tsia": -98=tsy fantany hadihadiana ny valiny; -99=tsy te-hamaly ny olona hadihadiana ; -100=tsy napetrakin'ny mpanadihady ny fanontaniana (hamarino ny antony)]

**Taona**	**Sokajy (0=Vavy,1=Lahy)**	**Taiza no teraka? (Kaominina, Faritra)**	**Aiza ny toerana honen-any? (Kaominina, Faritra)**	**asa fototra**	**Antony fifindrana**
				1=Mpamboly; 2=Mpiasa mpanjakana; 3=Mpiasa tsy miankina; 4=saran-katsaha; 5=Mpanangom-bokatra; 6= mpaka vokatra ara-boajanahary , 7= Hafa (inona)]	*(1=Mianatra; 2=miasa; 3=manambady; 4=mamboly, 5=hafa (inona))*

C.Mahakasika ny tany hajariana

1.Afaka resahanao ve ny mahakasika ny tany nampiasainao nandritran'ny taom-pambolena 2013/2014. Firy ny tany nambolenao? Firy amin'ireo ny tavy ary aiza avy? Firy ny tanimbary? Firy ny habehan'ny tany voavoly (velarany na masomboly nampiasaina)? Ahoana ny fomba nahazoanareo ireo tany ireo? Oviana no notevesina voalohany? (Ataovy kisarisary mba hanamora ny fahazoana azy)

[(Marika hoan'ireo valin-teny "tsia": -98=tsy fantany hadihadiana ny valiny; -99=tsy te-hamaly ny olona hadihadiana ; -100=tsy napetrakin'ny mpanadihady ny fanontaniana (hamarino ny antony)]

**Toerana**	**Tany**	**Fomba nahazo-ana ny tany**	**tany novidiana/ nindramina/ nofaina**	**Tany nam-pindramina/ nampanofaina**	**Tany nampindramina/ nampanofaina**	**taona nanajariana ny tany**	**Toetrin'ny tany**	**Velaran'ny tany nambolena**	
**[Raiso eto ny anaran'ny fiat-son'ny olona eny an -toerana ny misy tany hajariana]**	*(1=tavirana tavy ; 2=lemaka tavy; 3=tanimbary; 4=tanimboly; 5=hafa (inona))*	*(1=lova; 2= nofaina; 3=novidiana; 4=nindramina; 5=ny tokantrano no nitevy voalohany; 6=hafa) (inona)*	**[Mariho eto raha novidiana, nin-dramina na nofaina/ t@ fian-akaviana na olona hafa]** (1=fianankaviana; 2=olona hafa)	1=nampindramina 2=nampanofaina	**[Mariho eto raha novidiana, nindramina na nofaina/ t@ fian-akaviana na olona hafa]** (1=fianankaviana; 2=olona hafa)	*(hafiriana no nana-jariana ny tany voalohany)*	*(Inona no karazan'ny tany? 1=malemy 2=masinamasina 3= masi-na)*	*(velaran-tany voavoly t@ taom-pambolena 2013/2014)* ***[ha or m²]***	*(masom-boly voavoly): mariho unites:* ***kg, kpk, vata***
Fanamarihana									

2.Raha ny enti-manana eo ampelatananao izao sy ny sandry miasa ato an-tokantranonao, mety hoe mbola nahavita tany nihoatra noho ny vitanao tamin'ity taona ity ve ianao?

[ ] Eny [ ] Tsia [ ] Tsy hay

3. Raha 'Eny' dia inona ny antony tsy nahafahanao nanao izany?

[ ] tsy misy tany azo vidiana [ ]tsy misy tany afaka hofaina [ ] tsy afaka manao teviala mba hahazoana tany vaovao [ ] ahi-dratsy

[ ] lasan'ny mpiavy daholo ny tany [ ] biby manimba voly (bibikely,vorona,) [ ] Hafa (inona)

Ampiasao ity pejy ity hoan'ny fandraisana ireo toerana misy ny tany hajariany tokontrana na voavoly na savoka.

D. Vokatra anaty ala

1. Amin'ny fiainanareo andavan'andro, mampiasa ireo karazana vokatra ara-boajanahary hita eny rano, ala, savoka?

[ ]Eny >> jereo '2.' [ ]Tsia >> jereo 'E.'

2. Inona ny vokatra tena alainareo amin'izany ? Aiza no maka azy ireo? Ampiasainao amin'ny filànao andavan'andro ve sa amidy?

[atambaro isakin'ny sokajy ireo vokatra alaina (eg., fanafody, sakafo, fitaovana,) - anontanio ny toetran'ny toerana hangalana izany]

a. Inona avy ireo akora tena ampiasainareo @ fanaboarana trano. Ampiasainareo @ filanareo ihany ve sa misy amidy ihany koa?

**Vokatra**	**Akora**	**Aiza no maka azy: ala/savoka/tany kinina/kesika hafa(inona))?**	**Filàna ao an-tokantrano/amidy/izy roa?**
	[anontanio ny karazana akora ampiasaina @ fanamboaran-trano]	[hanontanio ary raiso ny fiantsoana ny toerana fangalana ny vokatra]	(1=filàna ao an-tokantrano; 2=amidy; 3= sady ampiasaina hoan'ny filàna ao an-tokantrano no amidy; 4=hafa (inona))
Kitay			
Tafo			
Rindrina			
Taolona trano			
Gorodona			
Rary			

b. Inona avy ireo vokatra hafa ara-boajanahary alainareo. Manao ahoana ny fampiasana azy?

**Vokatra**	**Aiza no maka azy: ala/savoka/tany (Kinina/kesika) hafa (inona))?**	**Filàna ao an-tokantrano/amidy/izy roa?**	**impiry isan-taona @ ankapobeany no maka ireo vokatra ireo?**
	[hanontanio ary raiso ny fiantsoana ny toerana fangalana ny vokatra]	(1=filàna ao an-tokantrano; 2=amidy; 3= sady ampiasaina hoan'ny filàna ao an-tokantrano no amidy; 4=hafa (inona))	[Mariho na isan-kerinandro na isam-bolana na hafa]

E. Olona voakasikin'ny tetik'asa (OVT)

1. Ianao ve na olona tatao an-tokantrano efa nandray anjara t@ fanadihadiana mahakasika @ fampiasana ny ala arovana t@ 2009/2010 ?

[ ] ENY >>jereo '2' [ ] TSIA >>jereo '3' [ ] Tsy fatatro >>jereo '3' [ ] tsy tadidiko >>jereo '3'

2. Raha "ENY", ianao ve na olona tato an-tokantrano izay nandray anjara t@ izany fanadihadiana izany dia voa marika fa olona mampiasa ny ala ?

[ ] ENY [ ] TSIA [ ] Tsy fatatro [ ] tsy tadidiko

3. Ianao na ilay olona ato an-tokantrano izay voa marika fa mpampiasa ny ala ve dia nantsoina vo aingana t@ ity taona ity fa anisan'ireo mahazo tetik'asa @ taona ity izay mifandraika indrindra @ fiarovana aty ala CAZ?

[ ] ENY [ ] TSIA [ ] Tsy fatatro [ ] tsy tadidiko

4. Ny tokantranonareo ve efa nahazo ilay tetik'asa (voly vary, fiompina tantely, voly ovy ala na fiompina akoho)?

[ ] ENY [Inona] ______________________________ [ ] TSIA

F. Fananana

1. Trano

	**Maharitra**	**Mandalo**	**[Hafa raha mihoatra ny iray]**	
Isan'ny trano				
Isan'ny rihana (trano maharitra)				
Isan'ny efitra (trano maharitra)				
Karazan'ny tafo (trano maharitra)				
Karazan'ny rindrina(trano maharitra)				
Hafa				

2. Fiompiana ***[Firy ny isan'ny biby fiompy anananareo ao an-tokantrano?)***

	**Isany**
Omby	
Kisoa	
Akoho	
Gana	
Gisa	
Hafa [inona]	
Hafa [inona]	
Hafa [inona]	

3. Firy volana no tena hitanareo fa ampy ny sakafonareo tamin'ny taom-pambolena 2013/2014?

4. Inona ny jiro ampiasainareo?

[Anontanio dia mariho raha ampiasainy ary jerijereo ao an-trano]

jiro petrola [ ] labozia[ ] lampy (piles) [ ]

elektrika [ ] Hafa inona [ ] Hafa inona [ ]

5. Ampy ve ny jironareo ao an-trano?

[ ] Ampy foana [ ] Matetika [ ] indraindray

[ ] Mahalana [ ] tsy ampy

6. Fitaovana ao an-tokantrano. Raha misy amin'ireto fitaovana ireto ao an-tokantranonareo dia firy taona no nanana azy ary ohatrinona ny vidiny tamin'izany?

[Fanamarihana: Anontanio aloha ireo zavatra hita eo amin'ny lisitra voalohany, raha toa ka hitanao fa ilaina ny miapita ao amin'ny lisitra faharoa dia tohizo anontaniana ireo fananana hita ao amin'io lisitra faharoa io]

[Raha tsy afaka manome ny nividina ankehitriny ny olona hadihadiana nefa afaka manome kosa ny vidiny t@ voanofidiana sy ny taonany fitaovana dia raiso eo sisiny havanana izany]

[(Marika hoan'ireo valin-teny "tsia": -98=tsy fantany hadihadiana ny valiny; -99=tsy te-hamaly ny olona hadihadiana ; -100=tsy napetrakin'ny mpanadihady ny fanontaniana (hamarino ny antony)]

	**Isa**	**vidiny amin'izao fotoana izao**	**vidiny nividiana azy**	**taonany**
		[hanontanio ary raiso ny vidiny fitaovana @ ankapobeany ankehitriny raha tsy fatany dia anontanio ny vidiny t@ nividanana azy ary ny taona nahazoana azy]		
**Lisitra voalohany**				
Radio				
Bisikileta				
Finday				
Radio Cassette/CD /carte				
Lampy				
Fandriana (hazo)				
Fandriana (vy)				
Kidoro @ tany				
seza				
**Antsy be**				
Famaky				
Angady				
angadin'omby				
Hafa(inona)				
Hafa(inona)				
Hafa(inona)				
**Lisitra faharoa**				
Moto				
Vata fahita lavitra				
DVD/VHS Player				
trakitera/kibota				
tsofa elektrika				
masinina fivelezana vary				
Fitaovana mamokatra herinaratra avy @ herin'ny masoandro				
Fitaovana mamokatra herinaratra				
Hafa(inona)				
Hafa(inona)				
Hafa(inona)				

G. Mahakasika ny Sosialy sy fifandraisana @ mpiara-monina

1. Fiofanana(Efa nanaraka fiofanana(fambolena, fahasalamana, etc.) tao anatin'ny 3 taona? Iza no nanome fiofanana ary mahakasika ny inona ?)

**Karazany**	**Mpampiofana**	**Tanjona**	**Im-piry**
Fambolena			
Fiompiana			
Fahasalamana			
Tontolo iainana			
Hafa [inona]			
Hafa [inona]			
Hafa [inona]			

2. Fikambanana

**Karazany**	**Anjara asa**		**Latsa-kemboka**
	**(1=mpikambana tsotra; 2=mpitantana fikambana (e.g., prezida, vice-pres, mpitantsoratra); 3=hafa (inona))**	**Misy latsa-kemboka aloha ve ao @ fikambanana? (1=ENY; 0=TSIA)**	**Raha'ENY', ohatrinona izany latsa-kemboka izany?**
VOI			
Fikambanan'ny tantsaha			
Fikambanan'ny vehivavy			
Fikambanan'ny tanora			
Fikambanana ara-pinoana			
Hafa [inona]			
Hafa [inona]			
Hafa [inona]			
Hafa [inona]			

3. Fifandraisana amin'ny mpiara-monina sy ny fianakaviana

a. Raha sendra olana goavana ianao toy ny aretina, fahaverazan'ny vokatra sns dia mety ahazo fanampiana avy amin'ireo havanao ve?

[ ] Eny [ ] Angamba [ ] Tsia

b. Raha sendra olana goavana toy ny aretina, fahaverazan'ny vokatra sns dia mety ahazo fanampiana avy amin'ireo mpiara-monina na ny olona manodidina?
[ ] Eny [ ] Angamba [ ] Tsia

c. Misy paositera avy amin'ny tetik'asa na avy amin'ny vondrona ara-politika ve ao @nareo?

[ ] ENY [ ] TSIA

H. Choice Experiment

Antobohy eto ny choice experiment.

MAHAKASIKA NY FANADIHADIANA

[Fanamarihana: Ity dia natokana ho an'izay nanao ny fanadihadiana]

1. Ahoana ny fahitanao ny fanadihadiana araka ireto mari-drefy ireto?

**a. Fahatokisana ny valin-teny nomeny**	**[ ]**			
5=Tena azo antoka tanteraka	4=Azo antoka	3=Azo antoka amin'ny ampahany	2= tsy dia azo antoka firy	1=tsy misy azo antoka mihintsy
**b. Fisokafany miresaka**	**[ ]**			
5=Tena niresaka tsara	4=Niresaka tsara	3=Niresaka ihany	2=tsy dia niresaka firy	1=tsy niresaka tsara
**c. Fahasahiranana maneho hevitra(na mandray hevitra)**	**[ ]**			
5=tsy sahirana mihintsy	4=tsy sahirana	3=tsy sahirana loatra	2=somary sahirana	1=tena sahirana be

2. mariho izay fanomezana nomena ilay tokantrano.

[ ]Kopy	[ ] Menaka	[ ] sira	[ ]briquet	[ ] vatomamy
[ ] lampy + piles	[ ] siramamy			

3. Raha misy fanamariahan hafa dia soraty eto izany.

Scripts for the implementation of the choice experiment (in English followed by Malagasy)

H. Choice experiment

[Summary English translation of the Malagasy choice experiment survey section (supplement to section H. in the household survey questionnaire)]

WILLINGNESS TO ACCEPT QUESTIONNAIRE

General introduction, consent and anonymity

"We are university students and researchers who aim to better understand your livelihoods. We would like to do a valuation exercise that we framed as a game in which you will be asked to consider different choice scenarios that you are well knowledgeable about. We will ask you to choose what you prefer most among the alternatives i.e. which option you think is best for your livelihoods / welfare. Please kindly note that even if we frame it as a game we ask you to answer as you would in a real situation.

The results of the research can be used by policy makers. But please note that we are conducting an independent research, i.e. we are independent of the government and thus we have neutral views with regard to forest use and forest policies. We also reassure you that your answers will not be communicated to anyone in a form where your reply can be linked to you.

First of all, we would like to kindly ask your consent, i.e. whether you willingly accept to be interviewed. Know that if you are happy to be interviewed, you can still stop us at any time or refrain from answering any question that you are not comfortable with.

1. Introduce the technical and material support for improved rice farming

Please consider a major foreign donor who would like to provide you with some development assistance (we used a doll to represent the donor). The donor would like to help you specifically with the improved rice cultivation technique. It targets rice cultivation both on hills and flat lands and it can be described as a sustainable and modern agricultural package that includes productivity enhancing practices such as the use of fertilisers, insecticides and/or herbicides. It involves digging and possibly the construction of terraces for slopes and precludes the use of fire as a way to maintain fertility while not fallowing the land. Each household will also be provided with some agricultural materials and inputs such as improved seeds, fertilisers, wheelbarrows, spades, etc.

Question: How familiar are you with such improved rice cultivation technique? (coding: 3= I've done it myself, 2=I've seen other people doing it, 1= I've heard about it, 0=never heard nor seen it)

If you were receiving tools for improved rice cultivation for free, how promising that would be for improving your livelihood? "I believe that the technical rice farming would better my livelihoods" - To be measured on a Likert scale from 1 to 5 (1='strongly disagree', 2='disagree', 3= 'neither disagree nor agree', 4='agree', 5='strongly agree')

2. Compare the improved rice cultivation with cash payments

Next consider that the donor lets you freely choose which development assistance you find best for your livelihoods. Therefore, the donor also offers to give you some cash payments that you can invest in any alternative income generating activities of your choice or to purchase materials or fertilizers, etc. Such cash payment would be managed by an independent external institution such as a microcredit or an access bank which will provide you with savings accounts.

Now, consider that the donor is asking you to choose between the two following options.

A	B
**You will be donated** 1,000,000 MGA cash	You will be provided with support for the improved rice cultivation

If the respondent chooses the cash payment, ask him/her, what would s/he do with the money?

In what follows, we will ask other scenarios but you have to look at each independently from each other.

3. Introduce *teviala* on one ha of forestland

Now, please consider that the government would make it possible for you to get a permit to do *teviala* on one hectare of forestland. This would be like a new additional land, still very fertile for which you have a legal title (no risk of being penalized by the government).

What would you cultivate on that land if you were offered it? For how many years would you cultivate there before fallowing the land?

Now, please consider the two following choices and choose which one you like most:

A	B
**You will be donated** 6,000,000 MGA (6 tapitrisa) cash	You will be provided with support for the improved rice cultivation
You would receive that cash in 10 instalments (i.e. 600,000 per year for 10 years)	-
You must not do teviala anymore (forever)	You will be offered one *teviala* permit on one ha

Please know that the *teviala* permit on one hectare is a one-off opportunity. Likewise, the rice cultivation technique would be a one-off project.

4. Introduce free *teviala*

Next, please consider that the government would make it possible for you to get a permit to do *teviala* on an unlimited forestland (i.e. not limited to one hectare). This would be similar to the former President Ratsiraka's government (1975-1991) where *teviala* permits were formally granted to rural farmers. That would mean that the government would stop enforcing forest protection and you can think of it as an open forest frontier.

So if you were offered the choice below, which one would you choose? I.e. which one is the best option for your livelihoods?

A	B	C
Cash payment: 9,000,000 MGA	-	Free *teviala*
Number of instalment: 10 years (900,000 per year)	-	
No support for improved rice cultivation	Support for improved rice cultivation	
You and your children must not do *teviala* presently and in the future (forever)	*Teviala* permit on 1 ha	
(*need to emphasize here that they can still buy paddy field or borrow or rent lands**)		

Know that in choice A where you must not do *teviala*, protection will be strictly enforced (will need to place some officer dolls in the alternative). If you are caught doing *teviala* even in one small area of forestland, you must face at least 5 years of imprisonment. No one will escape as patrolling efforts will be very stringent.

Also, in alternative B (*we used colourful background paper to distinguish the three alternatives*), you must not exceed one hectare or do *teviala* elsewhere without a permit. The sanction would be the same if you don't abide by the rules.

5. Introduce timeframe/number of instalments

Next, please consider that in the following choice, you must not do *teviala* anymore. Which one would you choose?

A	B
**Cash payment**: 9,000,000 MGA	**Cash payment**: 6,000,000 MGA
Number of instalment: 20 years (450,000 per year)	Number of instalment: 10 years (600,000 per year)
No *teviala*	

Ask the respondent if s/he has any questions, then proceed with the first choice card of the DCE survey. The position of the reference level alternative (i.e. the open forest frontier) was alternated in the six choice tasks (across the three columns, A, B, and C).

COMPREHENSION AND CERTAINTY

Respondents' rated comprehension (scale 1 to 5) – For the overall CE exercise – to be measured by the enumerator)

1: The respondent doesn't understand the CE valuation exercise at all, all the choices seem inconsistent, barely finished the choice cards

2: Many inconsistencies but at least finished all the choice cards

3: So so, the respondent seems to understand the generalities but still missed the details, still made few inconsistent choices

4: In general, good understanding of the details and generalities, made only one- two inconsistent choices

5: Perfect, understands very well everything

Certainty codes (to be scored by the interviewers for each choice card on a scale of 1 to 5)

1: very uncertain, changed his/her choice many times, asked the interviewers to re-explain the choice cards again one or two more time, took ages to finally make up his/her mind

2: uncertain, the respondent took also very long but least a little bit quicker than 1

3: so, so, seems a bit uncertain but at least when s/he made his choices eventually, s/he seemed certain.

4: certain of his/her choice, the respondent was quick and didn't request any additional explanations

5: no doubt at all, relatively quick, made up his mind after the first round of explanation.

Follow-up questions

[Please see 'Follow-Up Questions' in the Malagasy version. An English summary is provided after each question]

WILLINGNESS TO PAY QUESTIONNAIRE

Introduction, consent and anonymity

We are university students and researchers who aim to better understand about your livelihoods. We would like to do a valuation exercise related to your livelihoods. The exercise will be framed as a game in which you will be asked to consider different choice scenarios that you are well knowledgeable about. We will ask you to choose what you prefer most among the alternatives i.e. which option you think would be best for your livelihoods / welfare.
Please know that we are conducting an independent research, i.e. we are independent of the government and thus we have neutral views with regard to forest use and forest policies.

First of all, we would like to kindly ask your consent, i.e. whether you willingly accept to be interviewed. Know that if you are happy to be interviewed, you can still stop us at any time or refrain from answering any question that you are not comfortable with.

Be reassured that your answers will not be communicated to anyone in a form where your reply can be linked to you. Consequently your answers cannot be used against you, and we ask you to truthfully reveal your most preferred alternative on the basis of what is your best livelihood option.

1. **Introduce the improved rice project**

Please consider that you will be given the opportunity to buy a household project which can provide you with alternative livelihood strategies; i.e. a project that you can use to complement your income generating activities, such project will all include technical support from the start till the end production and start up materials. It targets rice cultivation both on hills and flat lands and it can be described as a sustainable and modern agricultural package that includes productivity enhancing practices such as the use of fertilisers, insecticides and/or herbicides. It involves digging and possibly the construction of terraces for slopes and precludes the use of fire as a way to maintain fertility while not fallowing the land. Each household will also be provided with some agricultural materials and inputs such as improved seeds, fertilisers, wheelbarrows, spades, etc.

Please consider that the primary project which you could purchase now is the improved rice cultivation technique. It specifically targets rice cultivation on steep hills and its main objective is to maintain soil fertility.

Question: How familiar are you with such improved rice cultivation technique? (coding: 3= I've done it myself, 2=I've seen other people doing it, 1= I've heard about it, 0=never heard nor seen it)

In your opinion, how promising is such improved rice cultivation with regard to improving your livelihoods? "I believe that the technical rice farming would better my livelihoods" - To be measured on a Likert scale from 1 to 5 (1='strongly disagree', 2='disagree', 3= 'neither disagree nor agree', 4='agree', 5='strongly agree')

2. Introduce teviala permit on one ha of forestland

Now, please consider that you would also be able to buy a permit to do *teviala* on one hectare of forestland. This would be like a new additional land, still very fertile for which you have a legal title (no risk of being penalized by the government).

What would you cultivate on that land if you were offered it? For how many years would you cultivate there before fallowing the land?

Now, if you could afford both alternatives below, which one would choose?

A	B
A *teviala* permit on one ha	Improved rice cultivation project

Please know that the *teviala* permit on one hectare is a one-off opportunity, i.e. your household would be given the chance to buy it only once in your lifetime, likewise, the rice cultivation technique would be a one-off project.

Next, which one would you choose in the following scenario:

A	B
**You pay**: 500,000 Ar in 10 instalments (50,000 per year)<br>You get a *teviala* permit on one ha	**You pay**: 100,000 Ar in 10 instalments (10,000 per year)<br>You get the improved rice cultivation project

Note that you can pay only after harvest. But please we would like to kindly remind you to carefully consider whether you would be really able to afford the one you choose.
Know that you would be paying the government through state agents, and the permit would be legal. Note that the *Fokontany* and independent stakeholders would also be involved to ensure transparency.

3. Introduce free *teviala* and number of instalments

Next, please consider that the government would make it possible for you to buy a permit to do *teviala* on an unlimited forestland (i.e. not limited to one hectare). This would be similar to the former President Ratsiraka's government (1975-1991) where *teviala* permits were formally granted to rural farmers. That would mean that the government would stop enforcing forest protection and you can think of it as an open forest frontier.

So if you were offered the choice below, which one would you choose? I.e. which one is the best option for your livelihoods?

A	B
Cash payment (you pay in total) 1,500,000 MGA<br>Number of instalment: 20 years (75,000 per year)<br>Free *teviala*	Cash payment (you pay in total) 1,000,000 MGA<br>Number of instalment: 10 years (100,000 per year)

4. **Introduce an example of choice card**

Next, which of the alternatives below would you choose? Please note that you need to carefully think of your budget constraints i.e. consider whether you would be really able to afford the one you choose.

**A**	**B**	**C**
You pay in total: 3,000,000 MGA	You pay in total: 500,000 MGA	No payment and no *teviala* (forest protection strictly enforced) – (*this must not sound too negative, will need to emphasize here that they can still buy paddy field or borrow or rent lands*)
Number of instalment: 20 years (150,000 per year)	One instalment	
No project	You get the improved rice cultivation project	
Free *teviala*	You get one *teviala* permit (1ha)	

Know that in choice C where you must not do *teviala*, protection will be strictly enforced (will need to place some officer dolls in the alternative). If you are caught doing *teviala* even in one small area of forestland, you must face at least 5 years of imprisonment. No one will escape as patrolling efforts will be very stringent.

Also, in alternative B (this will be referred to as the colour of the background paper), you must not exceed one hectare or do *teviala* elsewhere without a permit. The sanction would be the same if you don't abide by the rules.

Ask the respondent if s/he has any question, then proceed with the first choice card

COMPREHENSION AND CERTAINTY

Comprehension codes (scale 1 to 5) – For the overall CE exercise – to be measured by the interviewer)

1: The respondent doesn't understand the CE valuation exercise at all, all the choices seem inconsistent, barely finished the choice cards

2: Many inconsistencies but at least finished all the choice cards

3: so so, the respondent seems to understand the generalities but still missed the details, still made few inconsistent choices

4: In general, good understanding of the details and generalities, made only one- two inconsistent choices

5: Perfect, understands very well everything

Certainty codes (to be scored by the interviewers for each choice card on a scale of 1 to 5)

1: very uncertain, changed his/her choice many times, asked the interviewers to re-explain the choice cards again one or two more time, took ages to finally make up his/her mind

2: uncertain, the respondent took also very long but least a little bit quicker than 1

3: so, so, seems a bit uncertain but at least when s/he made his choices eventually, s/he seemed certain.

4: certain of his/her choice, the respondent was quick and didn't request any additional explanations

5: no doubt at all, relatively quick, made up his mind after the first round of explanation.

FOLLOW-UP QUESTIONS

[Please see 'Follow-Up Questions' in the Malagasy version. An English summary is provided after each question]

CHOICE EXPERIMENT SURVEY IN PRACTICE

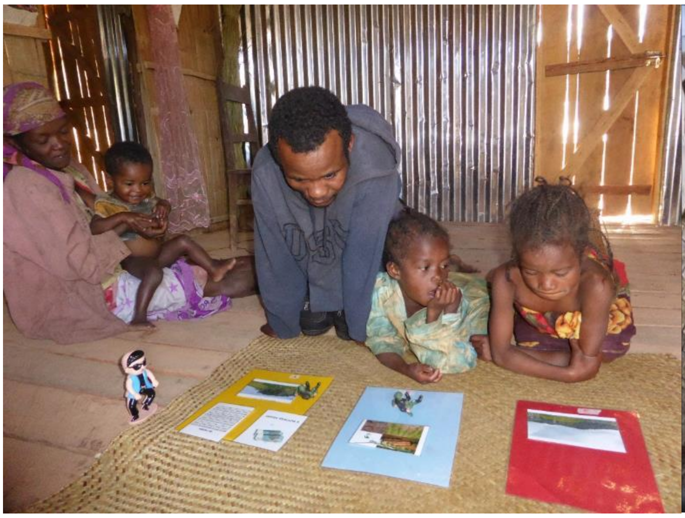

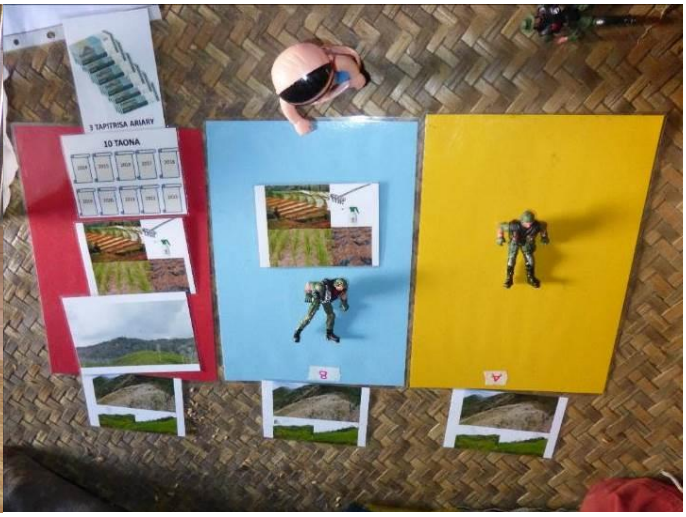

H. CHOICE EXPERIMENT

[Original Malagasy-language choice experiment survey section (supplement to section H. in the household survey questionnaire)]

WILLINGNESS TO ACCEPT QUESTIONNAIRE

WTA (izay en italique sy voatsipika dia fanazavana ho antsika interviewers fa tsy mila resahina ilay olona adihadiana)

Introduction (*teboka telo voalohany atao mialoha ny HH QUESTIONNAIRE*):

FREE AND PRIOR INFORMED CONSENT (FPIC)

Izahay dia mpianatra mpikaroka avy amin'ny Oniversiten'Antananarivo. Ny fianarana izay ataonay dia mikasika ny velontenantsika aty ambanivolo. Mety aharitra ora iray eo ny koranantsika. Ary hizara sokajy roa: Voalohany dia fanontaniana vitsivitsy mahakasika ny famelomantenan-dreo: fambolena, fiompiana, sy fivelomana hafa ataondreo itondrana vady aman-janaka, ary ny faharoa dia hanano karazana kilalaon-tsaina izay isika na an-kitsapaka, mbola mahakasika velon-tena ihany ary ho azavaiko misimisy rehefa avy eo. Ity pejy misy sary izay apetrakay aminareo ity moa dia manazava amin'ny antsipirihany hoe zovy moa izahay ary inona tsara ilay fikarohana ataonay (*project leaflet*). Aza manahy fa raha sendra tsy mahay mamaky teny moa isika ato antrano dia mbola ho azavainay misy2 miandalana eto ihany ny ao anatiny.

Voalohany indrindra dia te ahafantatra izahay raha mazoto sy malalaka ianareo ikorana aminay. Ny resaka ifanaovantsika moa dia an-tsitrapo sy malalaka tanteraka fa tsy terena akory.

Rehefa manaiky izy:

Misaotra mialoha ny amin'ny finiavana asehonareo. Eny antenantenany eny, afaka manapaka mametraka fanontaniana ianareo na mangataka ny hijanona. Raha toa koa ka misy fanontaniana tsy tianareo ho valiana dia zonareo tsara ny mizaka hoe tsy hamaly ary dia afaka miroso amin'ny fanontaniana manaraka isika.

Mazava hatreto? Misy fanontaniana ve na fanamarihana?

REASSURE RESPONDENT ABOUT ANONYMITY

Fantaro fa fianarana / fikarohana ity ataonay ity, ary afaka mahazo toky isika fa ny valinteny omentsika eto na ny momba momba antsika dia tsy hozarainay na amin'iza na amin'iza fa mijanona ho tsambaratelontsika. Ho raketinay amin'ny fomba hafa kely ny valinteninareo (laharana fa tsy anarana) hany ka tsy misy afaka ny hahalala hoe zovy no nizaka inona. Izahay rahateo moa tsy maka anarana na hoe laharana karam-panondro izany. Afaka tena matoky isika noho izany fa tsy misy raha tokony ahavaka antsika ny korana eto ary afaka tena mamboraka izay am-pontsika isika mahakasika ny fameloman-tenantsika. Tsara ny manarimaka fa ity fanadihadiana ity sy ny fitaovana ampiasainay dia tsy aty amin'ny faritra misy anareo ihany no anaovanay azy fa efa nampiasaina tamin'ny faritra hafa teto Madagasikara hatrany ampitan-dranomasina.

WHAT IS RESEARCH? (*Optional: Tsy voatery atao ety amboalohany fa afaka atao amin'ny farany koa na rehefa mametraka fanontaniana zareo ohatra hoe, inona ny voka-tsoa azo avy amin'ilay fanadihadiana, sns.).*

Izahay dia tena mpianatra / mpikaroka mahaleo-tena tanteraka ary tsy manao asa fampandrosoana. MARIHINAY fa izahay dia tena tsy misy ifandraisainy amin'ny ANGAP (*na MNP izay ahafantarandry zareo azy*) na ny fanjakana. Izany hoe tsy miankina amin'ny Fanjakana na koa hoe iraky ny Fanjakana ary tsy miara-miasa amin'ny NGAP fa MPIANATRA MPIKAROKA ihany, Izany hoe ny andraikitray dia azo lazaina ho mizara mizara dingana roa: 1) mangadihady sy mijery ny zava-misy ianantsika ety ifotony (tena mitety tokantrano tsiraikaraiky mht na lavitra na akaiky) ary rehefa vita izany dia 2) manoratra boky mikasika ny valim-pikarohana ary manolotra soson-kevitra ny fanjakana sy ny sehatra iraisam-pirenena. Ary dia anjaran'ireo ny mandinika sy manantanteraka asa fampandrosoana. Izahay kosa tsy afaka ny ampanantena fa mandainga si izahay raha mizaka izany.

RANDOM SAMPLING OF HOUSEHOLD AT THE VILLAGE LEVEL (*Optional: afaka rehefa manontany zareo hoe ahoana ny fomba nisafidianana ny tokantrano vao azavaina)*

Ity fangadihadiana ataonay ity dia isan-tokantrano, ny atao hoe tokantrano moa dia ireo olona iray tafo, iray mahandro, iray fivelomana. Sitrakay tokoa raha ny tokantrano rehetra eto xxx no adihadianay saingy ny fotoana moa voafetra sady izahay koa efatra mianadahy si dia voatery ampahany si no vitanay. Izahay tsy hoe nifidy ireo ampahany ireo na nanavakavaka fa nataonay ankitsapaka, toy ny natao tanaty satroka ny anaram-bositra fiantsoana antsika dia izay voaray tao tokantrano adihadianay.

ETO MANOMBOKA ILAY CE:

Amin'ity dingana manaraka ity izany ity dia hiara haminavina toe-javatra maromaro isika, izany hoe hijery fanoharana izay miendrika kilalaon-tsaina na ankitsapaka isika, ireo fanoharana ireo dia mahakasika ny velontenantsika.

Na dia hoe hiresaka betsaka ny hoe Fanjakana na ANGAP isika (*na MNP izahay ahalalandry zareo ny national park*) anatin'ity kilalaon-tsaina ity dia MARIHINAY fa izahay dia tena tsy misy ifandraisainy amin'ny ANGAP na ny fanjakana fa MPIANATRA MPIKAROKA ihany, mahaleo-tena tanteraka izahay. Ilay Fanjakana na ANGAP horesahanay ato anatin'ity fanoharana ity dia ohatra ihany ampaisainay amin'ity fianarana ity, fa tsy hoe izahay akory no Fanjakana. Raha haka ohatra iray izao isika anazavainay izay hoe maha- maha mahaleo-tena anay izay dia sady tsy mandrara anareo hanano atiala ohatra izahay no tsy mandrisika anareo hanano atiala koa etsy andaniny. Izany hoe tsy miandany izahay.

Ampahatsiahivinay fa tena fianarana na fitsapa-kevitra ihany ity ataontsika manaraka ity.

PLEASE, REMIND RESPONDENT ABOUT ANONYMITY HERE (LE HOE TSY MAKA ANARANA SY TAZOMINA HO TSIAMBARATELO NY KORANA, TSY MIVOAKA NY RINDRINA EFATRA).

Koa manentana antsika aho araka izany hilaza ny safidy izay tena andrian'ny saintsika.

⇨ ***Remarque generale hafa hoa antsika interviewer:***

- *Ilay raimpianakaviana no tena mila mamaly, afaka hoe eo koa ilay vadiny manampy azy fa izy no tompokevitra farany,*
- *Mila rassurer-na be zareo eny am-boalohany hoe tsy anelingelina ny asa fivelomany akory isika fa ny fivelomany hatrizay mbola afaka ataony foana ihany fa tena hoe ivelany na fanampiana ilay efa velontena si no resahantsika anatin'ity kilalaon-tsaina ity.*
- *Isaky ny miova choice card dia preciser-o tsara foana hoe hafa indray koa ity manaraka ity, tsy misy ifandraisany amin'ny teo aloha. Izany hoe asaina ataony toy ny mbola tsy nisafidy mihitsy izy teo aloha.*
- *Angamba tsara preciser-na koa amin'ilay teviala 1 ha hoe opportunite indray mandeha monja io fa tsy hoe isan-taona akory dia afaka ividy anio foana. Dia tahak'izany koa ilay voly vary teknika, fanampiana indray mitontona fa tsy misy foana akory isan-taona.*
- *Tsara koa preciser-na raha nisafidy ilay tsy manano atiala izy hoe mandrakizay izy io ho azy sy ny taranany (izay mbola ao anaty tokantrano) tsy afaka ny hanano intsony mihitsy fa sao dia mbola manantena hoe mbola afaka ahazo an'ilay teviala 1 ha any aoriana any.*
- *Eo amin'ilay fampidirana anle voly vary teknika, mila normaliser-na ihany le izy fa tsy afaka ny hitompo teny fatratra be isika hoe tena hamokatra be io. Mety au contraire aza mantsy ho ratsy be ny vokatra*

1. Introduire le voly vary teknika:

Voalohany indrindra dia andeha isika haminavina hoe misy mpamatsy vola vahiny iray tonga ety amintsika mba hitondra asa fampandrosoana satria hita fa sahirankirana isika ety ambanivolo. Andeha ataontsika hoe te-hanampy eo amin'ny fambolem-bary io mpamatsy vola io ka hampianatra anao izany hoe voly vary teknika izany. Izy io moa dia mety aby na vary an-tanety na horaka ary ny tena tanjona amin'izy io dia hitazomana ilay tsiron-tany (mba tsy malady masina[1] ilay tany). Ao anatin'izany fanamapiana voly vary teknika izany dia misy masomboly voafantina anaovana andrana omena anao eo amin'ny 30 kapoaka (3 kopy) eo, izany masomboly voanfantina izany moa dia masomboly mety amin'ny tany aty. Ankoatran'io masomboly io dia misy koa ny zezika biolojika ary ny fanafody behatoka ilaina amin'izany ary koa ny fiofanana na fampianarana antsika izany teknika vaovao izany (izany hoe hisy mihitsy teknisiana ho avy aty aminao ampianatra anao izany teknika izany). Omena fitaovana madinidinika koa ianao toy ny angady, rateau, fourche, sns. Mazava tsara ve? _*Aseho azy le sary voly vary teknika.*_ Mety misy asa tany sy asa angady (mamadika benja) mila ataonao mialohan'ny hambolena fa izany moa dia ho ampianarina anao (_*aseho azy ilay sary ambany ankavia*_ (ecobuage).

Marihiko fa fanampiana amin'ny fambolenao sy ny fivelomanao ihany ity voly vary teknika fa tsy hoe ny taninao rehetra akory dia tsy maintsy ho atao an'io jiaby, masomboly 32 kapoaka ihany koa aloha no andramana ho atao amin'ny taninao voalohany.

Question mahakasika ny familiarite-any amin'ilay tetik'asa: Manao ahoana ny fahafantaranao mikasika an'io voly vary teknika io ("tavy boka"? *Coding: (3: efa nanao mihitsy, 2: Efa nahita, 1: Efa nahare, 0: Sady mbola tsy nahita no mbola tsy nahare mihitsy)*

Manao ahoana ny fahitanao an'io voly vary teknika io? Izany hoe matoky ve ianao fa mety ho tsara izy io ary afaka hanampy anao amin'ny fivelomanao?

Refesina amina mari-drefy 1 ka hatramin'ny 5 (1 tena tsy mino, 3: misalalsala, 5: tena mino)

2. Comparer project (vary) avec argent (MGA 1,000,000):

Ianao anefa no tena mahafantatra izay tena mety amin'ny fivelomanao ary mety tsy tianao ilay voly vary teknika, ka manome safidy anao io mpamatsy vola io hoe afaka mahazo lelavola koa ianao ho takalon'ilay voly vary teknika.

---

[1] Ny vokatra moa dia tombanana ho eo amin'ny 1,5 a 2 tonnes per hectare eo (70 a 100 vata isaky ny hectare ny vokatra azo avy amina ambioka 1 vata – verifier-o sao tsy mitovy le unite, 1 vata akotry ~ 20 kg eo

Raha ianao izao no misafidy hoe vola 1,000,000 Ar sa ilay fanampiana voly vary teknika io, inona no tianao? Mila misafidy iraika ihany ianao. Ilay masombola dia afaka ampiasainao amin'izay tianao, na ivangana vary na hanaramana olona na izay safidimponao.

A	B
**Mandray vola** 1 tapitrisa ariary	Voly vary teknika

Raha nisafidy ilay vola dia inona no mety ampiasainao izany?

Hisy fanoharana be dia be hojerentsika ka fantaro fa izy ireo dia tsy mifampiankina fa samy hafa aby, izany hoe ilay safidinao amin'ilay fanoharana teo ohatra tsy voatery hitovy ary tsy misy ifandraisany amin'izay manaraka eo (_mbola mila averina azavaina indray ity rehefa isaky ny miova ilay choix atolotra azy_.

3. Introduire teviala

Amin'ity manaraka ity indray, andeha isika haminavina hoe afaka mahazo fahazoan-dalana hanano atiala ara-dalana amina faritr'ala eo amin'ny 1 hekara eo ianao, izy io izany dia sahala amin'ny ambioka vary iray vata (***aseho azy ilay sarina atiala***). Izy io izany dia sahala amin'ilay plan 5 taona tahaka ny tamin'ny andron-dRatsiraka.

Izany hoe afaka mahazo tany vaovao ianao, mbola malemy tsara ANKOATR'IREO tany efa ananao toy ny jinjan-drazana sy ny tanimbolinao. Marihiko fa izy io dia tsy mahakasika (tsy manova) ny fambolem-barinao amin'ny jinjan-drazana na taninao hafa fa FANAMPINY na tany vaovao ankoatra ireo efa anananao.

Raha ianao izao no mahazo alalana ara-dalana anao atiala amin'io 1 ha io, dia inona no hambolenao eo? Mandritra ny firy taona eo io no hambolenao an'io alohan'ny amelanao azy hiala sasatra?

Raha misafidy ianao hoe le VVT sa le atiala 1 ha dia inona no tianao?

A	B
Teviala 1 ha	Voly vary teknika

Raha toy izao manaraka izao indray ary ny safidy manoloana anao dia inona no tianao?

Io vola io dia hotantana-na sehatra tsy miankina iray izay ahafahanao mitahiry ny volanao (izany hoe anokafanao kaonty tahiry na boatin-drakitra). Io izany dia karazan'ny mpanelanelana anao sy ilay mpamatsy vola. Afaka tehirizinao ao foana ny volanao dia alainao rehefa misy ilaivanao azy.

A	B
**Mandray vola**: 6,000,000 Ar (6 tapitrisa)	Mahazo ilay voly vary teknika
Mandritra ny folo taona izany hoe 600,000 (6 hetsy) isan-taona	-
Tsy mahazo manano atiala mandrakizay (na any aoriana any aza)	Mahazo anilay teviala 1 ha

Marihiko fa ilay voly vary teknika zany ao amin'ilay *safidy B* dia afaka ataonao eo amin'ny tany efa anananao ihany na amin'ilay tany vaovao (atiala 1 ha) ilay vao ho azonao).

Marihiko koa fa indray mandeha monja no afaka mividy an'io teviala 1 ha io ianao fa tsy iverina intsony amin'ny manaraka, dia toy izay koa ilay voly vary teknika, fanampiana indray mandeha mitontona izy io fa tsy ho azonao isan-taona akory.

Andeha hotohizantsika ihany ity fanoharana[2] ity, hafa indray koa ity manaraka ity, tsy misy ifandraisany amin'ny teo aloha teo.

4. Introduire teviala malalaka

Amin'ty manaraka ity indray, fantaro fa mety misy koa fahafahanao manano atiala malalaka tsy misy fepetra. Izany hoe tsy misy intsony ny fanjakana na ny NGAP na ny tetik'asa mpanara-maso anao fa dia afaka manara-po manano izay vitanao ianao. Izy io izany dia sahala amin'ilay plan 5 taona tahaka ny tamin'ny andron-dRatsiraka izay nahazoan'ny raimandrenintsika teo aloha namonjy tany lonaka tsy misy fepetra.

Raha tahaka izao ny safidy manoloana anao dia inona indray no tianao:

A	B	C
Vola: 9 tapitrisa Ar no tontalin'ny vola raisinao	Tsisy vola	Teviala malalaka
Mandritra ny 10 taona (izany hoe 9 hetsy isan-taona)	---	
Tsy misy ilay fanampiana voly vary teknika	Mahazo ilay fanampiana voly vary teknika (masomboly 8 kg + fiofanana amle teknika + zezika + fanafody behatoka + fitaovana)	
Tsy mahazo manano atiala intsony mandrakizay ianao sy ny taranakao (ireo zaza mbola miray	Mahazo anilay teviala 1 ha (mbola asiana miaramila eo akaikiny midika fa tsy afaka manitatra mihoatra an'io 1 ha io zareo)	

[2] Raha tsy mety misafidy eo izy tonga dia sautio any amin'ny 4 (teviala malalaka)

trano aminao ato) – Asiana MIARAMILA!<br><br>(<u>*aleo ity tsy dia negativiser-na be fa azavaina hoe ato ry zareo mbola afaka mamondro (manofa) na miarana na mividy tany amin'olona fa tsy afaka manitatra manano atiala fotsiny*</u>*)		

***Fanamarihana*:** Fantaro fa raha tsy nahazo an'ilay alalana manano teviala ianao kanefa manano na dia 1 ara monja aza dia tamy ity rangaha (statuette miaramila) hisambotra anao (*sady aseho azy ilay miaramila*) ary dimy taona am-ponja farafahakeliny ny sazy miandry anao. Torak'izany koa, raha ohatra ianao misafidy an'ilay hoe mahazo alalana ara-dalana anano atiala amina 1 ha kanefa mihoatra anio fepetra 1 ha io na koa manao any ankafa tsy nahazoana alalana dia miditra am-ponja avy hatrany mandritra ny dimy taona farafahakeliny, izany hoe hisy mpanara-maso akaiky (*tondroina ilay statuette miaramila*) ny atiala.

Marihiko koa fa amin'ilay safidy voalohany (choix A), ilay hoe tsy mahazo alalana anano atiala na dia 1 ara monja aza dia ihatra MANDRAKIZAY izy io, izany hoe tsy afaka ny hanitatra hanano atiala intsony mihitsy ianao sy ny taranakao na amin'izao na amin'ny ho avy fa hikatona tanteraka mandrakizay io atiala io ary mety hisy mihitsy aza miaramila hitoby ao anaty ala ao hiambina an'io.

Ao tsara ve hatreto? Tsy misy fanontaniana? Raha tsy misy dia hotohizantsika foana ihany ty kilalaontsaina ity an. Aza adino fa mbola ato anatin'ialy kilalaon-tsaina na fanoharana foana isika izao.

5. Introduire timeframe

Amin'ity fanoharana ity indray, andeha ataontsika hoe tena tsy afaka ny manano atiala intsony mihisy ianao sy ny taranakao (**ireo zaza mbola miara-miaina aminao ato na rehefa lehibe aza izy ireo – fa ireo zanany efa manambady mahaleo-tena dia tsy voakasik'izay satria efa manana ny tokantranony manokana izy)**. Raha toa ka misy ny mpamatsy vola iray te anampy anao ary anome masombola anao mamaim-poana (tsy misy tamberiny tsy misy arakaraka), inona no tianao: vola 9 tapitrisa ariary ve anatin'ny 20 taona (izany hoe 5 alina sy 4 hetsy isan-taona mandritra ny 20 taona) (choix 1) sa ahazo 6 tapitrisa ariary ao anatin'ny 10 taona izany hoe 6 hetsy ariary isan-taona?

A	B
**Vola**: 9,000,000 Ar (9 tapitrisa)	**Vola**: 6,000,000 Ar (6 tapitrisa)
20 taona (5 alina sy 4 hetsy isan-taona)	10 taona (6 hetsy isan-taona)

Raha mbola tsy namely na tsy napetraka ity fanontaniana ity tery aloha dia afaka apetraka eto:

inona no mety ampiasainareo izany vola izany?

COMPREHENSION AND CERTAINTY

Comprehension codes (likert scale 1 to 5) – For the overall CE exercise – izany hoe am farany vao refesin'ny interviewer)

1: Tena tsy mahazo mihitsy. Tena tsy mety mihitsy

2: Vita ihany le follow-up fa betsaka ny diso, betsaka ny tsy azony

3: Netinety, azony ny ankapobeny fa hoatry ny nisy diso, nisy tsy azony ihany ny details

4: Azony ny zava-dehibe sady azony ilay details, amin'ny ankamaroany

5: Tena parfait, ao tsara, aony tsara daholo na ny ankapobeny na ny details

Certitude codes (isaky ny choice card ianao ry mpanao enquete no mila mandrefy azy ity)

1: Raha tena nisalasala be, ela be izy, sady niova ny choix-ny, mety hoe alternative A dia niova B avy eo dia sady mamerina manontany indray izy, ety tsy mahavita misafidy mihitsy izy

2: Nisalasala koa izy sady manontany zavatra, sady somary mila presser-na izy vao manao safidy sdy mety mbola mifindra

3: Nisalasala sady manontany fa rehefa nifidy izy dia hoatry ny resy lahatra ihany (fa ela2 vao nifidy)

4: Nisalasala kely,

5: tena tsy nisalasala mihitsy fa vao vita ny fanazavana dia nisafidy haingana

REHEFA VITA NY CHOICE CARDS:

Amin'ity farany ity indray dia fantaro fa mety misy koa tetik'asa hafa telo hafa ankoatr'ilay voly vary teknika afaka anampy anao amin'ny fivelomanao. Izany hoe tetikasa afaka ANAMPIANAO ny fivelomanao izy io fa tsy hoe asolo ny velon-tenanao amin'izao akory (*<u>Mila azavaina tsara hoe ankoatry ny velon-tena efa ataondry zareo amin'izao izy ireto fa tsy hoe asolo ny velontenany akory, izany hoe mbola afaka mamboly foana ry zareo</u>*):

- **Fambolena ovy voly maoderina**: masomboly voafantina (6 a 8 oviala sur un are) miampy ny fitaovana sy ny zezika ary ny fiofanana ilaina amin'izany. Amin'ny maha hoe masomboly voafantina sy fambolena maoderina azy moa dia mety mahatratra 5 à 12 tonnes à l'hectare ny vokatra.

- **Fiompiana akoho maoderina:** Akoho gasy vita vaksiny, salama tsara, miisa 5, 1 lahy, 4 vavy, miampy fiofanana sy fitaovana ary ny sakafo sahaza azy ireo mandritra ny telo volana. Anamboarana fahitra (tranony)
- **Fiompiana tantely maoderina:** vata maoderina miisa roa, miampy ny renin-tantely sy ny fisarihana ny tantely sy ny fitaovana hafa rehetra ilaina miampy ny fiofanana mandra-pioty vokatra. Hisy teknisiana ampianatra.
- **Voly vary teknika** ve: masomboly voafantina eo amin'ny 8 kg eo, miampy zezika sy fanafody bibikely ary fitaovana entinao miasa ny tany ary koa fiofanana ampianarana anao izany teknika vaovao izany, ny tanjona dia itazomana ny tsiron-tany mba tsy halady ho masina ny tany. Tahak'izao ny mety ho paozin'izany voly vary teknika izany (<u>*aseho sary*</u>), mety misy asa tany mila ataonareo mialoha.

Raha asain'ilay mpamatsy vola misafidy ianao hoe iza amin'ireto tetik'asa efatra ireo no tianao entinao manampy ny fivelomanao? Raha alahatra dia iza amin'ireo no tianao indrindra ary iza no tsy tinao indrindra?

FOLLOW-UP QUESTIONS

A. FANONTANIANA MAHAKASIKA ILAY FANOHARANA / KILALAON-TSAINA NATAONTSIKA TEO

1. a. Raha ataontsika hoe mahazo ilay fahazoan-dalana hanano atiala malalaka iny ny tokantranonao (izany hoe foana hatreo ny NGAP ary tsy misy fanjakana manara-maso intsony) dia mbola misy ve fitsipika ety ifotony izay mifehy hoe aiza ianao no afaka manano atiala? Izany hoe mbola misy ve ny fepetra na fitsipika amintsika samy isika ety ifotony sa afaka ny anara-po tokoa ianao? *Would there be still any traditional rules regulating teviala in the free teviala scenario where the government is not present?* (coding eny = 1, tsia = 0, -98= tsy fantatro, -99= tsy te hamaly ilay olona adiadiana)

   b. Raha misy fitsipika ifotony, inona izy io? (<u>*raiso naoty izay lazainy, raha tsy omby moa ny palce dia ao amin'ilay espace hoe fanamarihana*</u>)

   c. Niova ve io fitsipika ety ifotony io sa hoatr'izay foana taloha? *Have these rules changed compared to before?* (Coding eny = 1, tsia = 0, -98= tsy fantatro, -99= tsy te hamaly ilay olona adiadiana)

2. Mino ve ianao fa raha misy mpamatsy vola vahiny iray, tena te hitondra asa fampandrosoana ety aminareo dia METY hoe tena hanome vola anareo tokoa izy fa tsy karazana tetik'asa toy ny mahazatra toy ny fambolem-bary maoderina na tetik'asa fiompiana

tantely? / How plausible did you find the idea of a donor who is interested in development that would give you CASH instead of usual development projects (e.g. improved rice-growing technique)?

Refesina amina mari-drefy 1 ka hatramin'ny 5 (1: tena mino hoe tena hanome vola, 3: misalalsala, 5: tsy mino mihitsy hoe hanome vola izany ny Fanjakana *au lieu de projet*)

3. a. Manao ahoana ny fahatokisanao an'ilay rafitra mahaleo-tena na banky iray hanelanelana anao sy ilay Mpamatsy vola, izany hoe ilay hitantana sy afaka anokafanao ilay boatin-drakitra itehirizanao an'ilay vola? *Izany hoe matoky ve ianao fa ho ara-dalana sy tomombana tsara ny fitehirizany ny volanao? How much do you trust the independent institution which is to manage your money over time?*

Refesina amina mari-drefy 1 ka hatramin'ny 5 (1: tena tsy matoky, 3: misalalsala, 5: tena matoky)

b. Raha tsy matoky izy (1 na 2 amin'ilay mari-drefy), inona ohatra no rafitra hafa atokisany kokoa hitahiry ilay vola, izany hoe ilay mpanelanelana azy amin'ilay mpamatsy vola?

4. *Raha nisafidy matetika ilay anano atiala malalaka:* Mety azonao azavaina ve hoe inona no antony nisafidianao matetika ilay hoe anano atiala malalaka?

5. *Raha nisafidy matetika ilay hoe tsy anano atiala mihitsy* (migadra raha vao manano): Mety azonao azavaina ve hoe inona no antony nisafidianao matetika ilay hoe tsy hanano atiala mihitsy?

B. QUESTIONS MAHAKASIKA BELIEFS AND PERCEPTIONS

6. a. Mahita tombotsoa amin'ny fiarovana ny atiala ve ianao? Izany hoe mitondra raha tsara ho anao ve (na ny velontenanao) rehefa voaaro io atiala io? (coding eny = 1, tsia = 0, -98= tsy fantatro, -99= tsy te hamaly ilay olona adiadiana)

b. Raha eny, inona ohatra?

c. Tamin'ianao nisafidy ve, nieritreritra anireo tombotsoa ireo ianao? (Coding eny = 1, tsia = 0, -98= tsy fantatro, -99= tsy te hamaly ilay olona adiadiana)

Do you perceive any benefits from forest protection? If yes, can you give examples? When you made your choices, did you take these benefits into account?

7. Mino ve ianao fa mihaino ny sosonkevitra izay avoakan'ny mpikaroka ny Fanjakana eo amin'ny politika apetrany hitantanana ny atiala? *Consequentiality of the valuation exercise: do you believe that the results of this research would be used to inform policy on forest conservation?*

*Refesina amina mari-drefy 1 ka hatramin'ny 5 (*1: tena tsy mino, 3: misalalsala, 5: tena mino*)*

8. Mino ve ianao fa mihaino anareo mponina ety ifotony ny Fanjakana eo amin'ny politika apetrany itantanany ny ala na eo amin'ny fanampiana anareo eo amin'ny famelomantenanareo? Ohatra hoe ny hetahetanareo mba ahazo fanampiana noho ny tsy fahafahanareo manano atiala intsony na izay hetahetanareo hafa? *How much do you believe you would be able to negotiate compensations / or any other requests with the government?* (i.e. be heard upon and responded to by the government)?

   *Refesina amina mari-drefy 1 ka hatramin'ny 5 (*1: tena tsy mino, 3: misalalsala, 5: tena mino*)*

9. Araka ny hevitrao, inona no fomba / paikady mety sy mahomby entina mitantana ny atiala ary koa manampy ny sarambabem-bahoaka eo amin'ny fivelomana: Tsy maintsy hividy taratasy fahazoan-dalana ara-dalana afahafahanareo manano atiala ve sa rarana tanteraka ny teviala ary omena tambiny na vola ianareo? *(Coding: 1= omena tambiny (vola na tetik'asa), 0= hividy alalana anano atiala,* -98= tsy fantatro, -99= tsy te hamaly ilay olona adiadiana*). In your opinion, which forest management policy do you think is more legitimate for the whole society: each household being paid not to do teviala or everyone pays to be able to do teviala? (coding: 1= being compensated for not doing teviala, 0=pay and buy a teviala permit, -98=I don't know, -99= the respondent doesn't want to answer)*

C. QUESTIONS MAHAKASIKA NY EXPERIENCES-NDRY ZAREO EO AMIN'NY FAMPIHARANA NY LALANA MANDRARA NY TEVIALA

10. Efa nisy ve olona tato an-tokantranonareo niditra am-ponja na voasazy nandoa lamandy noho ny fanaovana atiala? *Eny / Tsia. (Coding* 98= tsy fantatro, -99= tsy te hamaly ilay olona adiadiana*, Eny = 1, Tsia = 0). Is there in your family anyone which has been penalized because of teviala practices? If yes, when, what was the sanctions?*

    Eny (1) / tsia (0)

    Raha "Eny", oviana? …………

    Firy?

    Inona ilay sazy? (nigatra nandritra ny …., nandoa vola…….)

11. Misy olona fantatrao ve eto amin'ny fokontany na faritra misy anareo niditra am-ponja na nandoa lamandy noho ny resaka atiala? *Eny / tsia. (Coding* 98= tsy fantatro, -99= tsy te hamaly ilay olona adiadiana*, Eny = 1, Tsia = 0). In the village territory or the FKT, do you know anyone who have been imprisoned or who have incurred sanctions because of teviala practices? If yes, When?*

    Eny (1) / tsia (0)

    Raha "Eny", oviana? …………

    Firy?

Inona ilay sazy? (nigatra nandritra ny …., nandoa vola…….)

12. a. Manao ahoana ny fiambenan'ny fanjakana na ny mpitantsoroka amin'ny fanjakana (NGAP) ny atiala amin'zao fotoana izao? (*This is only a dummy question to introduce 3b and 3c, write down any categorical answer, Ask the respondent to give examples of how strong or how weak it is*). Izany hoe matetika ve ny fisafoana, misy ve ny olona voasamboatra na voasazy vao haingana sns.

    b. Raha oharina amin'ny teo aloha, manao ahoana ny fiambenana an' io atiala io amin'izao fotoana izao? Nihamafy kokoa ve sa niha-malefaka kokoa sa mitovy ihany? Compared to now, how stronger or weaker was it in the past (*Le taloha resahana eto dia miankina amin'ilay agen-ilay mpamaly, mety io hoe 5 taona lasa, 10 taona lasa, 20 taona lasa, tsara anontaniana ilay mpamaly hoe taloha oviana ilay tiany zakaina*)? (Coding: -98= tsy fantatro, -99= tsy te hamaly ilay olona adiadiana, 2=mafy kokoa, 1=malefaka kokoa, 0=mitovy) *Tsara noter-na eo ambany ihany hoe taloha oviana no hi se referrervan'ilay adihadiana.*

    c. Araka ny fieritreretanao azy, mety anao ahoana ny fiarovana an'io atiala io amin'ny ho avy (ihamafy ve sa ihamalefaka kokoa sa mitovy amin'izao ihany)? Andeha ataontsika hoe hanao ahoana afaka 5 taona eo? In your opinion, do you think the protection will become stronger, weaker, or stay the same in the future, let's say in 5 years' time? (Coding -98= tsy fantatro, -99= tsy te hamaly ilay olona adiadiana, 2=mafy kokoa, 1=malefaka kokoa, 0=mitovy)

WILLINGNESS TO PAY QUESTIONNAIRE

WTP (izay en italique sy voatsipika dia fanazavana ho antsika interviewers fa tsy mila resahina ilay olona adihadiana)

Introduction (*atao mialoha ny HH QUESTIONNAIRE*):

- **FREE AND PRIOR INFORMED CONSENT (FPIC)**

Izahay dia mpianatra mpikaroka avy amin'ny Oniversiten'Antananarivo. Ny fianarana izay ataonay dia mikasika ny velontenantsika aty ambanivolo. Mety aharitra ora iray eo ny koranantsika. Ary hizara sokajy roa: Voalohany dia fanontaniana vitsivitsy mahakasika ny famelomantenan-dreo: fambolena, fiompiana, sy fivelomana hafa ataondreo itondrana vady aman-janaka, ary ny faharoa dia hanano karazana kilalaon-tsaina izay isika na an-kitsapaka, mbola mahakasika velon-tena ihany ary ho azavaiko misimisy rehefa avy eo. Ity pejy misy sary izay apetrakay aminareo ity moa dia manazava amin'ny antsipirihany hoe zovy moa izahay ary inona tsara ilay fikarohana ataonay (*project leaflet*). Aza manahy fa raha sendra tsy mahay mamaky teny moa isika ato antrano dia mbola ho azavainay misy2 miandalana eto ihany ny ao anatiny.

Voalohany indrindra dia te ahafantatra izahay raha mazoto sy malalaka ianareo ikorana aminay. Ny resaka ifanaovantsika moa dia an-tsitrapo sy malalaka tanteraka fa tsy terena akory.

Rehefa manaiky izy:

Misaotra mialoha ny amin'ny finiavana asehonareo. Eny antenantenany eny, afaka manapaka mametraka fanontaniana ianareo na mangataka ny hijanona. Raha toa koa ka misy fanontaniana tsy tianareo ho valiana dia zonareo tsara ny mizaka hoe tsy hamaly ary dia afaka miroso amin'ny fanontaniana manaraka isika.

Mazava hatreto? Misy fanontaniana ve na fanamarihana?

- **REASSURE RESPONDENT ABOUT ANONYMITY**

Fantaro fa fianarana / fikarohana ity ataonay ity, ary afaka mahazo toky isika fa ny valinteny omentsika eto na ny momba momba antsika dia tsy hozarainay na amin'iza na amin'iza fa mijanona ho tsiambaratelo. Ho raketinay amin'ny fomba hafa kely hany ny valintenintsika ka tsy misy afaka ny hahalala hoe zovy no nizaka inona. Izahay rahateo moa tsy maka anarana na hoe laharana karam-panondro izany. Afaka tena matoky isika noho izany fa tsy misy raha tokony ahavaka antsika ny koranantsika eto ary afaka tena mamboraka izay am-pontsika isika mahakasika ny fameloman-tenantsika. Tsara ny manarimaka fa ity fanadihadiana ity sy ny fitaovana ampiasainay dia tsy aty

amin'ny faritra misy anareo ihany no anaovanay azy fa efa nampiasaina tamin'ny faritra hafa teto Madagasikara hatrany ampitan-dranomasina.

- **WHAT IS RESEARCH?** (*Optional: Tsy voatery atao ety amboalohany fa afaka atao amin'ny farany koa na rehefa mametraka fanontaniana zareo ohatra hoe, inona ny voka-tsoa azo avy amin'ilay fanadihadiana, sns.).*

Izahay dia tena mpianatra / mpikaroka mahaleo-tena tanteraka ary tsy manao asa fampandrosoana. MARIHINAY fa izahay dia tena tsy misy ifandraisainy amin'ny ANGAP na ny fanjakana. Izany hoe tsy miankina amin'ny Fanjakana na koa hoe iraky ny Fanjakana ary tsy miara-miasa amin'ny NGAP fa MPIANATRA MPIKAROKA ihany, Izany hoe ny andraikitray dia azo lazaina ho mizara mizara dingana roa: 1) mangadihady sy mijery ny zava-misy ianantsika ety ifotony (tena mitety tokantrano tsiraikaraiky mht na lavitra na akaiky) ary rehefa vita izany dia 2) manoratra boky mikasika ny valim-pikarohana ary manolotra soson-kevitra ny fanjakana sy ny sehatra iraisam-pirenena. Ary dia anjaran'ireo ny mandinika sy manantanteraka asa fampandrosoana. Izahay kosa tsy afaka ny ampanantena fa mandainga si izahay raha mizaka izany.

- **RANDOM SAMPLING OF HOUSEHOLD AT THE VILLAGE LEVEL** (*Optional: afaka rehefa manontany zareo hoe ahoana ny fomba nisafidianana ny tokantrano vao azavaina)*

Ity fangadihadiana ataonay ity dia isan-tokantrano, ny atao hoe tokantrano moa dia ireo olona iray tafo, iray mahandro, iray fivelomana. Sitrakay tokoa raha ny tokantrano rehetra eto xxx no adiadianay saingy ny fotoana moa voafetra sady izahay koa efatra mianadahy si dia voatery ampahany si no vitanay. Izahay tsy hoe nifidy ireo ampahany ireo na nanavakavaka fa nataonay ankitsapaka, toy ny natao tanaty satroka ny anaram-bositra fiantsoana dia izay voaray tao tokantrano adihadianay.

ETO MANOMBOKA ILAY CE:

Amin'ity dingana manaraka ity izany ity dia hiara haminavina toe-javatra maromaro isika, izany hoe hijery fanoharana izay miendrika kilalaon-tsaina na ankitsapaka isika, ireo fanoharana ireo dia mahakasika ny velontenantsika.

Na dia hoe hiresaka betsaka ny hoe Fanjakana na ANGAP isika (*na MNP izahay ahalalandry zareo azy ny national park*) anatin'ity kilalaon-tsaina ity dia MARIHINAY fa izahay dia tena tsy misy ifandraisainy amin'ny ANGAP na ny fanjakana fa mahaleo-tena tanteraka izahay. Ilay Fanjakana na ANGAP horesahanay ato anatin'ity fanoharana ity dia ohatra ihany ampaisainay amin'ity fianarana ity, fa tsy

hoe izahay akory no Fanjakana. Raha haka ohatra iray izao isika anazavainay izay hoe maha- maha mahaleo-tena anay izay dia sady tsy mandrara anareo hanano atiala ohatra izahay no tsy mandrisika anareo hanano atiala koa etsy andaniny. Izany hoe tsy miandany izahay.

Ampahatsiahivinay fa tena fianarana / fisapan-kevitra ihany ity ataontsika manaraka ity.

PLEASE, REMIND RESPONDENT ABOUT ANONYMITY HERE (LE HOE TSY MAKA ANARANA SY TAZOMINA HO TSIAMBARATELO NY KORANA, TSY MIVOAKA NY RINDRINA EFATRA).

Koa manentana antsika aho araka izany hilaza ny safidy izay tena andrian'ny saintsika.

⇨ ***Remarque generale hafa hoa antsika interviewer***:

- *Ilay raimpianakaviana no tena mila mamaly, afaka hoe eo koa ilay vadiny manampy azy fa izy no tompokevitra farany,*
- *Mila rassurer-na be zareo eny am-boalohany hoe tsy anelingelina ny asa fivelomany akory isika fa ny fivelomany hatrizay mbola afaka ataony foana ihany fa tena hoe ivelany na fanampiana ilay efa velontena si no resahantsika anatin'ity kilalaon-tsaina ity.*
- *Isaky ny miova choice card dia preciser-o tsara foana hoe hafa indray koa ity manaraka ity, tsy misy ifandraisany amin'ny teo aloha. Izany hoe asaina ataony toy ny mbola tsy nisafidy mihitsy izy teo aloha.*
- *Angamba tsara preciser-na koa amin'ilay teviala 1 ha hoe opportunite indray mandeha monja io fa tsy hoe isan-taona akory dia afaka ividy anio foana. Dia tahak'izany koa ilay voly vary teknika, fanampiana indray mitontona fa tsy misy foana akory isan-taona.*
- *Tsara koa preciser-na raha nisafidy ilay tsy manano atiala izy hoe mandrakizay izy io ho azy sy ny taranany tsy afaka ny hanano intsony mihitsy fa sao dia mbola manantena hoe mbola afaka hividy an'ilay teviala 1 ha any aoriana any.*
- *Eo amin'ilay fampidirana anle voly vary teknika, mila normaliser-na ihany le izy fa tsy afaka ny hitompo teny fatratra be isika hoe tena hamokatra be io. Mety au contraire aza mantsy mety ho ratsy be ny vokatra*
- *Mahakasika "affordability": (hoe takatry ny fahefa-mividiny ve ilay izy sa tsia), isaky ny choice card (CC1 a CC6) raha ohatra izy nisafidy ilay mandoa vola dia mila anontaniana sy rappeler-na FOANA izy hoe izay atokisany fa tena laniny ihany no afaka safidiany.*

1. **Project:**

Andeha ataontsika hoe te hanampy antsika ny Fanjakana eo amin'ny fivelomantsika kanefa tsy manana ny enti-manana anantanterahana an'izany. Andeha ary isika haminaviana hoe misy fahafahantsika mividy tetik'asa iray hanampiana ny fivelomantsika, ny Fanjakana dia hanampy antsika hitady mpandraharaha izay ahafahantsika mividy io tetik'asa io. Andeha ataontsika hoe voly vary teknika io tetik'asa io. Ao anatin'izany voly vary teknika izany dia misy masomboly voafantina anaovana andrana omena anao eo amin'ny 30 kapoaka (3 kopy) eo, izany masomboly voanfantina izany moa dia masomboly mety amin'ny tany aty. Ankoatran'io masomboly io dia misy koa ny zezika biolojika ary ny fanafody behatoka ilaina amin'izany ary koa ny fiofanana na fampianarana antsika izany teknika vaovao izany (izany hoe hisy mihitsy teknisiana ho avy aty aminao ampianatra anao izany teknika izany). Omena fitaovana madinidinika koa ianao toy ny angady, rateau, fourche, sns. Izy io moa dia mety aby na vary an-tanety na horaka ary ny TENA TANJONA AMIN'IZY IO DIA HITAZOMANA ILAY TSIRON-TANY (mba tsy malady masina[3] ilay tany). Mazava tsara ve? _Aseho azy le sary voly vary teknika._ Mety misy asa tany sy asa angady (mamadika benja) mila ataonao mialohan'ny hambolena fa izany moa dia ho ampianarina anao (_aseho azy ilay sary ambany ankavia_ (ecobuage).

Marihiko fa fanampiana amin'ny fambolenao sy ny fivelomanao ihany ity voly vary teknika fa tsy hoe ny taninao rehetra akory dia tsy maintsy ho atao an'io jiaby, masomboly 32 kapoaka ihany koa aloha no andramana ho atao amin'ny taninao voalohany, ianao no misafidy ny toerana tinao anaovana azy.

Question mahakasika ny familiarite-any amin'ilay tetik'asa: Manao ahoana ny fahafantaranao mikasika an'io voly vary teknika io ("tavy boka"? *Coding: (3: efa nanao mihitsy, 2: Efa nahita, 1: Efa nahare, 0: Sady mbola tsy nahita no mbola tsy nahare mihitsy)*

Manao ahoana ny fahitanao an'io voly vary teknika io? Izany hoe matoky ve ianao fa mety ho tsara izy io ary afaka hanampy anao amin'ny fivelomanao?

Refesina amina mari-drefy 1 ka hatramin'ny 5 (1 tena tsy mino, 3: misalalsala, 5: tena mino)

Raha eny, mety mahaliana anao ve io tolotra voly vary teknika io? Izany hoe mety mazoto ve ianao hanefa vola mba ahazoanao an'io tolotra voly vary teknika io? (Coding -98= tsy fantatro, -99= tsy te hamaly ilay olona adiadiana, eny=1, tsia=0)

2. **Introduire teviala**

---

[3] Ny vokatra moa dia tombanana ho eo amin'ny 1,5 a 2 tonnes per hectare eo (70 a 100 vata isaky ny hectare na ambioka 1 vata – verifier-o sao tsy mitovy, 1 vata akotry ~ 20 kg eo

Amin'ity manaraka ity indray, andeha isika haminavina hoe afaka mividy fahazoan-dalana hanano atiala ara-dalana amina faritr'ala eo amin'ny 1 hekara eo ianao, izy io izany dia sahala amin'ny ambioka vary iray vata (***aseho azy ilay sarina atiala***). Izy io izany dia ka tahaka ilay plan 5 taona sahala ny tamin'ny andron-dRatsiraka.

Izany hoe afaka mividy tany vaovao ianao, mbola malemy tsara ankoatr'ireo tany efa ananao toy ny jinjan-drazana sy ny tanimbolinao ianao. Marihiko fa izy io dia tsy mahakasika (tsy manova) ny fambolem-barinao amin'ny jinjan-drazana na taninao hafa fa FANAMPINY na TANY VAOVAO ankoatra ireo efa anananao.

Raha ianao izao no mahazo io fahazoana alalana ara-dalana anao atiala amin'io 1 ha io, dia inona no hambolenao eo? Mandritra ny firy taona eo io no hambolenao an'io alohan'ny amelanao azy hiala sasatra?

Mitohy ihany ilay kilalaon-tsaina an.

Raha ianao izao no misafidy hoe hividy ilay tany vaovao 1 ha sa ilay fanampiana amina voly vary teknika, inona no maharisika anao kokoa? Marihiko fa indray monja no afaka mividy an'io teviala 1 ha io ianao fa tsy iverina intsony amin'ny manaraka, dia toy izay koa ilay voly vary teknika, fanampiana indray mandeha mitontona izy io fa tsy ho azonao isan-taona akory.

A	B
Teviala 1 ha	Voly vary teknika

Ary raha izao indray ny safidy manoloana anao: hoe hanefa vola dimy hetsy ariary (500,000) dia ahazo ilay tany vaovao 1 ha sa handoa vola 100,000 ariary dia ahazo ilay tolotra voly vary teknika, inona no safidinao[4]? Marihiko fa afaka efainao rehefa miaka-bokatra ny vola. Hisy rafim-panjakana andoavanao izany vola izany, marihiko fa hisy koa rafitra tsy miankina anara-maso izany mba ampangarahara azy tanteraka.

A	B
**Manefa Vola**: 500,000 Ar	**Manefa Vola**: 100,000 Ar
Mahazo anilay teviala 1 ha	Mahazo ilay fanampiana amina voly vary teknika

Andeha hotohizantsika ihany ity kilalaon-tsaina ity. Hisy fanoharana be dia be hojerentsika ka fantaro fa izy ireo dia tsy mifampiankina fa samy hafa aby, izany hoe ilay safidinao amin'ilay fanoharana voalohany teo dia tsy voatery hitovy ary tsy misy ifandraisany amin'izay manaraka eo (*mbola mila averina azavaina indray ity rehefa isaky ny miova ilay choix atolotra azy*.

[4] Raha tena tsy mety misafidy mihitsy izy de lazao hoe andeha ataontsika hoe raha ohatra ka anananao le enti-manana andoavana ireo vola, inona amin'ireo no tinao?

3. Introduire teviala malalaka

Amin'ty manaraka ity indray, fantaro fa mety misy fahafahanao mividy fahazoan-dalana hanano atiala malalaka tsy misy fepetra, izany hoe tsy voafetran'ilay 1 ha teo aloha. Izany hoe tsy misy intsony ny ANGAP na Fanjakana hiaro ny ala. Mitovy amin'ny plan 5 taona tamin'ny andron-dratsiraka fa saingy mila manefa vola amin'ny fanjakana ianao mba ahazoanao io alalana ara-dalana io. Aseho azy ilay sary.

Andeha hotohizantsika ihany ity fanoharana ity, hafa indray koa ity manaraka ity, tsy misy ifandraisany amin'ny teo aloha teo.

4. Introduire an example of choice card

Inona indray no tianao amin'ireto safidy manaraka ireto? Marihiko izany fa amin'ity indray mitoraka ity dia **IZAY AZONAO ANTOKA FA VITANAO NO IDIRANAO**. Izany hoe izay tena atokisanao hoe tena ho voaloanao tokoa (amin'ilay ohatra eto ambany izao dia hoe tena laniny tokoa ve ilay andoa 5 alina sy iray hetsy isan-taona mandritra ny 20 taona). Marihiko hatrany fa afaka efainao rehefa miaka-boatra ny vola.

A	B	C
Manefa Vola: 3 tapitrisa Ar	Manefa Vola 500,000 (5 hetsy Ar)	Tsy misy vola alohanao ary tsy mahazo alalana anano atiala ianao (mihidy tanteraka ny atiala – misy miaramila mpiambina ao) – <u>*aleo ity tsy dia negativiser-na be fa azavaina hoe ato ry zareo mbola afaka mamondro (manofa) na miarana na mividy tany amin'olona fa tsy afaka manano atiala fotsiny*</u>
Afaka aloha anatin'ny 20 taona (5 alina sy 1 hetsy isan-taona)	Aloha indray mandeha monja	
Tsy misy ilay tetik'asa voly vary teknika	Mahazo tetik'asa voly vary teknika	
Malalaka ny teviala	Mahazo anilay teviala 1 ha	

i. <u>*Raha nanao safidy an-tsitrapo izy tsy mila noterena dia iny izany no choix ampidirina anaty fichier excel. Indraindray nefa lazainy fa tsy misy tiany ilay alternative telo atolotra azy ka raha sendra an'izay dia ilay alternative hoe mijanona amin'izao, mihidy tanteraka ny atiala no recorder-na ary asiana asterisk (eto izao dia "C*") - (rehefa mampiditra data dia 0 izy rehefa choix an-tsitrapo fa 1 izy rehefa safidy noterena)*</u>

<u>**Raha mazoto ny hividy ilay fahazoan-dalana ilay tokantrano dia miditra amin'ny 5:*</u>

5. Introduire timeframe

Raha ianao indray izao no maniry hividy io taratasy fahazoan-dalana manano atiala malalaka io. Iza no tianao? Hanefa vola 1,500,000 ariary anatin'ny 20 taona ve (7,5000 na dimy arivo sy fito alina) sa handoa 1,000,000 ariary anatin'ny 10 taona (100,000 ar) isan-taona.

A	B
Manefa vola 1,500,000 Ar	Manefa vola 1,000,000 Ar
Aloha anatin'ny 20 taona (75,000 na dimy arivo sy fito alina)	Aloha anatin'ny 10 taona (100,000 ar) isan-taona
Mahazo an'ilay fahazoan-dalana hanano atiala malalaka	

***Fanamarihana**:* Fantaro fa raha tsy nahazo an'ilay alalana manano teviala ianao kanefa manano na dia 1 ara monja aza dia tamy ity rangaha (statuette miaramila) hisambotra anao (*sady aseho azy ilay miaramila*) ary dimy taona am-ponja farafahakeliny ny sazy miandry anao. Torak'izany koa, raha ohatra ianao misafidy an'ilay hoe mahazo alalana ara-dalana anano atiala amina 1 ha kanefa mihoatra anio fepetra 1 ha io na koa manao any ankafa tsy nahazoana alalana dia miditra am-ponja avy hatrany mandritra ny dimy taona farafahakeliny, izany hoe hisy mpanara-maso akaiky (*tondroina ilay statuette miaramila*) ny atiala.

Marihiko koa fa amin'ilay hoe tsy mahazo alalana anano atiala na dia 1 ara monja aza dia ihatra mandrakizay izy io, izany hoe tsy afaka ny anano atiala intsony mihitsy ianao ary tsy afaka ny hanantena hoe aoriana kely ao mety mbola afaka hanano fa hikatona tanteraka mandrakizay io atiala io ary mety hisy mihitsy aza miaramilam-panjakana hatory ao anaty ala ao hiambina an'io.

Ao tsara ve hatreto? Misy fanontaniana? Raha tsy misy dia hotohizantsika foana ihany ty kilalaontsaina ity an. Aza adino fa mbola ato anatin'ialy kilalaon-tsaina foana isiska izao.

Averiko hatrany fa ny valinteny omentsika eto na ny anarantsika dia ho tsy hozaraina na amin'iza na amin'iza ary ho raketiny amin'ny fomba hafa kely hany ka tsy misy afaka ny hahalala hoe iza no nizaka inona. Koa manentana antsika aho araka izany hilaza ny safidy izay tena andrian'ny saintsika.

COMPREHENSION AND CERTAINTY

Comprehension codes (likert scale 1 to 5) – For the overall CE exercise – izany hoe am farany vao refesin'ny interviewer)

1: Tena tsy mahazo mihitsy. Tena tsy mety mihitsy

2: Vita ihany le choix rehetra fa betsaka ny diso, betsaka ny tsy azony

3: Netinety, azony ny ankapobeny fa hoatry ny nisy diso, nisy tsy azony ihany ny details

4: Azony ny zava-dehibe sady azony ilay details, amin'ny ankamaroany

5: Tena parfait, ao tsara, azony tsara daholo na ny ankapobeny na ny details

Certitude codes (isaky ny choice card ianao ry mpanao enquete no mila mandrefy azy ity)

1: Raha tena nisalasala be, ela be izy, sady niova ny choix-ny, mety hoe alternative A dia niova B avy eo dia sady mamerina manontany indray izy, ety tsy mahavita misafidy mihitsy izy

2: Nisalasala koa izy sady manontany zavatra, sady somary mila presser-na izy vao manao safidy sdy mety mbola mifindra

3: Nisalasala sady manontany fa rehefa nifidy izy dia hoatry ny resy lahatra ihany (fa ela2 vao nifidy)

4: Nisalasala kely,

5: tena tsy nisalasala mihitsy fa vao vita ny fanazavana dia nisafidy haingana

REHEFA VITA NY CHOICE CARDS:

Amin'ity farany ity indray dia fantaro fa mety misy koa tetik'asa telo hafa ankoatr'ilay voly vary teknika afaka anampy anao amin'ny fivelomanao ary afaka vidinao amina mpandraharaha iray izay miara-miasa amin'ny Fanjakana. Izany hoe tetikasa afaka ANAMPIANAO ny fivelomanao izy io fa tsy hoe asolo ny velon-tenanao amin'izao akory (*<u>Mila azavaina tsara hoe ankoatry ny velon-tena efa ataondry zareo amin'izao izy ireto fa tsy hoe asolo ny velontenany akory, izany hoe mbola afaka mamboly foana ry zareo</u>*):

- **Fambolena ovy voly maoderina**: masomboly voafantina (6 a 8 oviala sur un are) miampy ny fitaovana sy ny zezika ary ny fiofanana ilaina amin'izany. Amin'ny maha hoe masomboly voafantina sy fambolena maoderina azy moa dia mety mahatratra 5 à 12 tonnes à l'hectare ny vokatra.

- **Fiompiana akoho maoderina:** Akoho gasy vita vaksiny, salama tsara, miisa 5, 1 lahy, 4 vavy, miampy fiofanana sy fitaovana ary ny sakafo sahaza azy ireo mandritra ny telo volana. Anamboarana fahitra (tranony)
- **Fiompiana tantely maoderina:** vata maoderina miisa roa, miampy ny renin-tantely sy ny fisarihana ny tantely sy ny fitaovana hafa rehetra ilaina miampy ny fiofanana mandra-pioty vokatra. Hisy teknisiana ampianatra.
- **Voly vary teknika** ve: masomboly voafantina eo amin'ny 8 kg eo, miampy zezika sy fanafody bibikely ary fitaovana entinao miasa ny tany ary koa fiofanana ampianarana anao izany teknika vaovao izany, ny tanjona dia itazomana ny tsiron-tany mba tsy halady ho masina ny tany. Tahak'izao ny mety ho paozin'izany voly vary teknika izany (<u>*aseho sary*</u>), mety misy asa tany mila ataonareo mialoha.

Raha misafidy ianao hoe inona amin'ireto tetik'asa efatra ireo no mahaliana anao entinao manampy ny fivelomanao? Raha alahatra dia iza amin'ireo no tianao indrindra ary iza no tsy tinao indrindra?

FOLLOW-UP QUESTIONS

D. QUESTIONS MAHAKASIKA ILAY FANOHARANA / KILALAON-TSAINA NATAONTSIKA TEO:

1. a. Raha ataontsika hoe mahazo ilay fahazoan-dalana hanano atiala malalaka iny ny tokantranonao (izany hoe foana hatreo ny NGAP ary tsy misy fanjakana manara-maso intsony) dia mbola misy ve fitsipika ety ifotony izay mifehy hoe aiza ianao no afaka manano atiala? Izany hoe mbola misy ve ny sakana na fitsipika amintsika samy isika ety ifotony sa anara-po tokoa ianao? *Would there be still any traditional rules regulating teviala in the free teviala scenario where the government is not present?* (coding eny = 1, tsia = 0, -98= tsy fantatro, -99= tsy te hamaly ilay olona adiadiana)

   b. Raha misy fitsipika ifotony, inona izy io? (<u>*raiso naoty izay lazainy, raha tsy omby moa ny palce dia ao amin'ilay espace hoe fanamarihana*</u>)

   c. Niova ve io fitsipika ety ifotony io sa hoatr'izay foana taloha? *Have these rules changed compared to before?* (coding eny = 1, tsia = 0, -98= tsy fantatro, -99= tsy te hamaly ilay olona adiadiana)

2. Tanatin'ilay kilalaon-tsaina teo isika dia niresaka hoe hoe Fanjakana hamarotra an'ilay taratasy fahazoan-dalana anano atiala mba hanampiana antsika amin'ny velontenantsika: METY tena ho tanteraka ve izany araka ny hevitrao? *Likelihood of the State selling permit in the valuation exercise:* How plausible did you find the idea of the government selling you a permit to do teviala to help you with your livelihooods?

Refesina amina mari-drefy 1 ka hatramin'ny 5 (1 tena tsy mino hoe ho tanteraka izany, 3: misalalsala, 5 tena matoky hoe ho tanteraka izany).

3. Andeha ataontsika hoe nahazo ilay taratasy fahazoan-dalana ara-dalana hanano atiala ianao, matoky ve ianao fa ho hajain'ny Fajakana izany fahazoan-dalana malalaka izany? Izany hoe hanan-kery foana ilay taratasy azonao / hitohy mandrakizay izany? *If the government had sold you permit, would you trust the government to honour that permit forever?*

Refesina amina mari-drefy 1 ka hatramin'ny 5 (*(*1: tena matoky, 3: misalalsala, 5: tena tsy matoky).

4. *Raha nisafidy matetika ilay anano atiala malalaka:* Mety azonao azavaina ve hoe inona no antony nisafidianao matetika ilay hoe anano atiala malalaka?
5. *Raha nisafidy matetika ilay hoe tsy anano atiala mihitsy* (migadra raha vao manano): Mety azonao azavaina ve hoe inona no antony nisafidianao matetika ilay hoe tsy hanano atiala mihitsy?

E. QUESTIONS MAHAKASIKA BELIEFS AND PERCEPTIONS

6. a. Mahita tombotsoa amin'ny fiarovana ny atiala ve ianao? Izany hoe mitondra raha tsara ho anao ve na ny famelomantenanao rehefa voaaro io atiala io? (coding eny = 1, tsia = 0, 98= tsy fantatro, -99= tsy te hamaly ilay olona adiadiana)

   b. Raha eny, inona ohatra?

   c. Tamin'ianao nanao ireo safidy teo ireo ve, nieritreritra anireo tombotsoa ireo ianao? (coding eny = 1, tsia = 0, 98= tsy fantatro, -99= tsy te hamaly ilay olona adiadiana)

   Do you perceive any benefits from forest protection? If yes, can you give examples? When you made your choices, did you take these benefits into account?

7. Mino ve ianao fa mihaino ny sosonkevitra izay avoakan'ny mpikaroka ny Fanjakana eo amin'ny politika apetrany hitantanana ny atiala? *Consequentiality of the valuation exercise: do you believe that the results of this research would be used to inform policy on forest conservation?*

*Refesina amina mari-drefy 1 ka hatramin'ny 5 ((*1: tena tsy mino, 3: misalalsala, 5: tena mino*))*

8. Mino ve ianao fa mihaino anareo mponina ety ifotony ny Fanjakana eo amin'ny politika apetrany itantanany ny ala na fangatanareo mikasika velon-tena? Ohatra hoe ny fanirianareo mba hividy fahazoan-dalana ara-dalana hanano atiala na izay hetahetanareo hafa mba hanatsarana ny fivelomanareo? How much do you believe you would be able to negotiate teviala permit / or any other requests with the government (i.e. be heard upon and responded to by the government)?

*Refesina amina mari-drefy 1 ka hatramin'ny 5 (*1: tena tsy mino, 3: misalalsala, 5: tena mino*)*

9. Araka ny hevitrao, inona no fomba / paikady mety sy mahomby entina mitantana ny atiala ary koa manampy ny sarambabem-bahoaka eo amin'ny fivelomana: Tsy maintsy hividy taratasy fahazoan-dalana ara-dalana afahafahanareo manano atiala ve sa rarana tanteraka ny teviala ary omena tambiny: tetikasa na vola ianareo? *(coding: 1= omena tambiny (vola na tetik'asa), 0= hividy alalana anano atiala,* 98= tsy fantatro, -99= tsy te hamaly ilay olona adiadiana*).*

F. FANONTANIANA MAHAKASIKA NY EXPERIENCES-NDRY ZAREO EO AMIN'NY FAMPIHARANA NY LALANA MANDRARA NY TEVIALA

10. Efa nisy ve olona tato an-tokantranonareo niditra am-ponja na voasazy nandoa lamandy noho ny fanaovana teviala? *Eny / Tsia. (Coding* -98= tsy fantatro, -99= tsy te hamaly ilay olona adiadiana*, Eny = 1, Tsia = 0). Is there in your family anyone which has been penalized because of teviala practices? If yes, when, what were the sanctions?*

Eny (1) / tsia (0)

Raha "Eny", oviana? …………

Firy?

Inona ilay sazy? (nigatra nandritra ny …., nandoa vola…….)

11. Misy olona fantatrao ve eto amin'ny fokontany niditra am-ponja na nandoa lamandy nandritra izany vanim-potoana izany? *Eny / tsia. (Coding* -98= tsy fantatro, -99= tsy te hamaly ilay olona adiadiana*, Eny = 1, Tsia = 0). In the village territory or the FKT, do you know anyone who have been imprisoned or who have incurred sanctions because of teviala practices? If yes, When?*

Eny (1) / tsia (0)

Raha "Eny", oviana? …………

Firy?

Inona ilay sazy? (nigatra nandritra ny …., nandoa vola…….)

12. a. Manao ahoana ny fiambenan'ny fanjakana na ny mpitantsoroka amin'ny fanjakana ny atiala amin'zao fotoana izao? (*This is only a dummy question to introduce 3b and 3c, write down any categorical answer, Ask the respondent to give examples of how strong or how weak it is*). Izany hoe matetika ve ny fisafoana, misy ve ny olona voasamboatra na voasazy vao haingana sns.

b. Raha oharina amin'ny teo aloha, manao ahoana ny fiambenana an' io amin'izao fotoana izao? Nihamafy kokoa ve sa niha-malefaka kokoa sa mitovy ihany? Compared to now, how stronger or weaker was it in the past (*<u>Le taloha resahana eto dia miankina amin'ilay agen-ilay</u>*

mpamaly, mety io hoe 5 taona lasa, 10 taona lasa, 20 taona lasa, tsara anontaniana ilay mpamaly hoe taloha oviana ilay tiany zakainy)? (Coding: -98= tsy fantatro, -99= tsy te hamaly ilay olona adiadiana, 2=mafy kokoa, 1=malefaka kokoa, 0=mitovy) *Tsara noter-na eo ambany ihany hoe taloha oviana no hi se referrervan'ilay adihadiana.*

c. Araka ny fieritreretanao azy, mety anao ahoana ny fiarovana an'io atiala io amin'ny ho avy (ihamafy ve sa ihamalefaka kokoa sa mitovy amin'izao ihany)? Andeha ataontsika hoe hanao ahoana afaka 5 taona eo? In your opinion, do you think the protection will become stronger, weaker, or stay the same in the future, let's say in 5 years' time? (Coding -98= tsy fantatro, -99= tsy te hamaly ilay olona adiadiana, 2=mafy kokoa, 1=malefaka kokoa, 0=mitovy)

CHOICE EXPERIMENT SURVEY IN PRACTICE

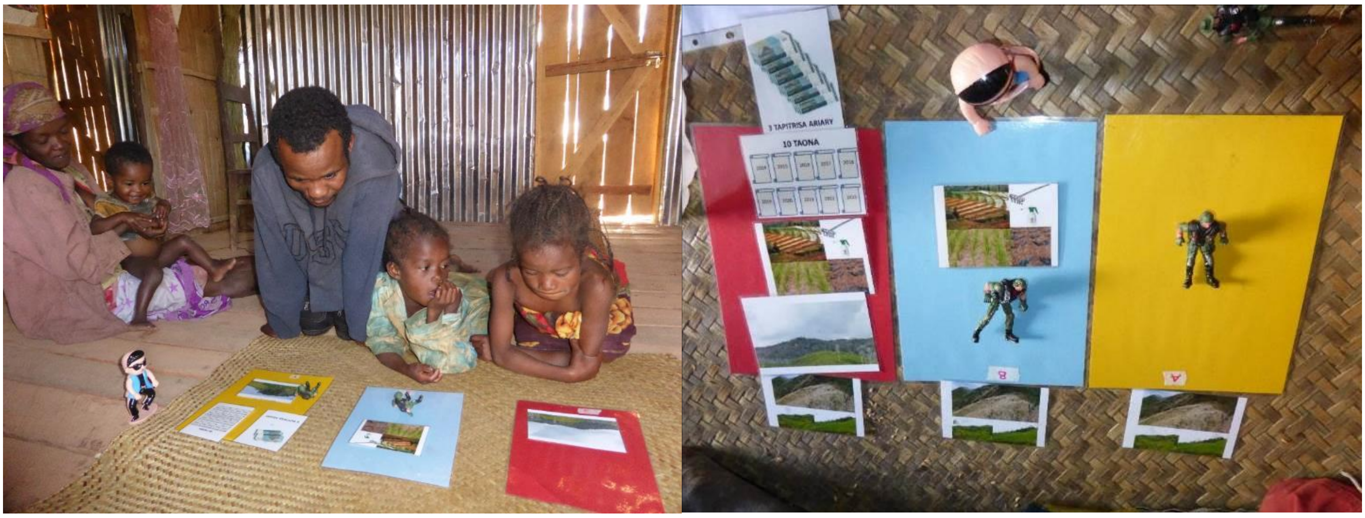

Supplement: Supplemental Information 10 — (household survey and choice experiment in English and Malagasy). [file peerj-06-5106-s010.pdf]
