# Supplementary material for: Who bears the cost of forest conservation?
Source: PeerJ. 2018 Jul 5;6:e5106. doi: 10.7717/peerj.5106 (PMC6035863; doi:10.7717/peerj.5106)
Supplement: Supplemental Information 11 — (Agricultural survey in English and Malagasy). [file peerj-06-5106-s011.pdf]

Supplementary materials: Who bears the cost of forest conservation? Survey instruments for phase three of data collection: the contingent valuation

We present the survey instruments used (in English as well as Malagasy)

p4ges can paying 4 global ecosystem services reduce poverty?

les paiements pour les services écosystémiques globaux peuvent-ils réduire la pauvreté? www.p4ges.org

Safeguards Follow-Up Survey Interviewer's Script

(English translation – in case of any discrepancies the Malagasy version should be treated as definitive).

(text in *italics* is explanation for the interviewers, and is not to be read out)

Introduction (to be done before the QUESTIONNAIRE):

NB Respondents were also provided with a copy of the general p4ges project information sheet to keep.

FREE AND PRIOR INFORMED CONSENT (FPIC)

"We are university students and researchers who aim to better understand your livelihoods. This interview may last about one hour, and is divided into two parts: first, a few questions concerning those projects that you received recently as support to your livelihoods. And the second part is a sort of exercise or game, still relating to those projects, and which I will explain further shortly. This page with a picture on it ***[the general p4ges project information sheet]*** that I will leave with you explains who we are and more about the research we are doing. Do not worry if perhaps you cannot read, I will still explain in more detail what is in it.

First of all, we would like to know whether you are willing and available to be interviewed by us. The discussions we will have are entirely voluntary and you are not compelled to take part.

[If they agree]

Thank you in advance for your willingness to take part. You are welcome to interrupt to ask a question or ask for the interview to stop. If there are any questions you would prefer not to answer, then it is your right not to answer them, and we can move onto the next one.

Is that clear up to now? Do you have any questions or clarifications?

REASSURE RESPONDENT ABOUT ANONYMITY

You should know that this is research we are doing, and you have a guarantee that your responses and your details will not be shared with anybody but will remain our secret. Your responses will be identified only by a number, not a name, so that nobody can find out who said what. Therefore we will not be collecting names or ID card numbers. Therefore you can trust that that there is no reason to be concerned about this interview, and you can say what you really wish to about your livelihoods. It is good to note that this research and these materials we use aren't only being used here but have been used elsewhere in Madagascar and overseas.

WHAT IS RESEARCH? (*Optional: It is not essential to do this section – it can be done at the end or if they ask questions e.g. about the benefits that will come from this study).*

We are completely independent students / researchers and are not engaged in development. We emphasise that there is no link between us and the government or CI. That is we do not depend on the government nor represent the government, and we are not colleagues of CI but only students/ researchers. That is our role is twofold: 1) investigate and observe the situation on the ground (we are visiting individual households whether near or far), and when that is completed 2) write a report about our findings and present suggestions to the government and international institutions. And it is their responsibility to consider these and to implement development activities. WE, on the other hand, cannot raise expectations – we would be lying if we did that.

SAMPLING OF HOUSEHOLD AT THE VILLAGE LEVEL (*Optional: this can be used if they ask how they came to be selected)*
This interview we are conducting is at the household level, (where household means those people who live under one roof, cook together and share a livelihood). We would like to interview everybody in [community name] however, we have a limited time, and are only two people, therefore we can only manage to cover some households. Only those people who received projects are included because we are interested in how the projects have gone, that is how you came to be selected.

[Start Questionnaire here up to end of Projects Section D. Sections A-E on pp1-4 only conducted with HHs not in the original HH survey'
From "Warmup", use text below:]

[Here begins the contingent valuation:]
In this next part, we will do a kind of exercise or game about your livelihoods and the projects you received. The objective of this is to find out what the best way is to help the livelihoods of rural people in the future. That is to say, we would like to find out how you value those projects when you consider what you received. We emphasise that this is only a study.

[PLEASE, REMIND RESPONDENT ABOUT ANONYMITY HERE.]

We therefore hope that you will give your true responses.

⇨ ***General remark for interviewers:***
- *It is the head of household who should respond, their spouse can help them but it is they ultimately decide.*

Explanation of certitude codes (for each money card)
1: very uncertain, changed his/her choice many times, asked the interviewers to re-explain the choice cards again one or two more time, took ages to finally make up his/her mind
2: uncertain, the respondent took also very long but least a little bit quicker than 1
3: so, so, seems a bit uncertain but at least when s/he made his choices eventually, s/he seemed certain.
4: certain of his/her choice, the respondent was quick and didn't request any additional explanations
5: no doubt at all, relatively quick, made up his mind after the first round of explanation.

certainty	*1*	*2*	*3*	*4*	*5*

Respondents' rated comprehension (scale 1 to 5) – For the overall CE exercise – to be measured by the enumerator)
1: The respondent doesn't understand the CE valuation exercise at all, all the choices seem inconsistent, barely finished the choice cards
2: Many inconsistencies but at least finished all the choice cards
3: So so, the respondent seems to understand the generalities but still missed the details, still made few inconsistent choices
4: In general, good understanding of the details and generalities, made only one- two inconsistent choices 4

5: Perfect, understands very well everything
.

comprehension	*1*	*2*	*3*	*4*	*5*

Introduction to the valuation exercise

[NB: Don't refer explicitly to the last time they conducted the choice experiment – if they mention it, say it is similar to that one but simpler, and about a completely different subject – we don't want them anchoring on their responses, or thinking it's about teviala]

As per what I explained previously, it is the value that you attach to the project that we are trying to establish with this exercise. It is worth noting that this exercise is not real, but a tool we are using to understand how well the projects went. That is to say that you may see references to money in this exercise but I would like to emphasise that we are not giving out money, the money only exists in the exercise. Now I will explain more about the exercise.

Warmup
I'd like you to think back to the time when you first got the project. [*say whatever they chose and lay down the card representing that project]*. Imagine that you knew then what you now know about this project.

[Ask warm up questions 1 & 2 (see questionnaire)]
[Clear away all cards]

[After Warmup Question 2:]
I would like to return again to the time when you first received the project. Imagine that instead of the projects, you had been offered money that you could spend howsoever you wished on improving your family's livelihood. This money would be managed by an independent institution where you could keep the money (that is you would open a savings account). This would therefore be a sort of intermediary between you and the donor of the money. You can keep the money there, and you can withdraw it whenever you need it.

Do you understand about this money? Once again, this is only an exercise, we are not giving out money.
[answer any questions they have about the money, payment vehicle etc]

[return to questionnaire here]

Payment Card

start time payment card:

I would still like you to think about that meeting where you chose this project. We're still discussing that decision-making. What we would like to know, if you could return and change your choice, would you still choose the project, or money? So, we will do a kind of exercise to help you make up your mind.
This is how the exercise will work: I guess you've already thought about how much money might replace the project you got. I will show you next a card showing the pricture of the project you received, which I will put down here, then I there are several cards that show different sums of money (that I will shuffle here) to be arranged next to the project card. If you think that you would prefer the money to the project, then put the card showing the money above the card showing the project, but if you would prefer to choose the project instead of the money, then put the money card below the card showing the project, but if you are really uncertain then put the money card alongside the project card. Note that I'm shuffling the money cards before we come to arranging them, then I will deal them out at random.

[Record the results in the table on the questionnaire]

end time payment card:

[Continue with follow up questions from questionnaire]

can paying 4 global ecosystem services reduce poverty?

les paiements pour les services écosystémiques globaux peuvent-ils réduire la pauvreté? www.p4ges.org

Safeguards Follow-up Survey Interviewer's Script

(Malagasy version i.e. definitive)

(izay en italique dia fanazavana ho antsika interviewers fa tsy mila resahina ilay olona adihadiana)

Introduction (atao mialoha ny QUESTIONNAIRE):

NB Respondents were also provided with a copy of the general p4ges project information sheet to keep.

FREE AND PRIOR INFORMED CONSENT (FPIC)

Izahay dia mpianatra mpikaroka avy amin'ny Oniversiten'Antananarivo. Ny fianarana izay ataonay dia mikasika ny velontenantsika aty ambanivolo. Mety aharitra ora iray eo ny koranantsika. Ary hizara sokajy roa: Voalohany dia fanontaniana vitsivitsy mahakasika ilay tetikasa izay azonareo tety ho fanampiana ny velon-tenanareo voa taona vitsivitsy monja izay, ary ny faharoa dia hanano karazana kilalaon-tsaina izay isika na an-kitsapaka, mbola mahakasika ireo tetikasa ireo ihany ary ho azavaiko misimisy rehefa avy eo. Ity pejy misy sary izay apetrakay aminareo ity moa dia manazava amin'ny antsipirihany hoe zovy moa izahay ary inona tsara ilay fikarohana ataonay ***[the general p4ges project information sheet]***. Aza manahy fa raha sendra tsy mahay mamaky teny moa isika ato antrano dia mbola ho azavainay misy2 miandalana eto ihany ny ao anatiny.

Voalohany indrindra dia te ahafantatra izahay raha mazoto sy malalaka ianareo ikorana aminay. Ny resaka ifanaovantsika moa dia an-tsitrapo sy malalaka tanteraka fa tsy terena akory.

[Rehefa manaiky izy:]

Misaotra mialoha amin'ny finiavana asehonareo. Eny antenantenany eny, afaka manapaka mametraka fanontaniana ianareo na mangataka ny hijanona. Raha toa koa ka misy fanontaniana tsy tianareo ho valiana dia zonareo tsara ny mizaka hoe tsy hamaly ary dia afaka miroso amin'ny fanontaniana manaraka isika.

Mazava hatreto? Misy fanontaniana ve na fanamarihana?

REASSURE RESPONDENT ABOUT ANONYMITY

Fantaro fa fianarana / fikarohana ity ataonay ity, ary afaka mahazo toky isika fa ny valinteny omentsika eto na ny momba antsika dia tsy hozarainay na amin'iza na amin'iza fa mijanona ho tsiambaratelontsika. Ho raketinay amin'ny fomba hafa kely ny valinteninareo (laharana fa tsy anarana) hany ka tsy misy afaka ny hahalala hoe zovy no nizaka inona. Izahay rahateo moa tsy maka anarana na hoe laharana karam-panondro izany. Afaka tena matoky isika noho izany fa tsy misy raha

tokony ahavaka antsika ny korana eto ary afaka tena mamboraka izay am-pontsika isika mahakasika ny velontenatsika. Tsara ny manarimaka fa ity fanadihadiana ity sy ny fitaovana ampiasainay dia tsy aty amin'ny faritra misy anareo ihany no anaovanay azy fa efa nampiasaina tamin'ny faritra hafa teto Madagasikara hatrany ampitan-dranomasina.

WHAT IS RESEARCH? (*Optional: Tsy voatery atao ety amboalohany fa afaka atao amin'ny farany koa na rehefa mametraka fanontaniana zareo ohatra hoe, inona ny voka-tsoa azo avy amin'ilay fanadihadiana, sns.).*
Izahay dia tena mpianatra / mpikaroka mahaleo-tena tanteraka ary tsy manao asa fampandrosoana. MARIHINAY fa izahay dia tena tsy misy ifandraisainy amin'ny ny fanjakana na CI. Izany hoe tsy miankina amin'ny Fanjakana na koa hoe iraky ny Fanjakana ary tsy miara-miasa amin'ny (CI) fa MPIANATRA MPIKAROKA ihany, Izany hoe ny andraikitray dia azo lazaina ho mizara mizara dingana roa: 1) mangadihady sy mijery ny zava-misy ianantsika ety ifotony (tena mitety tokantrano tsiraikaraiky mht na lavitra na akaiky) ary rehefa vita izany dia 2) manoratra boky mikasika ny valim-pikarohana ary manolotra soson-kevitra ny fanjakana sy ny sehatra iraisam-pirenena. Ary dia anjaran'ireo ny mandinika sy manantanteraka asa fampandrosoana. Izahay kosa tsy afaka ny ampanantena fa mandainga si izahay raha mizaka izany.

SAMPLING OF HOUSEHOLD AT THE VILLAGE LEVEL (*Optional: afaka rehefa manontany zareo hoe ahoana ny fomba nisafidianana ny tokantrano vao azavaina)*
Ity fangadihadiana ataonay ity dia isan-tokantrano, ny atao hoe tokantrano moa dia ireo olona iray tafo, iray mahandro, iray fivelomana. Sitrakay tokoa raha ny tokantrano rehetra eto xxx no adihadianay saingy ny fotoana moa voafetra sady izahay koa roa mianadahy si dia voatery ampahany si no vitanay. Izahay tsy hoe nifidy ireo ampahany ireo na nanavakavaka fa izay olona nahazo tetikasa taty aminareo ihany no mahalianay satria te ahafantatra ny fadehan'ireo tetikasa azonareo taty izahay kai zany no nahataviditra anao anatin'izay fanadihadiana izao.

[Start Questionnaire here up to end of Projects Section D. Sections A-E on pp1-4 only conducted with HHs not in the original HH survey'
From "Warmup", use text below:]

[ETO MANOMBOKA ILAY contingent valuation:]
Amin'ity dingana manaraka ity dia hanao karazana kilalaon-tsaina mahakasika ny velontenantsika sy ny tetikasa izay azonareo isika. Ny tena tanjon'ilay zavatra ataontsika eto moa izany dia te ahafantatra izahay hoe inona ny fomba tsara indrindra ho entina manampy ny velon-tenantsika ety ifotony amin'ny hoavy.
Izany hoe te-ahafantatra izahay ny sandany homenao io tetikasa io raha mijery ireo zavatra azonao rehetra ianao. Ampahatsiahivinay fa tena fianarana na fitsapa-kevitra ihany ity ataontsika manaraka ity.

[PLEASE, REMIND RESPONDENT ABOUT ANONYMITY HERE (LE HOE TSY MAKA ANARANA SY TAZOMINA HO TSIAMBARATELO NY KORANA, TSY MIVOAKA NY RINDRINA EFATRA).]

Koa manentana antsika aho araka izany hilaza ny safidy izay tena andrian'ny saintsika.

⇨ ***Remarque generale hafa hoa antsika interviewer**:*

- *Ilay raimpianakaviana no tena mila mamaly, afaka hoe eo koa ilay vadiny manampy azy fa izy no tompokevitra farany,*

Certitude codes (isaky ny money card)

1: Raha tena nisalasala be, ela be izy, sady niova ny choix-ny, mety hoe ambony dia niova ambany avy eo dia sady mamerina manontany indray izy, ety tsy mahavita misafidy mihitsy izy

2: Nisalasala koa izy sady manontany zavatra, sady somary mila presser-na izy vao manao safidy sady mety mbola mifindra

3: Nisalasala sady manontany fa rehefa nifidy izy dia hoatry ny resy lahatra ihany (fa ela2 vao nifidy)

4: Nisalasala kely,

5: tena tsy nisalasala mihitsy fa vao vita ny fanazavana dia nisafidy haingana

certainty	*1*	*2*	*3*	*4*	*5*

Comprehension codes (likert scale 1 to 5) – For the overall CE exercise – izany hoe am farany vao refesin'ny interviewer)

1: Tena tsy mahazo mihitsy. Tena tsy mety mihitsy

2: Vita ihany le follow-up fa betsaka ny diso, betsaka ny tsy azony

3: Netinety, azony ny ankapobeny fa hoatry ny nisy diso, nisy tsy azony ihany ny details

4: Azony ny zava-dehibe sady azony ilay details, amin'ny ankamaroany

5: Tena parfait, ao tsara, azony tsara daholo na ny ankapobeny na ny details

comprehensio n	*1*	*2*	*3*	*4*	*5*

Introduction to the valuation exercise

[NB: Don't refer explicitly to the last time they played the kilalao-ntsaina – if they mention it, say it is similar to that one but simpler, and about a completely different subject – we don't want them anchoring on their responses, or thinking it's about teviala]

Araky ny nambarako teo moa izany fa ny sandany omenao ilay tetikasa no tena zavatra hotadiavintsika amin'ity kilalaon-tsaina ity. Tsara ho marihina antrany moa fa tsy zavatra tena misy ilay kilalao fa fitaovana ho entinay manombana ny fandehan'ilay tetikasa efa azonareo. Izany hoe mety ahita resaka vola isika eto dia tiako no manamarika fa tsy hizara vola izahay fa ao atin'ilay kilalao ihany no misy safidy vola. Rehefa avy eo izaho manazava misimisy kokoa mahakasika ilay kilalao.

Warmup

Tiako ianao hiverina hieritreritra any amin'ilay fotoana nahazoanao ilay fanampiana tamin'ny voalohany. [say whatever they chose and lay down the card representing that project]. Izao ianareo asaiko mieritreritra hoe raha nahafantatra ny fizotran'ilay tetikasa amin'izao fotoana izao.

[Ask warm up questions 1 & 2 (see questionnaire)]
[Clear away all cards]

[After Warmup Question 2:]
Averiko indray mande fa tiako ianao hiverina hieritreritra any amin'ilay fotoana nahazoanao ilay fanampiana tamin'ny voalohany. Izao ianareo asaiko mieritreritra hoe raha nahafantatra ny fizotran'ilay tetikasa amin'izao fotoana izao dia ho mety manao ahaona ny safidinareo vaovao. Ka amin'izany eritrereto hoe raha tokony nahazo ilay tetikasa ianao dia vola no natolotra anao ho solony mba hoentina hanatsarana ny velon-tenanao.
Io vola io dia hotantana-na sehatra tsy miankina iray izay ahafahanao mitahiry ny volanao (izany hoe anokafanao kaonty tahiry na boatin-drakitra). Io izany dia karazan'ny mpanelanelana anao sy ilay mpamatsy vola. Afaka tehirizinao ao foana ny volanao dia alainao rehefa misy ilaivanao azy.

Azonao tsara ve mahakasika ilay vola teo? Mbola averoko iany fa kilalao izy ity fa tsy hizara vola izahay tsy akory.
[answer any questions they have about the money, payment vehicle etc]

[return to questionnaire here]

Payment Card

start time payment card:

Mbola tiako ianao mieritreritra momba ilay fivoriana izay nifidiananao ilay tetikasa. Mbola mihevitra isika momba izany fandikana-kevitra izany. Ny tianay hofantatra dia, rahaohatra ka afaka miverina manova ny safidinao ianao dia, mbola nifidy an'ilay tetikasa ve sa te-hifidy vola? Dia, manao karazana kilalalao isika mba manampy anao manapa-kevitra.
Otran'izao ny fandehan'ilay kilalao, mino aho fa efa mety voatombanao izao izay ilay vola mety mahasolo ilay tetikasa efa azonao. Ka asehoko anao manaraka eto misy karatra maneho ilay sarin'ny tetikasa efa azonao izay apetrako eto ambany eto, aveo dia misy karatra maromaro misy maribola isan'karazany (izay akorotako eto) alahatra manoloana ilay tetikasa. Ka raha itanao fa raha aleonao nifidy ny vola toy izay ny tetikasa dia apetrao eo ambon'ilay karatra misy ilay tetikasa ilay karatra misy ilay vola fa raha itanao aleonao nifidy tetikasa toy izay vola dia apetrao eo amban'ilay karatra misy ilay tetikasa ilay karatra misy ilay vola fa raha tena misalasala ianao dia apetrakao milahatra amin'ilay tetikasa ilay karatra misy ilay vola. Tsara ho marihina fa ireto karatra misy ny vola ireto dia akorotako alohan'ny andaharana azy eto anoloana eto ka aveo dia atao antsapaka ny fakana ilay karatra aveo.

[Record the results in the table on the questionnaire]

end time payment card:

[Continue with follow up questions from questionnaire]
