# Supplementary material for: Who bears the cost of forest conservation?
Source: PeerJ. 2018 Jul 5;6:e5106. doi: 10.7717/peerj.5106 (PMC6035863; doi:10.7717/peerj.5106)
Supplement: Supplemental Information 12 — Contingent valuation of the compensation recieved by housholds (in English and Malagasy). [file peerj-06-5106-s012.pdf]

Supplementary materials: Who bears the cost of forest conservation? Survey instruments for phase two of data collection: the agricultural survey

We present the survey instruments used (in English as well as Malagasy)

can paying 4 global ecosystem services reduce poverty?

les paiements pour les services écosystèmiques globaux peuvent-ils réduire la pauvreté? www.p4ges.org

Work Package 6: Agricultural Questionnaire Survey

Survey Information

Activity/Task	Date	Start Time	End Time	Person(s) Responsible	Remarks
Interview					
Checking Questionnaire					
Coding Questionnaire					
Data Entry					
Checking and Approving Data Entry					

A. Identification

[Note: All the information in the table below, except for the name & PID of the respondents should be filled prior to the survey based on the initial HH survey data from the respective HH.]

1. HH Name & Code	*(name)*	*(HHID)*
2. Village Name & Code	*(name)*	*(VID)*
3. Fokontany Name & Code	*(name)*	*(FID)*
4. Commune Name & Code	*(name)*	*(CID)*
5. District Name & Code	*(name)*	*(DID)*
6. Name & PID of primary respondent	*(name)*	*(PID)*
7. Name & PID of secondary respondent	*(name)*	*(PID)*
8. GPS Location of the HH	*(Lat)*	*(Lon)*
9. Distance of HH from village centre	*(Minutes)*	*(Km)*

Respondent(s)

[Note: the respondent should normally be the HH head, but important additional information could be gained from talking to other adult respondents (e.g. Previous users of the land if inherited)]

[If suitable respondents are too old/infirm to walk to the plot, area measurements and inputs/outputs for latest year should be undertaken at plot with younger HH member, and plot history completed in HH dwelling]

[Notes on respondents here (if not HH head) i.e. who gave what information]

B. Land Ownership; Land Access; and Land Use

1. Information about land use

[Some of these information will have been obtained from the HH survey, and can be reproduced here. Interviewer should double check that these are all of the plots owned or used by the respondent]

Field number	Site name	Distance from Dwelling	Estimated Size	Type	Ownership/Access [For 'savoka', ask if the plot belongs solely to the HH or it is commonly held within the larger family or lineage]	Rented/Bought/ Borrowed from?	Cost/Payment details for rented and bought plots		Cultivated in most recent agricultural year?	Plot Age	Rotation Period
				(1=hill tavy; 2=flat tavy; 3=tanimbary; 4=tanimboly; 5=savoka; 6=other (specify))	*(1=inherited; 2= rented (specify); 3=bought (specify); 4=borrowed (specify); 5=cleared by the HH, 6=other (specify))*	**[If the plot is rented, bought, or borrowed, specify whether it is from the family or others] (1=family; 2=others)**	*Payment Type (Cash/ Kind)*	*Amount*	*(whether the plot was cultivated in 2013/2014) (1=YES; 0=NO)*	**[Fill this later based on the plot-specific survey information]**	*(time between most recent cultivation)* ***[if currently fallow, ask & record how long it has been fallow for]***

Remarks

2. Tavy System

[Note: Follow the pointers in each question carefully, especially on the 'YES/NO' questions as responses on those questions dictate which questions to ask and which ones to skip in this section.]

a. What limits the area of tavy land you cultivate each year?

[Prompt until no further factor is mentioned. Rank factors according to importance (top 3 only if more than 3 mentioned)]

**List of factors**	**Mark 'X' if Mentioned**	**Rank [THREE most important]**
Labour availability (HH labour/money to hire labour)		
Access to fertilizer		
Access to seed		
Fertility of existing land		
Cannot get fresh land through teviala: customary restrictions		
Cannot get fresh land through teviala: government restrictions		
Cannot get fresh land through teviala: land scarcity/population pressure		

b. What limits the productivity of the tavy land that you farm?

[Prompt until no further factor is mentioned. Rank factors according to importance (top 3 only if more than 3 mentioned)]

**List of factors**	**Mark 'X' if Mentioned**	**Rank [THREE most important]**
Labour availability		
Access to fertilizer		
Access to seed		
Fertility of existing land		
Pests (mice, insects, birds)		
Weeds		
Rainfall		

c. Have you obtained additional land for tavy in the last 5 years?

[ ] YES >> go to '3.' [ ] NO >> go to 'd.'

d. Have you considered obtaining additional land for tavy?

[ ] YES >> go to 'e.' [ ] NO >> go to 'f.'

e. If YES, what are the main factors stopping you?

[Prompt until no further factor is mentioned. Rank factors according to importance (top 3 only if more than 3 mentioned)]

**List of factors**	**Mark 'X' if Mentioned**	**Rank [THREE most important]**
There is no land for sale		
Theer is no land to rent		
I don't have enough labour to farm more land		
I don't have enough capital (money to buy/rent land)		
I cannot do teviala to obtain new land		
Immigrants are using land		
Population of village is growing too much		

f. If NO, why are you not considering obtaining additional land for tavy?
[Prompt until no further factor is mentioned. Rank factors according to importance (top 3 only if more than 3 mentioned)]

**List of factors**	**Mark 'X' if Mentioned**	**Rank [THREE most important]**
There is no land for sale		
Theer is no land to rent		
I don't have enough labour to farm more land		
I don't have enough capital (money to buy/rent land)		
I cannot do teviala to obtain new land		

3. Tanimbary

[Note: Follow the pointers in each question carefully, especially on the 'YES/NO' questions as responses to those questions dictate which questions to ask and which ones to skip in this section.]

a. Do you have tanimbary plot(s)?

[ ]YES >> go to 'b.'   [ ] NO >> go to 'l.'

b. Did you establish any of your Tanimbary plots within the last THREE years?

[ ] YES >> go to 'c.'   [ ] NO >> go to 'e.'

c. If 'YES', which year? **[Tick as appropriate]**

[ ] 2012   [ ] 2013   [ ] 2014

d. What were the main one-off costs to your household in establishing this Tanimbary? >> go to 'h.'

**Categories**	**Costs**	
	Labour [total mandays]	*Monetary*
Land acquisition		
Land preparation (building bunds, terracing etc)		
Infrastructure (building canals, locks for water diversions etc.)		
Other costs (specify)		
Other costs (specify)		
Other costs (specify)		
Other costs (specify)		

e. Have you recently increased the amount of tanimbary you have?

[ ] YES >> go to 'h.'   [ ] NO >> go to 'f.'

f. Have you considered increasing it in the future?

[ ] YES >> go to 'h.'   [ ] NO >> go to 'g.'

g. What are the main factors stopping you from increasing the area of tanimbary?

[prompt until no further factor is mentioned. Rank factors according to importance (top 3 only if more than 3 mentioned)]

**List of factors**	**Mark 'X' if Mentioned**	**Rank [THREE most important]**
Land availability		
Labour availability		
Capital (money to buy/rent land)		
Too much water (flooding)		
Too little water (drought)		
Investment in barrages/irrigation		
Skill/knowledge (fahaizana)		

h. What crops do you plant in your tanimbary?

[For multiple tanimbary plots, mark all that apply]

[ ] A single rice crop
[ ] 2 rice crops
[ ] 3 rice crops
[ ] rice & a contra-season crop (name)
[ ] other combination(s) (specify)

i. Have you considered planting more crops per year?

[ ] YES >> go to 'j.'
[ ] NO

j. If 'YES', what are the main reasons for not planting multiple crops per year?

[prompt until no further factor is mentioned. Rank factors according to importance (top 3 only if more than 3 mentioned)]

**List of factors**	**Mark 'X' if Mentioned**	**Rank [THREE most important]**
Labour availability		
Too much water (flooding)		
Too little water (drought)		
Access to fertilizer		
Temperature		

k. What limits the productivity of the tanimbary you have?

[prompt until no further factor is mentioned. Rank factors according to importance (top 3 only if more than 3 mentioned)]

**List of factors**	**Mark 'X' if Mentioned**	**Rank [THREE most important]**
Labour availability		
Too much water (flooding)		
Too little water (drought)		
Access to fertilizer		
Irrigation infrastructure (barrages etc)		
Pests		
Weed		

l. What are the main reasons why you don’t have tanimbary?
[prompt until no further factor is mentioned. Rank factors according to importance (top 3 only if more than 3 mentioned)]

**List of factors**	**Mark 'X' if Mentioned**	**Rank [THREE most important]**
Land availability		
Labour availability		
Capital (money to buy/rent land)		
Too much water (flooding)		
Too little water (drought)		
Investment in barrages/irrigation		
Skill/knowledge (fahaizana)		

m. Have you considered farming in tanimbary in the future?
[ ] YES >> go to sub-section '4.' [ ] NO >> go to 'n.'

n. What are the main factors stopping you?
[prompt until no further factor is mentioned. Rank factors according to importance (top 3 only if more than 3 mentioned)]

**List of factors**	**Mark 'X' if Mentioned**	**Rank [THREE most important]**
Land availability		
Labour availability		
Capital (money to buy/rent land)		
Too much water (flooding)		
Too little water (drought)		
Investment in barrages/irrigation		
Skill/knowledge (fahaizana)		

4. Productivity: Tavy vs Tanimbary
a. Which type of agriculture is more productive (value of crop per unit area)?
[ ] Tavy (from forest) [ ] Tavy (land which has been long cleared) [ ] Tanimbary

b. Which type of agriculture is more productive (value of crop per effort needed in an agricultural season)?
[ ] Tavy (from forest) [ ] Tavy (land which has been long cleared) [ ] Tanimbary

C. Livestock inputs and outputs

1. Livestock [Input/Output]

	**Current Number [double check against HH survey]**		**Inputs [during the past 12 months or period specified for the survey]**				**Output [during the past 12 months or period specified for the survey]**		
	From 1st HH survey	*Verification*	*Where pastured?*	*Feed*	*Medicine*	*Other costs [specify]*	*Consumption*	*Sale (animals)*	*Sale (Produce)*
Omby									
Kisoa									
Akoho									
Gana									
Gisa									
Ondry									
Bee hives									
Fish tanks/ponds									
Hafa [inona]									
Hafa [inona]									
Hafa [inona]									

D. Off-farm Income

1. Apart from farming, what were the main sources of income in your household during the last farming year (2013/2014)? What period did those income flow and how much?

Persons Involved	Sources*	Directly or indirectly related to park or protected area?	Total time involved [specify days or months]	Rate [specify daily, weekly or monthly rate]	Total Income
[Identify the persons involved as HH head or in relation to HH head, and indicate whether the person is an adult (M or F) or a child]	[Specify the source using the code below the table. If a person indicated in the previous column earns income from more than one source, use a separate row to record information for each source of income.]	[Record with 'YES' or 'NO' whether the source of income in the previous column is directly or indirectly influenced by park or protected area, including any environmental/conservation-related interventions]	[Calculate and record the total number of days worked here for the work that are paid by day. For salaried jobs or jobs paid by months, record total number of months worked.]	[Record the daily wage rate or monthly salary as appropriate clearly specifying the timeframe. For example, ___MGA/day; ____MGA/ month]	[If respondents directly report this, for example for 'Remittance', record the figure; for others calculate from the previous two columns and record here.

* Some key sources: 1=Daily wage (agricultural); 2=Daily wage (other in-village labour, e.g., cleaning, house building); 3=Daily wage (outside village); 4=Logging (lumberjack); 5=Artisan; 6=Service (govt. or private sector job); 7=Remittance, 8=others (specify).

1=spouse(legally married or co-habiting); 2=son/daughter; 3=son/daughter in-law; 4=grandchild; 5=mother/father; 6=mother/father in-law; 7=brother/sister; 8=brother/sister in-law; 9=uncle/aunt; 10=nephew/neice; 11=step/foster child; 12=other family; 13=not related

E. Questions about teviala

[Note: best to ask these questions at the end of the day? Avoid if overly sensitive?]

1. Do you or your HH have the rights to do Teviala in this area?

[   ] YES >> go to Q 2.   [   ] NO >> go to Q 3.

2. Are these rights limited to specific areas on forest?

[   ] YES   [   ] NO

3. If Teviala is being practised or used to be practised in this area, what insitutions regulate, or used to regulate, where, and when HH could do teviala (e.g. Tangalamena, head of lineage)? ***[Record any additional information in the box below the table]***

Teviala practices	Regulating Institution(s)	Where one could ddo teviala?	When one could do teviala?	If cannot practice now, why not?
[Record whether 'currently practised' OR 'previously practised']	[ask & list relevant institutions, both formal & informal]	[ask & record both location and types of land in terms of tenure situation	[ask & record timing as well as conditions that would have to be met to be able to do teviala]	[ask & record about the restrictions in place and the authorities involved in setting/enforcing the restrictions]

[Record any additional information about Teviala practices in the box below]

Work Package 6: Agricultural Questionnaire Survey	FID: ______	HHID: ______

[Start a new sheet for each field]

A. Basic info about the field and interviews

[If some of these information already recorded from the HH survey, try to verify here]

1. Field number (from previous sheet)	
2. Field location (local toponym or description of site/location)	
3. Does the HH have formal tenure rights (*vita borne)?*	
4. Specify the kind of tenure rights you have over this plot (e.g., owned in common, private ownership, customary use rights)	
5. Was this field cultivated by HH in most recent (2013/2014) agricultural year?	
6. If not, might any other HH have rights to use this field?	
7.If 'YES', who?	
**[Note: if not cultivated in most recent year, only measure/estimate size and collect clearance/cultivation history]**	
8. Field visited by researcher and respondent(s)? (1=YES; 0=NO)	
9. Field visited but too overgrown to measure? (1=YES; 0=NO)	

B. Location and Area

1. GPS boundary waypoints (all plots)

**GPS make and number**	**Waypoints of field boundary (e.g. 37-43, or 37, 39-43)**

2. deducted reckoning details (small plots only) ***[Add/remove extra lines as necessary]***

	**magnetic compass bearing**	**Distance (with wheel or pacing)**
Start point		
1		
2		
3		
4		
5		
6		
7		
8		
9		
10		
end point back to start		

3. Estimated relative size (for fields not visited)

[If a field is too far away to visit, or too overgrown to measure, size can be estimated by the respondents (if not visited) or the researcher and respondents (if overgrown) relative to fields which have been measured]

**Comparator Field Number**	**Relative size of this field to comparator (in %)**

C. Plot history

1. First clearance & cultivation history: when was this plot first cleared from the forest (ala velona) and by whom? ***[Use key events (cyclones, presidents, French colonisation etc)]***

**[(Non-)response codes: -98=respondent did not know; -99=respondent did not want to answer; -100=did not ask (specify why)]**	**[use multiple columns to redord additional details if reported]**		
a. When did the HH start farming this plot?			
b. Was the plot cleared by members of the HH? (If 'YES', who?)			
c. If not, who cleared the plot?			
d. Relation of original clearer to HH head			
e. How did the household acquire the plot?	**[This information should already be available from the main agricultural HH survey sheet (section B.). Use this opportunity to verify/ correct the information in the main agricultural survey sheet.]**		
f. Year of first clearance (best guess)			
g. Range of uncertainty (if respondent is uncertain, give range of possible years)			
h. Did the clearer have a permit from the Foret, or have to pay a bribe			
i. If 'YES', give details			

2. Any other details about the plot & cultivation history

[use the space below to record any additional information about the plot that is important/relevant to our survey]

3. Crops planted & cropping frequency

a. Starting with the most recent farming year on this plot, please tell us which years have you cultivated in this plot and the crops you have grown, for as far back as you can remember.

	**Latest farming season (________AD)**	**________ AD**	**________ AD**	**________ AD**	**________ AD**		
What crops were planted (when cultivated by the respondent)							

b. How many more times do you think this plot was cultivated before your household started farming in this plot and/or before you can remember? [ ]

c. In your knowledge/experience, has there been any change in the number of years this plot is usually left to fallow between cultivated years since this plot was first cleared? If 'YES', could you tell us the usual fallow length in early years and now?

Fallow length in early years	
Fallow length now	

D. Agricultural Inputs and Outputs

1. Agricultural Inputs - Labour: tell us about the labour used to cultivate this plot during the 2013/2014 agricultural year.

[Note: Use the space under ONE from 'Tavy System', 'Tanimboly' OR 'Tanimbary' as appropriate for this plot, and ignore the other two.]

**Tavy System**	**Period**	**HH Labour**			**External Labour**			**Other Expenditure**
		Men	*Women*	*Children*	Labour	*Payment Type* ***[Record whether in-kind/Cash/ Exchange]***	*How much?* ***[Record the quantity & unity for 'in-kind'; amount for 'cash']***	**[Ask and record any other labour-related expenditure here]**
Kapakapa (Slash)		_____ No / _____ days	_____ No / _____ days	_____ No / _____ days	_____ No / _____ days			
Mandoro (Burn)		_____ No / _____ days	_____ No / _____ days	_____ No / _____ days	_____ No / _____ days			
Mamboly		_____ No / _____ days	_____ No / _____ days	_____ No / _____ days	_____ No / _____ days			
Miava [1]		_____ No / _____ days	_____ No / _____ days	_____ No / _____ days	_____ No / _____ days			
Miava [2]		_____ No / _____ days	_____ No / _____ days	_____ No / _____ days	_____ No / _____ days			
Miava [3]		_____ No / _____ days	_____ No / _____ days	_____ No / _____ days	_____ No / _____ days			
Miandry Fody		_____ No / _____ days	_____ No / _____ days	_____ No / _____ days	_____ No / _____ days			
Misangom-bary		_____ No / _____ days	_____ No / _____ days	_____ No / _____ days	_____ No / _____ days			
Mitango katsaka		_____ No / _____ days	_____ No / _____ days	_____ No / _____ days	_____ No / _____ days			
Mitango tsaramaso		_____ No / _____ days	_____ No / _____ days	_____ No / _____ days	_____ No / _____ days			
Mitaona vary		_____ No / _____ days	_____ No / _____ days	_____ No / _____ days	_____ No / _____ days			
Mively vary		_____ No / _____ days	_____ No / _____ days	_____ No / _____ days	_____ No / _____ days			
Hafa (inona)		_____ No / _____ days	_____ No / _____ days	_____ No / _____ days	_____ No / _____ days			
Hafa (inona)		_____ No / _____ days	_____ No / _____ days	_____ No / _____ days	_____ No / _____ days			
Hafa (inona)		_____ No / _____ days	_____ No / _____ days	_____ No / _____ days	_____ No / _____ days			
Hafa (inona)		_____ No / _____ days	_____ No / _____ days	_____ No / _____ days	_____ No / _____ days			
**Tanimboly**								
Miava		_____ No / _____ days	_____ No / _____ days	_____ No / _____ days	_____ No / _____ days			
Taillage		_____ No / _____ days	_____ No / _____ days	_____ No / _____ days	_____ No / _____ days			
Maka vokatra [___________]		_____ No / _____ days	_____ No / _____ days	_____ No / _____ days	_____ No / _____ days			
Maka vokatra [___________]		_____ No / _____ days	_____ No / _____ days	_____ No / _____ days	_____ No / _____ days			
**Tanimbary**								
Mamadika bainga/ benja		_____ No / _____ days	_____ No / _____ days	_____ No / _____ days	_____ No / _____ days			
Mampiditra rano		_____ No / _____ days	_____ No / _____ days	_____ No / _____ days	_____ No / _____ days			
Miosy/Mamakivaky		_____ No / _____ days	_____ No / _____ days	_____ No / _____ days	_____ No / _____ days			
Planage		_____ No / _____ days	_____ No / _____ days	_____ No / _____ days	_____ No / _____ days			
Manetsa		_____ No / _____ days	_____ No / _____ days	_____ No / _____ days	_____ No / _____ days			
Miava [1]		_____ No / _____ days	_____ No / _____ days	_____ No / _____ days	_____ No / _____ days			
Miava [2]		_____ No / _____ days	_____ No / _____ days	_____ No / _____ days	_____ No / _____ days			
Miandry Fody		_____ No / _____ days	_____ No / _____ days	_____ No / _____ days	_____ No / _____ days			
Mijinja		_____ No / _____ days	_____ No / _____ days	_____ No / _____ days	_____ No / _____ days			
Mitaona vary		_____ No / _____ days	_____ No / _____ days	_____ No / _____ days	_____ No / _____ days			
Mively vary		_____ No / _____ days	_____ No / _____ days	_____ No / _____ days	_____ No / _____ days			
Hafa (inona)		_____ No / _____ days	_____ No / _____ days	_____ No / _____ days	_____ No / _____ days			
Hafa (inona)		_____ No / _____ days	_____ No / _____ days	_____ No / _____ days	_____ No / _____ days			
Hafa (inona)		_____ No / _____ days	_____ No / _____ days	_____ No / _____ days	_____ No / _____ days			

2. Agricultural Inputs - Materials & Other Inputs

**Main Crops**	**Inputs**										
	Seed		*Manure*		*Fertilizer*		*Pesticide*		*Animals*	Hired equipment	*Other materials*
	**Quantity & Unit [Ask and record Quantity & Unit]**	**Price [Ask and record price/unit if bought]**	**Use manure? [Record 'YES' or 'NO']**	**If 'YES' was there sufficient manure from the livestock owned?**	**Quantity & Unit [Ask and record Quantity & Unit]**	**Price [Ask and record price/ unit]**	**Quantity & Unit [Ask and record Quantity & Unit]**	**Price [Ask and record price/unit]**	**[If hired draught animals were used in land preparation, ploughing, ask and record the cost of renting them]**	**[If other machinery/equipment were hired, ask and record the cost involved here]**	**[Ask and record any other cost associated with the farming of this plot here]**

3. Agricultural Outputs - Yield, Consumption, Sale

	**Outputs**								
	Total yield	*How would you rate this yield compared to typical yield from this plot?*	*If the yield has not been typical, how much would be the typical yield in this plot relative to this yield?*	*HH consumption*	*Sale*		*In-kind Payment*	*Seed*	
**Main crops**	**[Record Reported Quantity & Unit]**	**[Get the respondents to rate the yield on 5-points scale: 1=very low, 2=low, 3=typical, 4=high, 5=very high]**	**[Record the relative variation in yield from the typical yield as %]**	**[Record reported Quantity & Unit]**	**Quantity [Ask and record Quantity Sold & Unit]**	**Time & Price [Ask and record when the produce was sold and the price/kg when sold]**	**[Ask and record Quantity & Unit]**	**[Ask and record Quantity & Unit]**	**Others (Specify)**

4. Agricultural Outputs - secondary yield

a. Did you get any secondary yield from this field in addition to the crops listed above?

[Give examples of 'secondary yields' such as straw that might be used as feed for livestock]

[ ] YES [ ] NO

b. If 'YES', how much did you obtain in the last farming year?

[Ask and record quantity & unit - OR if sold income from it]

**Quantity**	**Unit**	**Income [if sold]**

5. Additional information about the field

[Use this space to record any additional information relevant to this field that are not captured in earlier sections]

can paying 4 global ecosystem services reduce poverty?
les paiements pour les services écosystèmiques globaux peuvent-ils réduire la pauvreté?

Work Package 6: Fanadihadiana mahakasika fambolena

Mahakasika ny fisy fanadihadiana

Asa	Daty	Ora nanombohana	Ora namaranana	Tompon'andraikitra	Fanamarihana
Fanadihadiana					
Famenoana ny fisy					
Marika fisy					
Fampidirana ny vokatry ny fanadihadiana					
Fanamarinana fa voadika ny fisy					

A. Famaritana ny tokantrano sy toerana fonenany

[Fanamarihana : Akotran'ny fanontaniana faha-6 sy faha-7 vao dia raiso avy amin'ny fanadihadiana teo aloha ary fenoy ny fanontaniana faha -1,2,3,4,5]

1. Anaran'ny TK & Marika & Isam-pianakaviana	*(Anarana)*	_______*(HHID)*______*Isa*
2. Anaran'ny vohitra & Marika	*(Anarana)*	*(VID)*
3. Anaran'ny fokontany & marika	*(Anarana)*	*(FID)*
4. Anaran'ny kaominina & Marika	*(Anarana)*	*(CID)*
5. Anaran'ny Distrika & Marika	*(Anarana)*	*(DID)*
6. Anaran'ny olona hadihadiana voalohany sy Marika	*(Anarana)*	*(PID)*
7. Anaran'ny olona hadihadiana faharoa & Marika	*(Anarana)*	*(PID)*
8. Toeram-ponenan'ny hadihadiana GPS	*(Lat)*	*(Lon)*
9. Alaviran'ny tanàna	*(Minitra)*	*(Km)*

Olona hadihadiana

[Fanamarihana: ny loham-pianakaviana no anaovana ny fanadihadiana, akotr'izay dia tokony hiresaka amin'ny olon-dehibe mety manana fanazavana hafa na manan-danja]

[Raha efa antitra na kilemaina ilay olona tompon-tany hanaovana fanadihadiana, dia afaka ataon'ny olona tanora ao an-tokantrano ny fandrefesana ny tany; ary atao ao an-tokantrano miaraka amin'ilay loham-pianakaviana ihany ny famenoana ny fisy]

[Mariho eto ny mombamomba ny olona hadihadiana raha tsy loham-pianakaviana]

B. Mahakasika ny tany hajariana

1. Mahakasika ireo tany ambolena

[Mety efa azo tany aloha ny valiny sasany amin'ireto fanontaniana manaraka ireto na izany aza anefa dia hamarino indray amin'ity fanadihadiana faharoa ity]

Nomeraon'ny tany	Anaran'ny toerana	Halavirany miala eo @ toeram-ponenana	velarany	karazany	fomba nahazoana ny tany [Raha 'savoka' ilay tany dia anontanio ihany koa raha fanany manokana na hiombonany amin'ny fianakaviana]	Nofaina/ Nindramina/ Novidiana	Vidiny/ antsipirihan'ny fifanarahana raha nofaina na nindramina na novidiana		nambolena tamin'ny taom-pambolena lasa teo ve?	taonan'ny tany	Fotoana hanajariana ny tany
				(1=tavirana tavy; 2=lemaka tavy; 3=tanimbary ; 4=tanimboly ; 5=savoka; 6=hafa (inona)	*(1=lova; 2= nofaina (hazavao); 3=novidiana (hazavao); 4=nindramina (hazavao); 5=nohajarian'ny tokan-trano, 6=hafa (inona))*	**[Raha nofaina na novidiana na nindramina ilay tany dia fantaro hoe iza no nahazoany azy, fianakaviana ve sa olonkafa] (1=fianakaviana; 2=hafa)**	*Karazana fifanarahana (vola/ zavatra)*	*Sandany*	*(nampiasaina tamin'ny taom-pambolena 2013/2014) (1=ENY 0=TSIA)*	**[Fenoy ito ao aorinan'ny fanontiana mahakasika ny tantaran'ilay tany ao amin'ny C]**	*(Raiso ny elanelan'ny fotoana nampiasaina azy farany sy amin'izao fotoana izao) [raha miala sasatra ilay tany amin'izao fotoana izao, anontanio na raiso hoe hafiriana ny fotoana hialan'ny tany sasatra]*

Fanamarihana

2. Mahakasika ny tavy

[Fanamarihana: Araho tsara ireo toromarika isaky ny fanontaniana, indrindra rehefa misy valin-teny ENY/TSIA dia mila arahina sy jerena ireo fanontaniana mety mifampitohy aminy]

a. Inona ny tena olana sedrainareo matetika amin'ny fambolena mahakasika ny tavy?

[Rehefa vita ny famelabelarana nataon'ilay olona hadihadiana dia tariho amin' ireto antony manaraka ireto izy raha sendra mbola tsy voateniny teo aloha. Alaharo araka ny lanjany (ireo telo lehibe ihany raisina raha mihoatra)

Antony	Asio 'X' raha io	Alaharo [3 voalohany]
tsy fahampian'ny olona miasa (na ny isan'ny afaka miasa ao an-trano/vola hanakaramana)		
tsy fahampian'ny fanafody		
tsy fahampian'ny masomboly		
tsy fahamasahan'ny tany		
tsy afaka manao teviala vaovao noho ny fandrarana ara-pomban-drazana		
tsy afaka manao teviala vaovao noho ny fandraran'ny fanjakana		
tsy afaka manao teviala vaovao noho ny tsy fisian'ny tany na noho ny fahabetsahan'ny mponina		

b. Inona ny olana tsy ahafahanareo mahazo vokatra tsara amin'ny fambolenareo?

[Rehefa vita ny famelabelarana nataon'ilay olona hadihadiana dia tariho amin' ireto antony manaraka ireto izy raha sendra mbola tsy voateniny teo aloha. Alaharo araka ny lanjany (ireo telo lehibe ihany raisina raha mihoatra)]

Antony	Asio 'X' raha io	Alaharo [3 voalohany]
tsy fahampian'ny olona hiasa (na ny isan'ny hiasa ao an-trano/vola hanakaramana)		
tsy fahampian'ny fanafody		
tsy fahampian'ny masomboly		
tsy fahamasahan'ny tany		
biby manimba voly (bibikely,vorona,)		
ahi-dratsy		
orana		

c. Manana tavy nohajariana tato anatin'ny 5 taona ve ianareo?

[ ] ENY >> jereo '3.'　　　[ ] TSIA >> jereo'd.'

d. Mieritreritra hanajary tavy hafa fanampiny ve ianareo?

[ ] ENY >> jereo 'e.'　　　[ ] TSIA >> jereo 'f.'

e. Raha ENY, inona no antony misakana anao tsy hanao izany ?

[Rehefa vita ny famelabelarana nataon'ilay olona hadihadiana dia tariho amin' ireto antony manaraka ireto izy raha sendra mbola tsy voateniny teo aloha. Alaharo araka ny lanjany (ireo telo lehibe ihany raisina raha mihoatra)]

Antony	Asio 'X' raha io	Alaharo [3 voalohany]
tsy misy tany azo vidiana		
tsy misy tany afaka hofaina		
tsy ampy ny olona afaka ampiasaiko hiasa izany tany vaovao izany		
tsy manam-bola hividianana na hanofaina tany		
tsy afaka manao teviala mba hahazoana tany hafa ajariana		
lasan'ny mpiavy daholo ny tany		
mitombo be ny isan'ny olona mampiasa tany		

f. Raha TSIA, inona no antony tsy hahafahanao hanajary tany hafa hanaovana tavy?

[Rehefa vita ny famelabelarana nataon'ilay olona hadihadiana dia tariho amin' ireto antony manaraka ireto izy raha sendra mbola tsy voateniny teo aloha. Alaharo araka ny lanjany (ireo telo lehibe ihany raisina raha mihoatra)]

**Antony**	**Asio 'X' raha io**	**Alaharo [3 voalohany]**
tsy misy tany azo vidiana		
tsymisy tany afaka hofaina		
tsy ampy ny olona afaka ampiasaiko hiasa izany tany vaovao izany		
tsy manam-bola hividianana na hanofaina tany		
tsy afaka manao teviala mba hahazoako tany vaovao		

3. Tanimbary

[Fanamarihana: Araho tsara ireo toromarika isaky ny fanontaniana, indrindra rehefa misy valin-teny ENY/TSIA dia mila arahina sy jerena ireo fanontaniana mety mifampitohy aminy]

a. Manana tanimbary ve ianao?

[ ]ENY >> jereo 'b.' [ ] TSIA >> jereo 'l.'

b. Manana tanimbary vao nohajariana tato anatin'ny telo taona ve ianareo?

[ ] ENY >> jereo 'c.' [ ] TSIA >> jereo 'e.'

c. Raha 'ENY', oviana? [mariho izay izy]

[ ] 2012 [ ] 2013 [ ] 2014

d. Inona avy ny fandaniana betsaka tamin'ny nanajarianao io tanimbary io tany amboalohany? >> jereo 'h.'

**Antony**	**Fandaniana**	
	Mpiasa [fitambaran'ny olona miasa]	*Sora-bola*
Fahazoana ilay tany		
Fanamboarana ny tanimbary (fandamahana, fanamboarana ny sisim-parihy,,,)		
Fotodrafitr'asa (fanamboarana lakan-drano, fitsinjarana rano,...)		
Hafa (inona)		
Hafa (inona)		
Hafa (inona)		
Hafa (inona)		

e.Efa nanitatra na nampitombo ny tanim-bary nananao ve ianao raha tato ato?

[ ] ENY >> jereo'h.' [ ]TSIA >> jereo 'f.'

f. Mieritreritra hanao izany ve ianao ao aoriana?

[ ] ENY >> jereo 'h.' [ ] TSIA >> jereo 'g.'

g. Inona ny antony tsy ahafahanao hanao izany?

[Rehefa vita ny famelabelarana nataon'ilay olona hadihadiana dia tariho amin' ireto antony manaraka ireto izy raha sendra mbola tsy voateniny teo aloha. Alaharo araka ny lanjany (ireo telo lehibe ihany raisina raha mihoatra)]

**Antony**	**Asio 'X' raha io**	**Alaharo [3 voalohany]**
tsy fisian'ny tany		
tsy fisian'ny olona hiasa		
tsy fahampian'ny ho enti-manana (vola hividianana/na hanofana)		
be rano loatra		
tsisy rano (maina)		
tsy fahampian'ny enti-manana hanangana foto-drafitr'asa (toha-drano,sns)		
tsy fahaiza-manao		

h. Inona no ambolenareo eo amin'ny tanim-barinareo?

[raha manana tanimbary maromaro izy dia raiso daholo ny zavatra amboleny]

[ ] varin-taona >> jereo'i.' [ ]vary in-2 [ ]vary in-3 [ ] vary & voly avotra (anarana)

[ ] hafa (inona)

i. Mieritreritra ny hanao karazana voly betsaka isan-taona ve ianareo?

[ ] ENY >> jereo 'j.' [ ] TSIA

j. Raha 'ENY', inona avy ireo antony tsy nahafahanao nanao izany?

[Rehefa vita ny famelabelarana nataon'ilay olona hadihadiana dia tariho amin' ireto antony manaraka ireto izy raha sendra mbola tsy voateniny teo aloha. Alaharo araka ny lanjany (ireo telo lehibe ihany raisina raha mihoatra)]

**Antony**	**Asio 'X' raha io**	**Alaharo [3 voalohany]**
tsy fisian'ny tany		
tsy fisian'ny olona hiasa		
tsisy rano (maina)		
tsisy fanafody		
hafanana		

k. Inona no mety sakana tsy ahafahanareo manatsara ny vokatra eny an- tanimbarinareo?

[Rehefa vita ny famelabelarana nataon'ilay olona hadihadiana dia tariho amin' ireto antony manaraka ireto izy raha sendra mbola tsy voateniny teo aloha. Alaharo araka ny lanjany (ireo telo lehibe ihany raisina raha mihoatra)]

**Antony**	**Asio 'X' raha io**	**Alaharo [3 voalohany]**
Tsy fahampian'ny olona hiasa		
Be rano loatra		
Tsisy rano		
Tsisy zezika		
Tsy fisian'ny foto-drafitr'asa hitsinjarana ny rano (barrages etc)		
Misy biby manimba voly		
Misy ahi-dratsy		

l. Inona avy ny antony tsy hananareo tanim-bary?

[Rehefa vita ny famelabelarana nataon'ilay olona hadihadiana dia tariho amin' ireto antony manaraka ireto izy raha sendra mbola tsy voateniny teo aloha. Alaharo araka ny lanjany (ireo telo lehibe ihany raisina raha mihoatra)]

**Antony**	**Asio 'X' raha io**	**Alaharo [3 voalohany]**
tsy fisian'ny tany		
tsy fahampian'ny olona hiasa		
tsy fahampian'ny enti-manana hividianana na hanofaina tanimbary		
be rano loatra		
tsisy rano		
tsy fahampian'ny enti-manana hanangana foto-drafitr'asa (toha-drano,sns)		
tsy fahaiza- manao		

m. Mieritreritra ny hanajary tanim-bary ve ianareo any aoriana?

[ ] ENY >> jereo sub-section '4.' [ ] TSIA >> jereo 'n.'

n. Inona ny tena antony mety hanakana anareo?

[Rehefa vita ny famelabelarana nataon'ilay olona hadihadiana dia tariho amin' ireto antony manaraka ireto izy raha sendra mbola tsy voateniny teo aloha. Alaharo araka ny lanjany (ireo telo lehibe ihany raisina raha mihoatra)]

**Antony**	**Asio 'X' raha io**	**Alaharo [3 voalohany]**
tsy fisian'ny tany		
tsy fahampian'ny olona hiasa		
Tsy fahampian'ny enti-manana hividiana na hanofana tanimbary		
be rano loatra		
tsisy rano		
tsy fahampian'ny enti-manana hanangana foto-drafitr'asa (toha-drano,sns)		
tsy fahaiza-manao		

4. Vokatra: Tavy vs Tanimbary

a. Iza amin'ireto no mamokatra kokoa raha jerena ny vokatra azo isaky ny Ha 1)?

[ ] Tavy (vao notevehina) [ ] Tavy (efa ela) [ ] Tanimbary

b. Iza amin'ireto no ahazoana tombony bebe kokoa raha jerena ny fandaniana mandritra ny taom-pambolena iray?

[ ] Tavy (vao notevehina) [ ] Tavy (efa ela) [ ] Tanimbary

C. Fiompiana (fandaniana sy vokatra azo)

1. Fiompiana[fandaniana sy vokatra azo]

	**isany[hanamarinana ny valiny azo nandritra ny fanadihadiana isan-tokantrano]**		**Fandaniana [nandritra ny 12 volana na fotoana voafaritra hanaovana fanadihadiana]**				**Vokatra azo [nandritra ny 12 volana na fotoana voafaritra hanaovana fanadihadiana]**		
	Fanadihadiana 1	**Fanadihadihana 2**	aiza no mitoby?	*Sakafo*	*fanafody*	***ffandaniana hafa[inona]***	*Natao sakafo*	*Namidy (biby fiompy)*	*Namidy (vokatra)*
Omby									
Kisoa									
Akoho									
Gana									
Gisa									
Ondry									
Bee hives									
Fish tanks/ponds									
Hafa [inona]									

D. Velon-tena hafa

1. Akotran'ny fambolena, inona ihany koa ny fidiram-bola hafa ao an-tokantranonareo nandritra ny taom-pambolena (2013/2014)? Oviana no tena nanaovanao izany ary ohatrinona ny vola azo?

Olona manao ilay asa	Loharanom-bola*	Misy hifandraisany mivantana na ankolaka amin'ny fiarovana ny ala ve?	Fotoana lany hanaovana ilay asa [omeo ny isan'ny andro na isan'ny volana]	Taha [isan'andro, isan-kerin'andro na isam-bolana]	Fitambaran'ny vola miditra
[Iza no olona nanao ilay asa : loham-pianakaviana ve sa tsia? Farito ny fifandraisany na ny fifandraisany amin'ny loham-pianakaviana, mariho ihany koa na olon-dehibe na ankizy, na lahy na vavy)]	[Raiso hoe inona ilay loharanom-bola araka ireo karazany etsy ambany, Raha toa ka misy loharanom-bola maromaro ny olona iray dia saraho isaky ny an-dalana vaovao]	[Raiso na " ENY" na "TSIA" ny Raiso raha misy fifandraisana mivantana na tsia ny loharano-bola sy ny faritra arovana na koa mifandray amin'ny resaka tontolo iainana]	[Kajio ary atambaro ny isan'ny andro niasany ho an'ireo mandray karama isan'andro, Ho an'ireo mandray vola isan-kerin'andro na isam-bolana dia kajio ihany koa ny isan'ny herin'andro sy ny volana]	[Raiso ny karama isan'andro na isam-bolana araka izay hanazavany azy . Ohatra, ___MGA/isan'andro; ____MGA/isam-bolana]	[Raha afaka milaza ilay olona anontaniana hoe mandray vola mitambatra" dia raiso; ho an'ireo hafa dia kajio ny fitambaran'ny vola azo rehetra]

* Karazana fidiram-bola vitsivitsy: 1=saraka an-tsaha (fambolena); 2=karama isan'andro (any amin'ny vohitra , oh., manadio, manao trano); 3=karama isan'andro (any amin'ny toerana hafa); 4=Mpitrandraka hazo(mpandidy hazo); 5= manao asa tanana, 6=asa birao (fanjakana na tsy miankina); 7=vola alefan'ny olona ao an-trano. Ho an'ny ankohatra ireo dia raiso mazava tsara .

1=vady(ofisaly na Tsia); 2=zanaka; 3=zanam-bady; 4=zafikely; 5=RAR; 6=Rafozana; 7=Rahalahy/anambavy; 8=zaodahy/zaobavy; 9=dadatao/nenintoa; 10=zanaka anambavy/anadahy; 11=zaza nat-sagana; 12=havana hafa; 13=Tsisy fifandraisana

E.Fanontaniana mahakasika teviala

[Fanamarihana: tsara raha anontaniana any amin'ny faran'ny fanadihadiana mba hialana amin'ny fihafahafan'ilay olona miresaka?]

1. Manana fahafahana hanao teviala ve ianao na ny tokantrano misy anao ety amin'ity toerana na tanana misy anareo

[ ] ENY >> jereo Q 2. [ ] TSIA >> jereo Q 3.

2. Izay fahafahana manao teviala izay ve dia voafetra amin'ny faritra iray? Jereo 3.

[ ] ENY [ ] TSIA

3. Raha toa ka azo atao ny manao Teviala ety. Iza no manana fahefana hanome alalana hanao izany? Aiza ary isaky ny oviana no afaka manao izany ny tokantranonareo? (oh. Tangalamena, Ray amandrenin'ny tanana)? [Raiso eto amin'ity tabilao ambany ity raha misy fanazavana fanampi-ny)]

**Fotoana nanaovana ilay teviala**	**Rafitra manome alalana**	**Aiza no afaka manao ilay teviala?**	**Amin'ny fotoana inona no afaka manao teviala?**	**Raha tsy manao teviala intsony amin'izao fotoana izao dia ino-na ny antony?**
[Raiso raha fahafahana manao teviala taloha na amin'izao fotoana izao]	[anontanio ireo karazana rafitra na ara-dalana na tsy ara-dalana]	[anontanio na ny toera-na na ny karazana tany azo anaovana izany (tanim-pokonolona,tanim-panjakana,sns)]	[anontanio eto hoe amin'ny fotoana inona no ahafahahana manao teviala sy ny fepetra rehetra mifanaraka izany]	[Anontanio ary raiso hoe aiza no mihatra ilay fandrarana ary iza no mametraka sy manara-maso ny fanaja-na izany]

[Raiso eto raha misy fanazavana fanampiny mahakasika ny fanaovana teviala]

Work Package 6: Fandihadiana mahakasika ny fambolena FID: ________ HHID: ________

[Manova fisy vaovao isaky ny tany iray]

A. Ny tokony ho fantatra mahakasika ny tany sy ny olona hadihadiana

(Raha misy amin'ireto efa azo tamin'ny fanadihadiana fototra teo aloha dia avereno hamarinana ihany eto)

1. Nomeraon'ny tany (avy amin'ny fisy voalohany)	
2. Toerana misy ilay tany (fiantson'ny olona eny an-toerana na ny fomba famaritany ilay tany)	
3. Voasoratra ara-dalàna amin'ilay tokantrano ve ilay tany(*vita borne)?*	
4. Afaka faritanao mazava ve izany (oh., fananana iombonana, fanana manokana, fahefana ara-pomban-drazana)	
5. Namboly tamin'io tany io ve ilay tokantrano tamin'ny taom-pambolena 2013/2014?	
6. Raha tsia,misy tokan-trano hafa ve manana fahefana hampiasa ity tany ity?	
7.Raha 'ENY', iza?	
**[Note: Raha tsy nambolena tao anatin'ny fotoana vitsivitsy, dia refeso na vinavinao ny haben'ny tany ary raiso ny hamaron'ny zava-maniry mandrakotra sy ny karazan'ireo voly natao teo amin'ilay tany teo aloha]**	
8. Niara nijery ilay tany ve ny olona mpanadihady sy ilay olona hadihadiana? (1=ENY; 0=TSIA)	
9. Voatsidika ilay tany fa saingy sarotra ny nandrefy azy noho ny habetsaky ny bozaka sy ahitra eo amin'ilay tany (1=ENY; 0=TSIA)	

B. Famaritana ny velaran'ilay tany sy ny toerana misy azy

1. Famintinana ireo an-tontanisa azo tamin'ny GPS

**GPS (mari-drefy ampiasaina)**	**Isa laharan'ny ireo teboka nalaina tao GPS (oh. 37-43, or 37, 39-43)**

2. Raha kely ny velaran-tany dia raiso araky ity tabilao ity ny mari-drefy entina mamaritra ny velaran-tany.[ampiasao arak'izay ilaivana azy ny an-dalana amin'ny fandraisana ny teboka]

	**Refin'ireo zoro (Compas ao GPS)**	**Refin'ny elanelana nangalana ny teboka tsirairay (ataovy isan'ny dingana mitovy no angalana ny zoro)**
teboka fanombohana		
1		
2		
3		
4		
5		
6		
7		
8		
9		
10		
teboka famaranana tokony hifandraika amin'ilay fanombohana		

3. Faminavinana ny haben'ny tany tsy voatsidika

[Raha tena lavitra loatra ilay tany dia azo vinavinain'ilay olona hadihadiana ihany ny refiny, fa raha sarotra refesina noho ny ahitra sy kirihitra dia afaka vinavinain'ny mpanadihady sy ny olona hadihadiana ihany raha hoarina amin'ny tany efa voarefy teo aloha]

**Tany nentina nanoharany ny habehany tany hovinaniana**	**Refy ankapobeany tany rehefa noharana tamin'ilay tany voarefy teo aloha (%)**

C. Tantaran'ny tany

1. Nanomboka hatramin'ny nanajariana ilay tany voalohany: Oviana no notevesina voalohany ilay tany? ary iza no nanao izany?[Ampiasao ireo toe-javatra tena nanamarika ilay fotoana (rivo-doza, fiovany fitondrana (Prezida), fanjanahan-tany,sns)]

**[(Marika ho an'iry mamaly : -98=tsy mahafantata; -99=tsy te hamaly; -100=tsy voanontany (lazao fa maninona)]**	**[ampiasao ireto handraisana ny antsimpirihan-javatra hafa lazain'ilay olona hadihadihana]**		
a. Oviana no nanomboka nampiasa ilay tany ilay tokantrano?			
b. Ianareo ihany ve no nanajary ilay tany voalohany? (raha 'ENY', iza?)			
c. Raha TSIA, iza no nanao izany?			
d. Inona ny fifandraisan'nilay olona nanajary voalohany sy ny loham-pianakaviana			
e. Ahoana ny fomba nahazoan'ilay tokan-trano ilay tany?	**[Ito dia efa hita amin'ny fanadihadiana mahakasika ny fambolena, ao amin'ny section B fa hanamarino eto raha diso na tsia izay voalaza tany aloha]**		
f. Taona nanajariana voalohany ilay tany			
g. Raha tsy mahatadidy ilay olona dia raiso ny elanelan'ny fotoana mety nanaovana izany			
h. Ilay olona nitevy ilay ala voalohany ve nahazo alalana tamin'ny fanjakana sa tsy maint-sy nanefa honitra			
i. Raha 'ENY', omeo ny antsipirihany			

2. Antsipirihany hafa mahakasika ilay tany na ny voly nifanesy teo

[Ampiasao ity handraisana ireo fanazavana fanampiny mahakasika ilay toerana, izay hita fa manan-danja na ilaina amin'ny fanadihadiana]

3. Voly natao teo amin'ilay tany sy ny elanelam-potoana nanaovana izany

a. Atomboy amin'ny taom-pambolena voa aingana indrindra, ary farito koa ireo voly rehetra natao isakin'ny taom-pambolena.

	0 **Taom-pambolena farany (_________AD)**	**_________ AD**	**_________ AD**	**_________ AD**	**_________ AD**		
Inona avy ireo karazana voly natao teo amin'ilay tany(izay nataon'ilay tokantrano anontaniana ihany)							

b. Araka ny fijeriny sy ny fitadidiny dia mety impiry no efa nampiasaina ity tany ity talohan'ny nanaovan'ilay tokantrano fambolena teo?

c.Araka ny fijeriny sy ny traik'efa efa ananan'ilay olona hadihadiana, efa mety niova ve ny halavan'ny fotoana hialan'ilay tany sasatra, nanomboka hatramin'ny voalohany nanajariana azy?

Halavan'ny fotoana hialan'ny tany sasatra teo aloha	
Halavan'ny fotoana hialan'ny tany sasatra amin'izao fotoana izao	

D. Fandaniana sy fampiasana ny vokatra azo avy amin'ny fambolena

1. Fandaniana makasika ny fambolena - olona miasa: firy ny isan'ny olona niasa ity tany ity tany ity nandritra ny taom-pambolena 2013/2014?

[Fanamarihana: Raiketo eo faritra natokana hoan'izany valin-teny azo ohatra'Tavy' dia avelao ny faritra 'Tanimboly' OR 'Tanimbary']

**Tavy**	**Fotoana**	**Olona niasa tao an-tokantrano**			**Olona nalaina avy any ivelany**			**Fandaniana hafa**
		Lahy	*Vavy*	*Ankizy*	Mpiasa	*Karazana tambiny* ***[Raiso na vola na zavatra]***	*Ohatrinona?* ***[Raiso ny fatrany raha zavatra/ny sandany raha vola]***	**[Raiso raha misy fandaniana hafa mahakasika ny olona nampiasaina]**
Kapakapa		_____ isa / _____ andro	_____isa / _____ andro	_____ isa / _____ andro	_____ isa / _____ andro			
Mandoro		_____ isa / _____ andro	_____isa / _____ andro	_____ isa / _____ andro	_____ isa / _____ andro			
Mamboly		_____ isa / _____ andro	_____isa / _____ andro	_____ isa / _____ andro	_____ isa / _____ andro			
Miava [1]		_____ isa / _____ andro	_____isa / _____ andro	_____ isa / _____ andro	_____ isa / _____ andro			
Miava [2]		_____ isa / _____ andro	_____isa / _____ andro	_____ isa / _____ andro	_____ isa / _____ andro			
Miandry Fody		_____ isa / _____ andro	_____isa / _____ andro	_____ isa / _____ andro	_____ isa / _____ andro			
Misangom-bary		_____ isa / _____ andro	_____isa / _____ andro	_____ isa / _____ andro	_____ isa / _____ andro			
Mitango katsaka		_____ isa / _____ andro	_____isa / _____ andro	_____ isa / _____ andro	_____ isa / _____ andro			
Mitango tsaramaso		_____ isa / _____ andro	_____isa / _____ andro	_____ isa / _____ andro	_____ isa / _____ andro			
Mitaona vary		_____ isa / _____ andro	_____isa / _____ andro	_____ isa / _____ andro	_____ isa / _____ andro			
Mively vary		_____ isa / _____ andro	_____isa / _____ andro	_____ isa / _____ andro	_____ isa / _____ andro			
Hafa (inona)		_____ isa / _____ andro	_____isa / _____ andro	_____ isa / _____ andro	_____ isa / _____ andro			
**Tanimboly**								
Miava								
Taillage		_____ isa / _____ andro	_____isa / _____ andro	_____ isa / _____ andro	_____ isa / _____ andro			
Maka vokatra [____________]		_____ isa / _____ andro	_____isa / _____ andro	_____ isa / _____ andro	_____ isa / _____ andro			
Maka vokatra [____________]		_____ isa / _____ andro	_____isa / _____ andro	_____ isa / _____ andro	_____ isa / _____ andro			
**Tanimbary**								
Mamadika bainga/benja		_____ isa / _____ andro	_____isa / _____ andro	_____ isa / _____ andro	_____ isa / _____ andro			
Mampiditra rano		_____ isa / _____ andro	_____isa / _____ andro	_____ isa / _____ andro	_____ isa / _____ andro			
Miosy/Mamakivaky		_____ isa / _____ andro	_____isa / _____ andro	_____ isa / _____ andro	_____ isa / _____ andro			
Planage		_____ isa / _____ andro	_____isa / _____ andro	_____ isa / _____ andro	_____ isa / _____ andro			
Manetsa		_____ isa / _____ andro	_____isa / _____ andro	_____ isa / _____ andro	_____ isa / _____ andro			
Miava [1]		_____ isa / _____ andro	_____isa / _____ andro	_____ isa / _____ andro	_____ isa / _____ andro			
Miava [2]		_____ isa / _____ andro	_____isa / _____ andro	_____ isa / _____ andro	_____ isa / _____ andro			
Miandry Fody		_____ isa / _____ andro	_____isa / _____ andro	_____ isa / _____ andro	_____ isa / _____ andro			
Mijinja		_____ isa / _____ andro	_____isa / _____ andro	_____ isa / _____ andro	_____ isa / _____ andro			
Mitaona vary		_____ isa / _____ andro	_____isa / _____ andro	_____ isa / _____ andro	_____ isa / _____ andro			
Mively vary		_____ isa / _____ andro	_____isa / _____ andro	_____ isa / _____ andro	_____ isa / _____ andro			
Hafa (inona)		_____ isa / _____ andro	_____isa / _____ andro	_____ isa / _____ andro	_____ isa / _____ andro			
Hafa (inona)		_____ isa / _____ andro	_____isa / _____ andro	_____ isa / _____ andro	_____ isa / _____ andro			

2. Fandaniana mahakasika ny fambolena- fitaovana na fandaniana hafa

Voly	Fandaniana										
	Masomboly		*zezika azo avy amin'ny fiompiana*		*zezi-bazaha*		*fanafody*		*biby*	fanofana fitaovana	*fitaovana hafa*
	**Lanjany sy mari-drefy [anontanio ny lanjany sy ny refy ampiasaina]**	**sandany [raiso ny vidiny sy ny marika ampiasainy]**	**Anontanio raha mampiasa zezika mineraly? [Raiso na 'ENY' na 'TSIA']**	**Raha 'ENY', ampy ve ny zezika azony avy amin'ireo fiompiany ireo**	**Lanjany & mari-drefy [Anontanio ny lanjany sy mari-drefy]**	**sandany [raiso ny vidiny/mari-drefy]**	**Lanjany & mari-drefy [Anontanio ny lanjany sy mari -drefy]**	**sandany [raiso ny vidiny/mari-drefy]**	**[Raha nampiasa omby nandritra ny fotoana nikarakarana ny tany dia ohatrinona ny hofany]**	**[Raha nisy fitaovana hafa nampiasaina na nofaina dia ohatrinona ny hofan'izany]**	**[Raiso eto izay fandaniana hafa mahakasika ny asa natao teo amin'ilay tany]**

3. Fampiasana ny vokatra - vokatra azo,ny lanjan'ny nohanina, ny namidy,

Voly	Fampiasana ny vokatra								Hafa (inona)
	Fitambaran'ny vokatra azo	*Nanao ahoana ny vokatra azo tamin'ity taompambolena ity raha oharina amin'ny vokatra azo amin'ny ankapobeny teto?*	*Raha mifanalavitra amin'ny vokatra azo matetika dia mety eo amin'ny firy eo eo ny elanelany azo tamin'ity taompambolena ity sy ny amin'ny ankapobeny?*	*Lany ao antokantrano*	*Amidy*		*Nandoavana zavatra*	*Masomboly*	
	**[Raiso ny lanja sy ny mari-drefy ampiasaina]**	**[Ampio ilay olona hanome laharan'isa 5: 1= tena ambany, 2=ambany, 3=mitovitovy, 4=ambony, 5=teny ambony]**	**[raiso eto ny fiovany vokatra azo raha natao fanoharana tamin'ny vokatra azo amin'ny ankapobeany %]**	**[Raiso ny lanja sy ny mari-drefy ampiasaina]**	**Lanjany [Raiso ny lanjan'ny namidy sy ny mari-drefy ampiasaina]**	**Fotoana sy sandany [Anontanio hoe oviana no nivarotana azy sy ny vidiny/kg]**	**[Raiso ny lanja sy ny mari-drefy ampiasaina]**	**[Raiso ny lanja sy ny refy ampiasaina]**	

4. Fampiasana ny vokatra - vokatra miakatra hafa tsy novolena

a. Mbola misy vokatra hafa alainareo amin'ny tanimboly ve ankoatra ireo vokatra voalaza etsy ambony ireo?

[Ataovy ohatra amin'izany vokatra hafa izany ny vilina ho an'ny biby fiompy]

[ ] ENY [ ] NO

b. Raha 'ENY', firy ny habetsany azonareo tamin'ny taona lasa?

[Anontanio ny lanjany sy ny mari-drefy ampiasainy - na ny vola azo taminy raha namidy]

Lanja	Mari-drefy	Vola azo[raha namidy]

5. Fanamarihana mahakasika ny vokatra azo

[Ampiasao ity banga ity handraisana izay fanazavana hafa mahakasika ny vokatra ankoatra izay rehetra voalaza etsy ambony]

